# Supplementary material for: USP15 Deubiquitinase Safeguards Hematopoiesis and Genome Integrity in Hematopoietic Stem Cells and Leukemia Cells
Source: Cell Rep. 2020 Dec 29;33(13):108533. doi: 10.1016/j.celrep.2020.108533 (PMC7788286; doi:10.1016/j.celrep.2020.108533)
Supplement: Document S2. Article plus Supplemental Information [file mmc5.pdf]

Cell Reports

Article

USP15 Deubiquitinase Safeguards Hematopoiesis and Genome Integrity in Hematopoietic Stem Cells and Leukemia Cells

Graphical Abstract

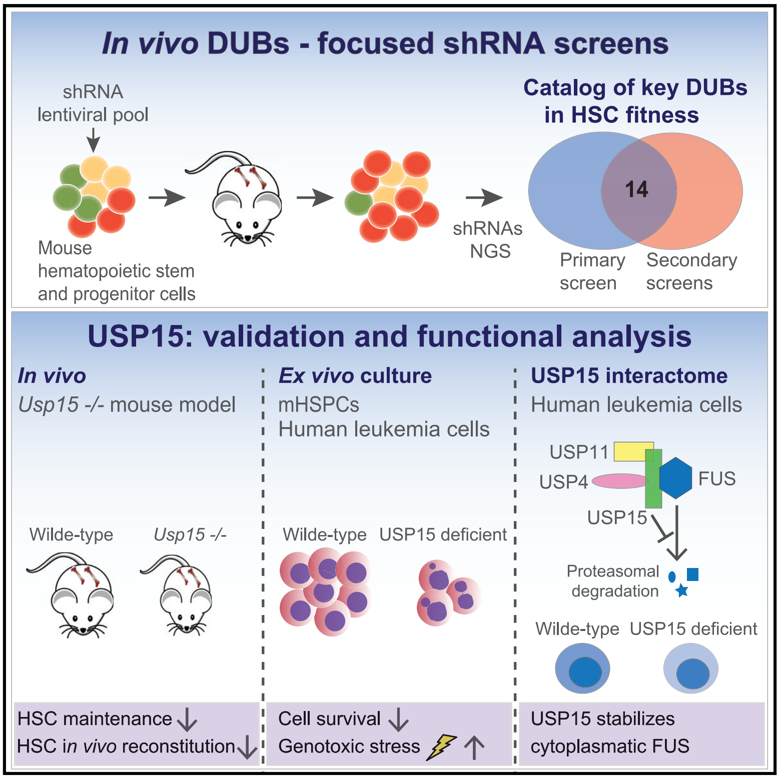

Highlights

- *In vivo* shRNAs screens for deubiquitinases identify regulators of murine hematopoiesis
- *Usp15* deletion compromises HSC maintenance and reconstitution potential *in vivo*
- USP15 loss affects genome integrity and growth of mHSPCs and human leukemia cells
- In human leukemia cells, USP15 stabilizes its interactor, FUS, a DNA repair factor

Authors

Paul van den Berk, Cesare Lancini, Carlos Company, ..., Maarten van Lohuizen, Gaetano Gargiulo, Elisabetta Citterio

Correspondence

gaetano.gargiulo@mdc-berlin.de (G.G.), elisabetta.citterio@gmail.com (E.C.)

In Brief

Van den Berk et al. use unbiased *in vivo* RNAi screens targeting deubiquitinases in mouse hematopoietic stem and progenitor cells. This study underscores the importance of deubiquitinases in hematopoietic stem cell function and reveals the role of USP15 in preserving genome integrity in normal and transformed hematopoietic cells.

van den Berk et al., 2020, Cell Reports *33*, 108533
December 29, 2020 © 2020 The Author(s).
https://doi.org/10.1016/j.celrep.2020.108533

CellPress

Cell Reports

CellPress
OPEN ACCESS

Article

USP15 Deubiquitinase Safeguards Hematopoiesis and Genome Integrity in Hematopoietic Stem Cells and Leukemia Cells

Paul van den Berk,[1] Cesare Lancini,[2,8] Carlos Company,[3,8] Michela Serresi,[3] Maria Pilar Sanchez-Bailon,[3] Danielle Hulsman,[2,7] Colin Pritchard,[4] Ji-Ying Song,[5] Matthias Jürgen Schmitt,[3] Ellen Tanger,[2] Oliver Popp,[6] Philipp Mertins,[6] Ivo J. Huijbers,[4] Heinz Jacobs,[1] Maarten van Lohuizen,[2,7] Gaetano Gargiulo,[3,9,*] and Elisabetta Citterio[2,7,9,10,*]

[1]Division of Tumor Biology and Immunology, the Netherlands Cancer Institute, Plesmanlaan 121, Amsterdam 1066 CX, the Netherlands
[2]Division of Molecular Genetics, the Netherlands Cancer Institute, Plesmanlaan 121, Amsterdam 1066 CX, the Netherlands
[3]Max-Delbrück-Center for Molecular Medicine (MDC), Robert-Rössle-Str. 10, 13092 Berlin, Germany
[4]Transgenic Core Facility, Mouse Clinic for Cancer and Aging (MCCA), the Netherlands Cancer Institute, Plesmanlaan 121, Amsterdam 1066 CX, the Netherlands
[5]Division of Experimental Animal Pathology, the Netherlands Cancer Institute, Plesmanlaan 121, Amsterdam 1066 CX, the Netherlands
[6]Proteomics Platform, Max Delbrück Center for Molecular Medicine in the Helmholtz Association and Berlin Institute of Health, Robert Rössle Strasse 10, 13125 Berlin, Germany
[7]ONCODE Institute, Utrecht, the Netherlands
[8]These authors contributed equally
[9]Senior author
[10]Lead Contact
*Correspondence: gaetano.gargiulo@mdc-berlin.de (G.G.), elisabetta.citterio@gmail.com (E.C.)
https://doi.org/10.1016/j.celrep.2020.108533

SUMMARY

Altering ubiquitination by disruption of deubiquitinating enzymes (DUBs) affects hematopoietic stem cell (HSC) maintenance. However, comprehensive knowledge of DUB function during hematopoiesis *in vivo* is lacking. Here, we systematically inactivate DUBs in mouse hematopoietic progenitors using *in vivo* small hairpin RNA (shRNA) screens. We find that multiple DUBs may be individually required for hematopoiesis and identify ubiquitin-specific protease 15 (USP15) as essential for HSC maintenance *in vitro* and in transplantations and *Usp15* knockout (KO) mice *in vivo*. USP15 is highly expressed in human hematopoietic tissues and leukemias. USP15 depletion in murine progenitors and leukemia cells impairs *in vitro* expansion and increases genotoxic stress. In leukemia cells, USP15 interacts with and stabilizes FUS (fused in sarcoma), a known DNA repair factor, directly linking USP15 to the DNA damage response (DDR). Our study underscores the importance of DUBs in preserving normal hematopoiesis and uncovers USP15 as a critical DUB in safeguarding genome integrity in HSCs and leukemia cells.

INTRODUCTION

Hematopoietic stem cells (HSCs) have the unique properties of self-renewal and multilineage potential, giving rise to daughter stem cells and committed progenitors, thereby achieving lifelong hematopoiesis. This is accomplished by maintenance of a homeostatic balance among HSC quiescence, self-renewal, and differentiation (de Haan and Lazare, 2018; Laurenti and Göttgens, 2018; Morrison and Spradling, 2008). Perturbation of this balance and replication stress can cause stem cell failure or transform normal HSCs and progenitors into disease-initiating leukemic stem cells (LSCs) (Flach et al., 2014). Understanding HSC and bone marrow (BM) homeostasis is therefore essential for understanding mechanisms controlling diseases and ultimately targeting LSCs (Warr et al., 2011).

The 76-amino-acid molecule ubiquitin is conjugated to proteins as a monomer (mono-ubiquitination) or in the form of ubiquitin chains (poly-ubiquitination) through the sequential action of E1, E2, and E3 enzymes (Yau and Rape, 2016). Deubiquitinating enzymes (DUBs; also referred to as deubiquitylating enzymes or deubiquitinases) reverse substrate ubiquitination, thereby critically regulating ubiquitin-mediated signaling pathways, including protein homeostasis and DNA repair (Mevissen and Komander, 2017). Consequently, deregulation of DUBs is implicated in human pathologies, such as cancer and neurodegenerative, hematological, and infectious diseases (Heideker and Wertz, 2015).

The human genome encodes ~100 DUBs, which are grouped into seven families based on structural properties (Haahr et al., 2018; Kwasna et al., 2018; Mevissen and Komander, 2017). We reported that ubiquitin-specific protease 3 (USP3) protects

This is an open access article under the CC BY license (http://creativecommons.org/licenses/by/4.0/).

mouse HSC function through modulation of the ubiquitin-dependent DNA damage response (DDR), a critical genome maintenance pathway (Lancini et al., 2014). This is in line with a proper DDR being crucial to HSC function (Bakker and Passegué, 2013; Biechonski et al., 2017). Numerous DUBs control ubiquitin-dependent DDR (Citterio, 2015; Lukas et al., 2011; Nishi et al., 2014; Schwertman et al., 2016), and DUB deregulation contributes to altered HSC homeostasis and human blood diseases (Adorno et al., 2013; Dey et al., 2012; Gu et al., 2016).

Functional analysis of HSCs within their physiological environment is more likely to result in finding modulators potentially relevant in disease (Morrison and Spradling, 2008; Schepers et al., 2015). Unbiased, functional genomic approaches by short hairpin RNAs (shRNAs) have demonstrated the power of forward RNAi screens in dissecting functional aspects of both normal (Cellot et al., 2013; Galeev et al., 2016) and leukemic HSCs (Zuber et al., 2011). Using lentiviral-based libraries (Gargiulo et al., 2014; Serresi et al., 2018), pooled *in vivo* screening approaches in early murine hematopoietic precursors led to the identification of critical factors limiting normal HSC self-renewal (Wang et al., 2012), as well as of determinants of malignant hematopoiesis (Miller et al., 2013; Puram et al., 2016).

While recent gene-centric approaches connected DUBs to HSC maintenance (Citterio, 2015), a comprehensive understanding of DUB biological functions in hematopoiesis and leukemia is missing. DUBs are poorly represented in *in vivo* screens (Wang et al., 2012), and *in vitro* functional approaches for DUBs in cancer cell lines were hypothesis driven (Nishi et al., 2014). In this study, we individually depleted all DUB genes using *in vivo* RNAi screens in mouse hematopoietic precursors, with the aim of ranking the most relevant DUBs required for normal and malignant hematopoiesis. We uncovered multiple DUBs as putative regulators of hematopoietic precursors activity and highlighted USP15 as a determinant of hematopoiesis *in vivo* and its role in preserving genome integrity, with potential implications for combinatorial treatments in leukemia.

RESULTS

In Vivo RNAi Screens for DUBs Identify DUB Regulators of HSPC Activity

To identify DUB determinants of mouse HSC activity, we performed pooled *in vivo* RNAi screens using adult murine hematopoietic stem and progenitor cells (HSPCs, mHSPCs) in a BM transplantation setting (Figure 1A). We generated a custom pool of 508 lentiviral shRNAs vectors potentially targeting all annotated mouse orthologs of human DUBs (~100) (Mevissen and Komander, 2017). This primary library contained three to six shRNA vectors per gene, selected from the shRNA library developed by the RNAi Consortium (TRC) at the Broad Institute (Open Biosystem) (Tables S1 and S2). Since statistical representation of shRNA libraries is critical for success in *in vivo* screening, we used the full library in a primary screen and divided the library into two sub-pools (DUB1 and DUB2 sub-libraries) used in secondary screens (Figure 1B). To perform qualitative controls, we included in each library shRNAs targeting known HSCs regulators as positive controls (Park et al., 2003; Vasanthakumar et al., 2016; Wang et al., 2012).

Freshly isolated lineage-negative (Lin^-) BM cells were transduced with the titered shRNAs pooled library (MOI < < 1), selected with puromycin, and subsequently injected into lethally irradiated mice (Figure 1A). In this limited time window, Lin^- cells were maintained *in vitro* in the presence of HSC cytokines in conditions known to preserve and enrich for stem cells/early progenitors (Ye et al., 2008). Indeed, early progenitors were maintained during transduction, as gauged by the enrichment of the $Lin^-c\text{-}Kit^+Sca1^+$ (LSK) cells in fluorescence-activated cell sorting (FACS) analysis (Figure S1A). Notably, the transduced cell culture also retained phenotypic HSCs, which was assessed by the HSC SLAM (signaling lymphocyte activation molecule) surface marker $CD150^+$ that is expressed on cells endowed with an immature phenotype and reconstitution potential (Christensen and Weissman, 2001; Kiel et al., 2005; Yeung and So, 2009) (Figure S1A). Transduced Lin^- cells were mixed 1:1 with total BM cells from CD45.1 mice (Figure 1A). To ensure optimal representation of the shRNA library, we injected a minimum of 1×10^6 Lin^- transduced cells per mouse, aiming for least at a predicted 2,000-fold library representation per animal, which is estimated to be sufficient to control for grafting efficiency and stochastic drifts (Gargiulo et al., 2014; Serresi et al., 2018).

We allowed cells to engraft recipient animals and harvested blood, BM, and spleen from recipient mice at 4 weeks post-transplantation (wpt). We chose a 4-week time point as readout based on experimentally determined parameters. First, we verified that 4 weeks is a sufficiently long period of time to allow assessment of potential phenotypic defects of the murine progenitors during the acute proliferative phase. This included both expansion and depletion, thereby enabling us to identify genes regulating either quiescence or proliferation. Second, 4 weeks is a time frame consistent with polyclonal engraftment and insufficient to allow manifestation of compensatory mechanisms and HSC clonality issues. In fact, in long-term engraftment experiments (4–6 months), only a small number of HSCs contribute to most cellular output (Naik et al., 2013). In our experiments, we observed measurable grafting in recipients and the generation of donor-derived B cells in the spleen of transplanted recipients (Figures S1B and S1C). This supports the 4-week time point as being sufficient to enable the screen while limiting HSC clonal expansion.

FACS analysis of BM, circulating blood cells, and splenocytes showed successful engraftment of the transduced Lin^- cells, with an average of 50% contribution in the BM (Figures 1C, S1B, and S1C). To assess the relative representation of each shRNA *in vivo*, we then performed parallel next-generation sequencing of PCR-amplified shRNA sequences from genomic DNA in the following conditions: (1) *in vivo* hematopoietic precursors and differentiated cells, isolated at 4 wpt from the BM (Lin^- cells) or the spleen ($CD43^-$, $CD45.2^+$, $CD19^+$, $CD220^+$ B cells), respectively, of recipients; and (2) control transduced Lin^- cells immediately before injection (input, or time 0 [T0]), as well as the plasmid library. Sequencing of individual samples revealed that individual shRNA abundance in transduced Lin^- (T0) correlated well with the hairpin reads in the plasmid library, supporting efficient transduction *in vitro* (R^2 = 0.69; Figure S2A). Importantly, more than 97% of the hairpins could be identified in the transduced Lin^- (T0) and more than 89% were retrieved *in vivo* in

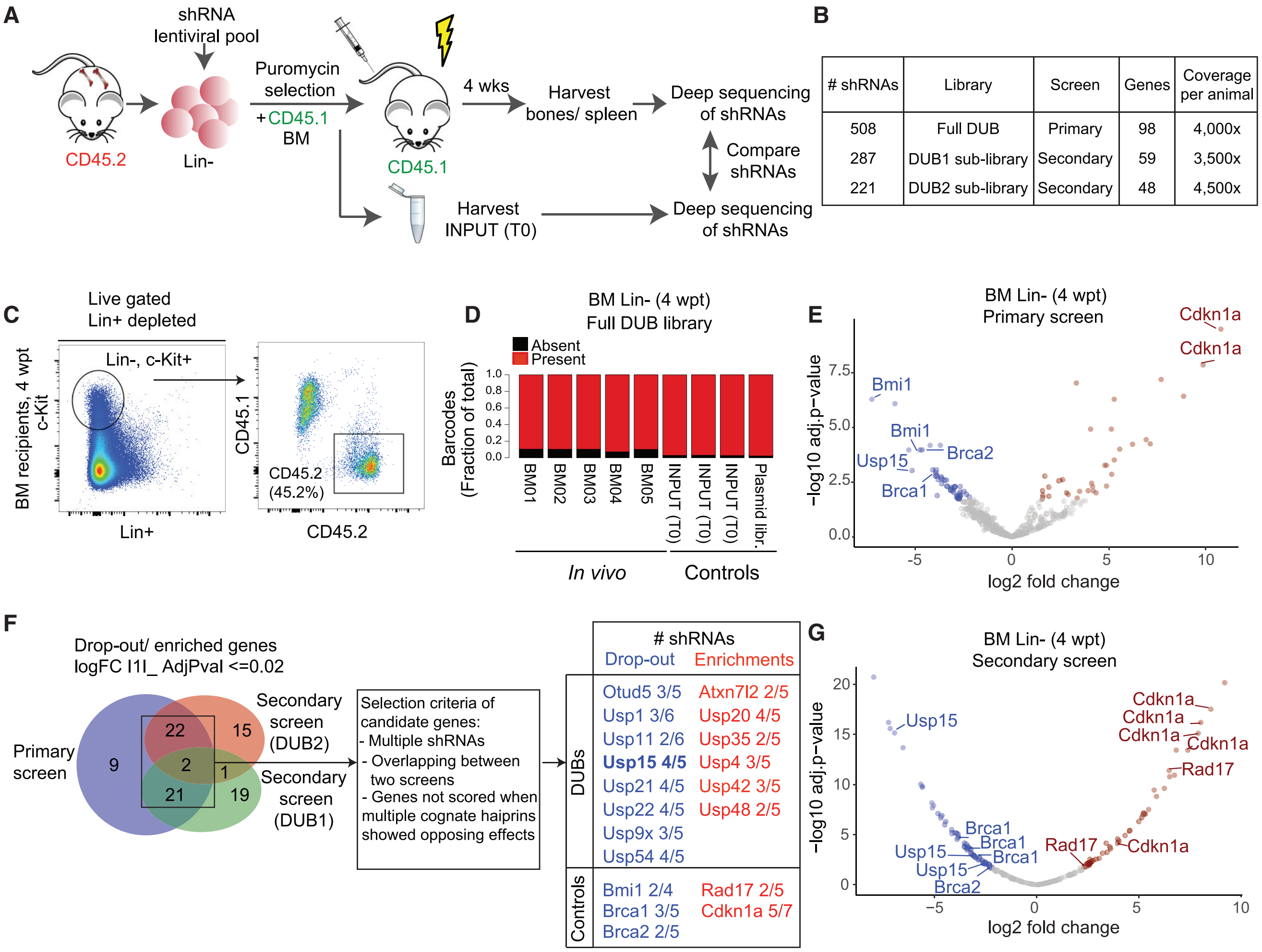

Figure 1. Pooled *In Vivo* RNAi Screen Identifies Candidate DUBs Effectors of Hematopoietic Stem and Progenitor Cell (HSPC) Activity

(A) Overview of the DUB RNAi screens *in vivo*.

(B) Primary and secondary screens parameters.

(C) Representative FACS profiles of Lin^- fraction purified from recipients at 4 wpt and analyzed for chimerism.

(D) Consolidated fraction of shRNAs retrieved *in vivo* in Lin^- cells at 4 wpt and controls.

(E) Volcano plot depicting the log2 fold change (FC) in the BM of recipients of all hairpins used in the primary screen, normalized across five replicates.

(F) Venn diagram depicting significantly differentially represented genes overlapping between the primary and secondary screens.

(G) Volcano plot depicting the log2 FC in the BM of recipients of all hairpins used in the secondary screen (DUB2 sub-library), normalized across seven replicates. Significantly (adjusted p $\leq$ 0.02) dropout (log2 FC $\leq$ 1, blue), and enriched (log2FC $\geq$ 1, red) shRNAs are shown in (E)–(G).

See also Figures S1 and S2.

purified Lin^- cells from each recipient mouse (4 wpt). We concluded that a significant proportion of the initial library complexity is maintained *in vitro* and *in vivo* (Figures S2A and 1D).

Principal-component analysis (PCA) showed that the five *in vivo* BM samples were more similar to each other and were distinct from the input cells before injection, and limited variance between the individual samples was found (Figures S2B and S2C). Moreover, a positive correlation was found between the relative representation of shRNAs retrieved from the BM to the ones retrieved from the spleen (R^2 = 0.668) (Figure S2D; Table S3).

Next, we performed a differential enrichment analyses on the *in vivo* and control samples. Among the top hits, we found genes relevant to HSC biology to be either enriched (involved in cell cycle restriction) or depleted (supporting self-renewal), including our positive controls. Consistent with the requirement for Bmi1 in adult HSC self-renewal (Park et al., 2003), two out of the four shRNAs targeting Bmi1 showed significant dropout (>20-fold) in Lin^- cells *in vivo* (Figure 1E; Table S3). DNA repair genes BRCA1 and BRCA2/FANCD1 were also highly depleted with at least one shRNA per gene, in line with their role in HSC survival (Navarro et al., 2006; Vasanthakumar et al., 2016). Consistent with a role in cell-cycle restriction (Wang et al., 2012), two shRNAs for the cell-cycle inhibitor *Cdkn1a* were enriched (Figure 1E). Notably, DUBs with established importance in HSC

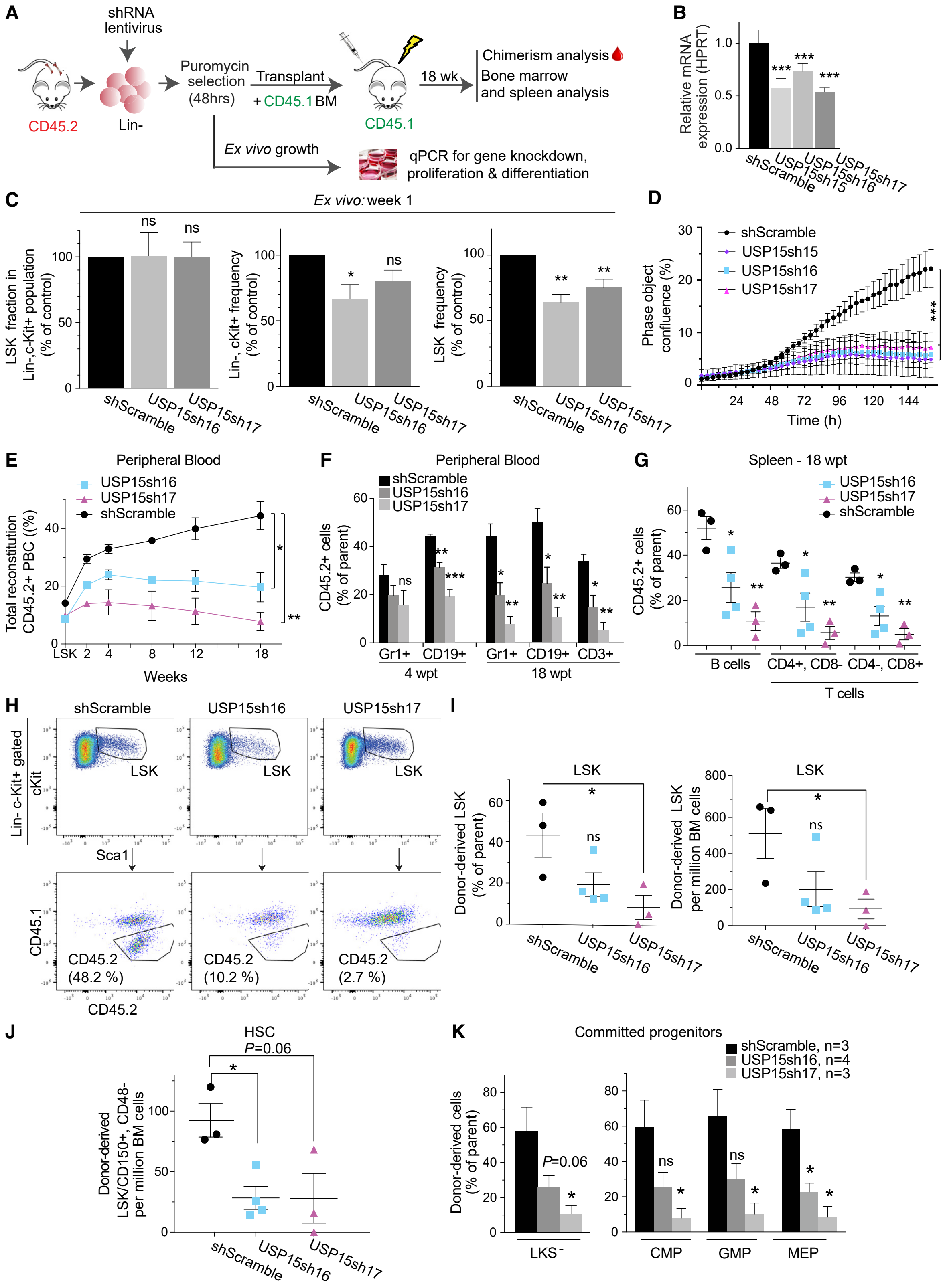

(legend on next page)

CellPress
OPEN ACCESS

maintenance, including USP1 (Parmar et al., 2010), USP3 (Lancini et al., 2014), and USP16 (Adorno et al., 2013; Gu et al., 2016), also scored top hits from the primary screen and were targeted by two independent shRNAs (Figures 1F and S2H; Table S3).

To validate our primary screen, we divided the primary library in two mostly nonoverlapping shRNA sub-pools (DUB1 and DUB2 sub-library) and performed secondary screens under similar transplantation conditions (Figures 1B, 1F, and S1C). In line with the primary screen, high hairpins representation *in vitro* and *in vivo* (>95%), low variance between individual mice, and the performance of positive control shRNAs support the overall good quality and reproducibility of the secondary screens (Figures 1F, 1G, and S2E–S2G; Table S3). Although many shRNAs showed similar changes in representation in the primary and in the secondary screens, a measurable variation was present, likely due to inconsistencies in transduction efficiency or to the stochastic gain or loss of shRNAs following *in vivo* growth (Table S3). To overcome this, we adopted stringent selection parameters. We considered as candidates those genes for which at least two shRNAs were depleted/enriched by 10-fold median in the BM relative to their representation in the T0 control (i.e., the injected cell population; adjusted p value $\leq$ 0.02) in each screen and that were called as hits in at least two independent experiments. When multiple hairpins showed opposite effect, the corresponding gene was excluded. By these criteria, our positive controls and 14 out of 81 DUB genes tested were validated in the secondary screens and defined as positive hits (Figures 1F and S2H).

To prioritize hits for follow-up, we focused on DUBs with reported high expression in LSK and in HSC (Cabezas-Wallscheid et al., 2014; Lancini et al., 2016). We focused on USP15, for which three independent shRNAs were depleted for >15-fold median in the BM after 4 weeks, and the top-scoring shRNAs showed a 60-fold dropout (Figures 1E and 1G; Table S3). USP15 (Baker et al., 1999) is expressed in the early progenitor compartment (LSK) and HSCs, as well as in blood and splenic B cells, and, among the depleted DUBs, it ranks as third in expression in LSK (Cabezas-Wallscheid et al., 2014; Lancini et al., 2016).

Together with our screen results, these data suggest a potential role for USP15 in hematopoiesis, though no functional study *in vivo* has yet been reported. We therefore decided to further investigate the role of USP15 in HSC biology.

USP15 Depletion Impairs HSPC Proliferation *In Vitro*

We first checked USP15 expression levels in normal hematopoiesis by surveying published gene expression datasets. In the mouse BM, *Usp15* expression is consistently high at the single-cell level, and expression is homogeneous in the entire hematopoietic tree, being expressed at similar level in single mouse long-term HSCs (LT-HSCs) and early lineage-committed progenitors (Figures S2I and S2J) (Nestorowa et al., 2016; Olsson et al., 2016). Importantly, *Usp15* expression pattern in the mouse is similarly conserved in humans, as inferred by *USP15* expression in $CD34^+$ human HSCs and early lineage-committed progenitors at the single-cell level (Figure S2K) (Pellin et al., 2019).

We addressed the impact of individual USP15-targeting shRNAs on hematopoietic progenitors *in vitro* and *in vivo* (Figure 2A). We first assessed the ability of the single shRNAs to reduce *Usp15* expression upon low MOI (<1). To cope with the paucity of Lin^- cells, we chose qRT-PCR as a readout. All three shRNAs identified in the secondary DUB screen (DUB2 sub-library; Figure 1G; Table S3) downregulated USP15 mRNA expression in freshly isolated, lentiviral-infected Lin^- cells (Figure 2B) and USP15 protein levels in primary murine lung cancer cells (Figure S3A). For functional validation, we prioritized the two top-scoring lentiviral shRNA vectors in the screen, and Lin^- cells were transduced with either a control (shScramble) or USP15-targeting #sh16 and #sh17 shRNAs. To determine the effect of USP15 depletion on the LSK compartment, the transduced cells were propagated in a serum-free medium supplemented with pro-self-renewal growth factors and analyzed by flow cytometry for the presence of LSK surface receptors at 1 week post-infection. Within the Lin^-, c-Kit^+ population, the fraction of LSKs remained comparable between USP15-depleted and control shRNA cells (Figure 2C, left panel). Nevertheless, the expansion of both Lin^-, c-Kit^+ and LSK cells was affected by USP15 depletion compared to control shRNA (Figure 2C, middle and right panels, and Figure S3B). Consistently, USP15 knockdown progenitors exhibited limited proliferation (Figure 2D).

Figure 2. USP15 Depletion Impairs HSPC Proliferation *In Vitro* and Reconstitution Potential *In Vivo*

(A) *In vitro* and *in vivo* validation assays for USP15-targeting shRNAs.

(B) Knockdown efficiency of shRNAs targeting USP15 in Lin^- cells as measured by qRT-PCR. Mean values of three technical replicates ± SD are shown.

(C) Flow cytometry analysis of Lin^- cells at 1 week post-infection. The frequency of LSKs in the Lin^-, c-Kit^+ population, as well as the frequency of Lin^-, c-Kit^+, and LSKs in the live culture, was calculated and normalized relative to shScramble control. n = 3 independent experiments. Mean values ± SEM are shown.

(D) Freshly purified Lin^- cells were plated 7 days post infection and monitored for growth. n = 4 wells per data point. Mean values ± SEM are shown.

(E–K). Freshly isolated WT Lin^- cells transduced with the indicated shRNAs were assayed in competitive BM transplantation. Mean values ± SEM are shown. n = 3 per shRNA, except for shUSP15#16 (n = 4).

(E and F) CD45.2 chimerism in peripheral blood (E) and contribution of transduced cells to myeloid ($Gr1^+$), B cell ($CD19^+$), and T cell ($CD3^+$) lineages in the blood (F) of recipients. PBC, peripheral blood cells.

(G) CD45.2 chimerism in B cell and T cell lineages in recipients' spleen at 18 wpt.

(H) Representative FACS profiles of the LSK compartment in recipients at 18 wpt.

(I) CD45.2 chimerism level in LSKs in primary recipients (left). Right: numbers of donor-derived LSKs in 10^6 viable BM cells at 18 wpt.

(J) Cell numbers of donor-derived HSCs (LSK/$CD150^+$/$CD48^-$) in 10^6 viable BM cells at 18 wpt.

(K) Fraction of donor-derived $LKSs^-$, CMPs, GMPs, and MEPs in primary recipients at 18 wpt.

*p $\leq$ 0.05; **p $\leq$ 0.01; ***p $\leq$ 0.001. p value was assessed by Student's t test or multiple t test (D) in Prism 7. See also Figure S3.

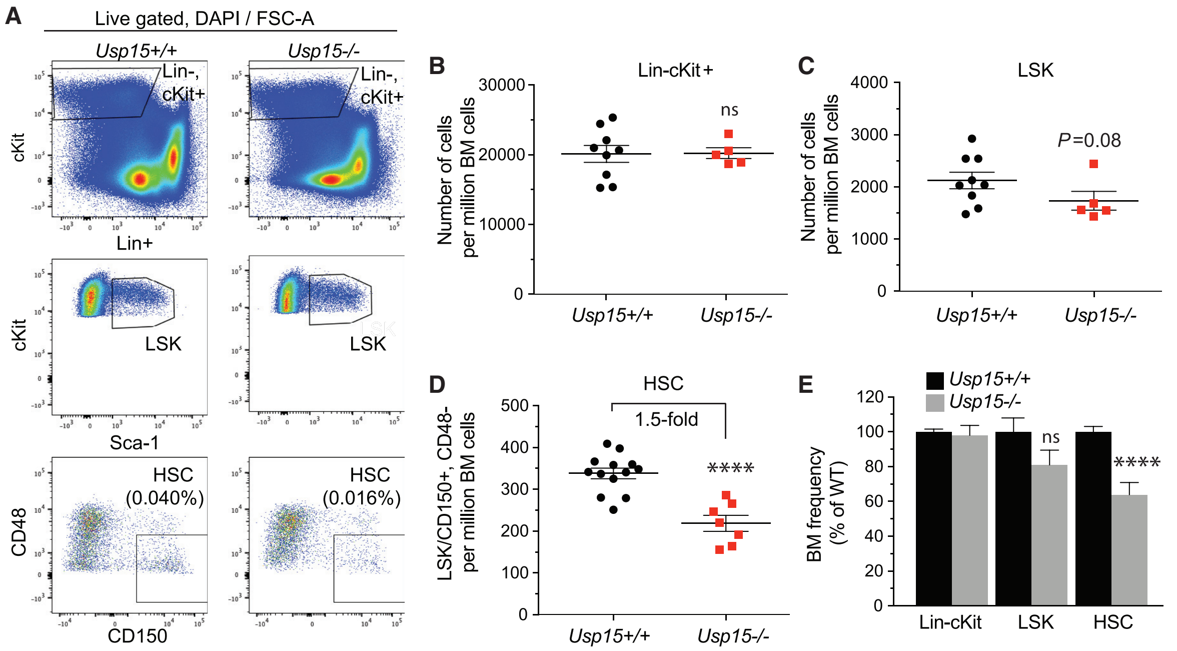

Figure 3. Reduced HSC Compartment in *Usp15* Knockout (KO) Mice

Flow cytometry analysis of the hematopoietic primitive populations in 8- to 12-week-old $Usp15^{+/+}$ and $Usp15^{-/-}$ mice.

(A) Representative FACS profiles of the Lin^-, $c\text{-}Kit^+$, LSK, and HSC populations. Frequency of HSCs in the live cell population is presented.

(B) Lin^-, $c\text{-}Kit^+$ cell numbers per million live BM cells.

(C) LSK cell numbers per million live BM cells.

(D) HSC (LSK, $CD150^+$, $CD48^{-/-}$) cell numbers per million live BM cells.

(E) Frequency of Lin^-, $c\text{-}Kit^+$, LSK and HSC in BM of $Usp15^{-/-}$ mice was calculated and normalized to $Usp15^{+/+}$ animals.

Results are from three (Lin^-, $c\text{-}Kit^+$, and LSK; $Usp15^{+/+}$ n = 9; $Usp15^{-/-}$, n = 5) or four (HSC; $Usp15^{+}/^{+}$, n = 13; $Usp15^{-/-}$, n = 7) independent experiments. *p $\leq$ 0.05, ****p $\leq$ 0.001; n.s., not significant. Error bars represent ± SEM. See also Figure S4.

USP15 Depletion Impairs Stem and Progenitor Cell Reconstitution Potential *In Vivo*

We then transduced murine Lin^- progenitors with USP15-targeting or control shRNAs and competitively co-transplanted these CD45.2 USP15-depleted or control progenitors together with freshly isolated CD45.1 BM cells (1:1 ratio) into lethally irradiated recipients. Within a period of 18 weeks, USP15 knockdown Lin^- cells failed to contribute to a chimerism level beyond the 20% of total peripheral blood cells, whereas the chimerism level of control mice progressively increased, reaching the expected ~50% contribution (Figure 2E). This underscores a competitive disadvantage of USP15-depleted cells compared to control cells. At 18 wpt, we found that all lineages within CD45.2 USP15-depleted peripheral blood cells, including myeloid/granulocytes ($CD11b^+$, $GR1^+$ cells), B cells, and T cells, were equally affected as compared to their control counterparts (Figures 2F and S3C). As observed in the blood, USP15 loss affected multilineage reconstitution (B cells and T cells) of recipient animals' spleen at 18 wpt, with an average 52% of control B cells compared to 25% and 10.8% of USP15-#sh16 and USP15-#sh17 cells, respectively (Figures 2G and S3D). As expected, the total cell numbers in the spleen and BM of euthanized recipient mice were comparable (Figure S3E).

The above results suggest a defect in the multilineage reconstitution potential of USP15-depleted progenitors. Given that BM-resident HSCs are mainly responsible for giving rise to and maintaining all blood cell lineages (Kiel et al., 2005; Naik et al., 2013; Wilson et al., 2008), we quantified the numbers of $CD45.2^+$ cells in the BM of recipients transplanted with either USP15-depleted or control progenitors at 18 wpt (Figures 2H–2K, S3F, and S3G). We then assessed stem cell reconstitution. In line with the overall lower relative contribution to the blood (Figure 2F), we measured a defect in USP15-depleted BM precursors. USP15-depleted LSKs were reduced in frequency and numbers (2.38- and 8-fold reduction, respectively) compared to control (shScramble) LSKs, which reached 50% contribution to the LSK compartment in recipient mice (Figures 2H, 2I, and S3F). To specifically focus on HSCs, we then employed the HSC surface receptors SLAM CD48 and CD150 markers (Cabezas-Wallscheid et al., 2014; Kiel et al., 2005; Oguro et al., 2013). We found a significant decline (3.25-fold) of CD42.2 HSCs (as defined by LSK/$CD48^-$/$CD150^+$) in the BM of animals reconstituted with USP15-depleted cells compared to controls (Figures 2J and S3F).

USP15 depletion resulted in a consistent decrease in donor-derived cells also in the more differentiated, proliferative LKS^- ($Lin^-Sca1^-c\text{-}Kit^+$) progenitors. A similar reduction of USP15-depleted cells compared to controls was measured in the myeloid subsets of common myeloid progenitors (CMPs) and granulocyte-monocyte progenitors (GMPs), as well as in the

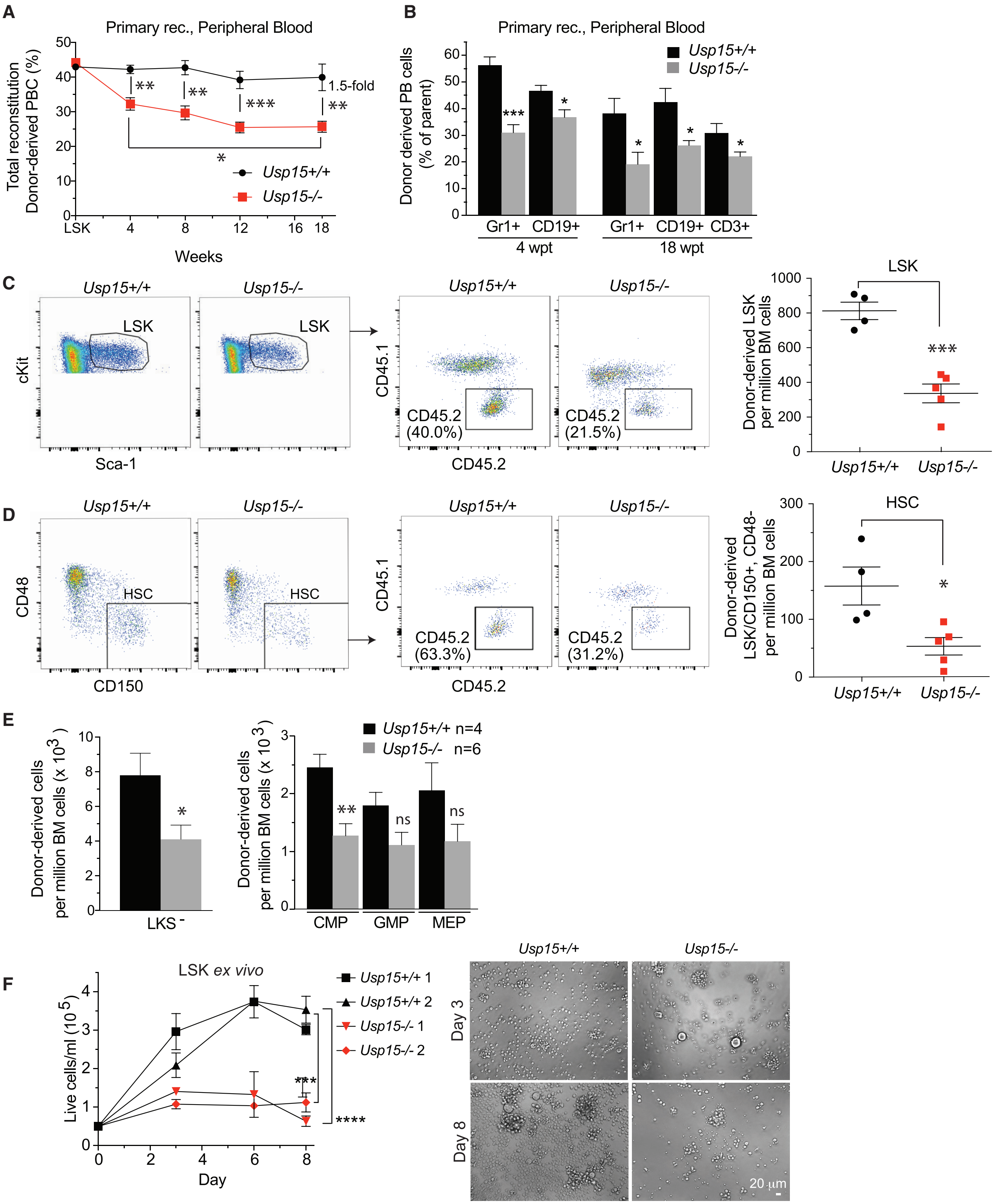

(legend on next page)

megakaryocyte-erythrocyte progenitors (MEPs) (Figures 2K and S3G) (Yeung and So, 2009), confirming an important role for USP15 in preserving all the main hematopoietic differentiation pathways.

USP15 Knockout (KO) Compromises Normal HSC Function *In Vivo*

To assess the role of USP15 in physiological hematopoiesis, we generated mice deficient for USP15 (Pritchard et al., 2017) (Figure S4A). Deletion of the *Usp15* locus was confirmed by PCR genotyping and western blot (Figures S4B and S4C). Homozygous *Usp15*$^{-/-}$ mice were viable, indicating that USP15 is dispensable for embryonic development. However, *Usp15*$^{-/-}$ animals were born at sub-Mendelian ratio and showed reduced survival and lower body weight when compared to *Usp15*$^{+/+}$ mice, confirming a critical role for USP15 *in vivo* (Figures S4D–S4F). Some of the *Usp15* KO animals showed evidence of inflammatory lesions (Figures S4G and S4H; Table S7).

We next screened young adult *Usp15*$^{+/+}$ and *Usp15*$^{-/-}$ littermates (8–14 weeks) for BM cellularity. No marked differences were found, suggesting that USP15-deficient BM can develop to a large extent normally (Figure S4I). In line with this, phenotypic analysis revealed a normal frequency in the Lin^{-}, c-Kit^{+} population in *Usp15*$^{-/-}$ and control mice (Figures 3A, 3B, 3E, and S4M), with a modest (but not significant) reduction in the *Usp15*$^{-/-}$ more undifferentiated stem and progenitors, the LSKs (Figures 3A, 3C, 3E, and S4M). Notably, within LSKs, the frequency and numbers of immature precursors endowed with reconstitution potential (LSK, CD135^{-}, CD150^{+}) (Christensen and Weissman, 2001; Kiel et al., 2005; Yeung and So, 2009) (Figures S4J–S4L) and, more specifically, phenotypic HSCs (LSK, CD48^{-}, CD150^{+}) (Cabezas-Wallscheid et al., 2014; Kiel et al., 2005; Oguro et al., 2013) were significantly lower in KO mice, reaching only 60% of their aged-matched wild-type (WT) controls (Figures 3A, 3D, 3E, and S4M). The more committed (myeloid) progenitor pools did not show any measurable phenotype (Figure S4N). Consistently, *Usp15*$^{-/-}$ BM cells performed similar to WT BM when assayed *in vitro* in myeloid colony-formation assays (colony-forming units in culture [CFU-Cs]) (Figure S4O).

To establish whether the HSCs remaining in *Usp15* KO mice are functionally equivalent to those in WT littermates, we performed competitive BM transplantations. Upon transplantation of BM cells containing a 1:1 mixture of test and competitor cells, chimerism of CD45.2 *Usp15*$^{-/-}$ peripheral blood cells in recipients significantly decreased over time compared to mice transplanted with *Usp15*$^{+/+}$ BM (Figure 4A). *Usp15*$^{+/+}$ chimerism remained constant throughout the 18 weeks of analysis and reached the expected plateau. Importantly, USP15 deletion critically affected myeloid/granulocytes (CD11b^{+}/Gr1^{+}) as well as lymphoid blood cells (CD19^{+} B cells and CD3^{+} T cells) (Figures 4B and S3C). This phenotype recapitulates the USP15 knockdown defects observed upon transplantation of shRNA-transduced Lin^{-} cells (Figures 2E and 2F). In recipient BM at 18 wpt, we found significantly lower numbers of *Usp15*$^{-/-}$ LSKs as well as HSCs (LSK, CD150^{+}, CD48^{-}) compared to WT controls, suggesting that USP15-deficient HSCs have reduced self-renewal capacity in recipients compared to WT HSCs (Figures 4C, 4D, and S5A). Consequently, the more committed *Usp15*$^{-/-}$ LKS^{-} and CMP pools were diminished (Figures 4E and S5B).

We next examined the consequences of USP15 deletion on HSPC cellular homeostasis. By DAPI/immuno-phenotyping combined analysis of freshly isolated BM cells, we measured that *Usp15*$^{-/-}$ mice have similar numbers of quiescent HSPCs compared to WT mice. The majority of HSCs were in the G0/G1 phase of the cell cycle. Under these physiological conditions, no subsets of HSPCs or HSCs differed significantly in terms of percentage of cells in S/G2 phase (Figure S5C). Of note, freshly isolated *Usp15*$^{-/-}$ stem and progenitor cells did not show apparent apoptosis (Figure S5D). Cleaved-caspase-3-positive cells were not readily detected on BM tissue sections of *Usp15*$^{-/-}$ mice (Figure S5E). RNA sequencing (RNA-seq) of WT and *Usp15*$^{-/-}$ LSKs confirmed the loss of *Usp15* and the maintenance of an overall stable identity of the cellular compartment (Figure S5F).

Having established a functional defect in *Usp15*$^{-/-}$ LSKs upon transplantation, we next assayed their intrinsic proliferative capacity in conditions of cytokine-induced replication. In *in vitro* liquid cultures, FACS-sorted *Usp15*$^{-/-}$ LSKs displayed a significantly reduced proliferative capacity compared to WT, which was exacerbated upon *ex vivo* culturing (Figure 4F).

USP15 Is Highly Expressed in Human Leukemia

LSCs share functional properties with normal HSCs. Acute myeloid leukemia (AML) and chronic myeloid leukemia (CML) arise in the early hematopoietic compartment and have LSCs endowed with self-renewal and ability to propagate the disease (Kreso and Dick, 2014; Warr et al., 2011).

Consistent with this, USP15 featured the highest of expression in human hematopoietic tissues and related cancers, including leukemia and lymphomas (The Cancer Genome Atlas [TCGA]) (Figures 5A and 5B). In an AML-specific dataset, *USP15* expression was significantly higher in patients with AML carrying various genetic abnormalities compared to the normal human CD34^{+}-enriched BM hematopoietic precursors (Figure 5C)

Figure 4. Genetic KO of *Usp15* Impairs HSC Function

(A–E) Competitive transplantation of BM cells freshly isolated from *Usp15*$^{+/+}$ or *Usp15*$^{-/-}$ mice.

(A and B) Chimerism in peripheral blood (A) and contribution of BM cells to myeloid (Gr1^{+}), B cell (CD19^{+}) and T cell (CD3^{+}) lineages in the blood (B) of recipients.

(C) Representative FACS profiles (left) and numbers of donor-derived LSKs per million viable BM cells in recipients at 18 wpt.

(D) Representative FACS profiles (left) and numbers of donor-derived HSCs (LSK/CD150^{+}/CD48^{-}) per million viable BM cells in recipients at 18 wpt.

(E) Numbers of donor-derived myeloid committed progenitor populations (LKSs^{-}, CMPs, GMPs, and MEPs) in recipients at 18 wpt.

(F) FACS-sorted LSKs were plated after 8 days (second plating) in culture and monitored for growth. n = 4 wells per data point. Representative images at days 3 and 8 of the second plating are shown. Bar, 20 μm.

*$p \leq 0.05$; **$p \leq 0.01$; ***$p \leq 0.001$. Error bars represent mean ± SEM (A–E); data represent one representative experiment out of three. See also Figure S5.

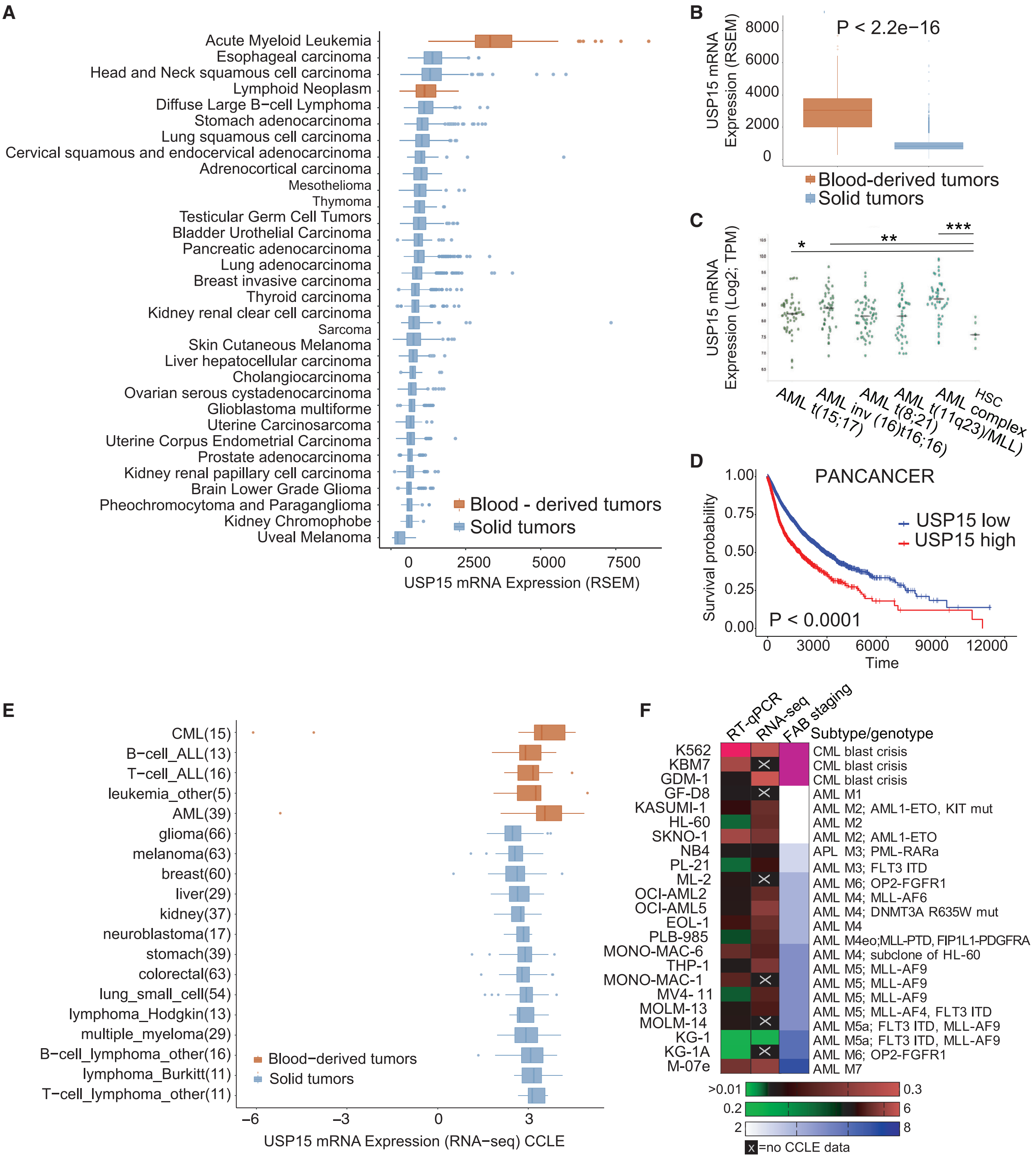

Figure 5. USP15 Is Highly Expressed in Primary Blood-Derived Cancer

(A) Cohort TCGA pan-cancer (PANCAN) (total number of samples, 11,060) shows upregulation of *USP15* in AML.

(B) Cohort TCGA PANCAN (11,060) shows upregulation of *USP15* in blood-derived tumors.

(C) Log2-transformed expression of *USP15* from microarray-based gene expression profiling of human BM cells (Hemaexplorer). TPM, transcripts per kilobase million.

(D) Kaplan-Meier curve correlating survival of 10,951 PANCAN patients with *USP15* gene expression. TCGA PANCAN samples used in this analysis are shown in Table S8.

(legend continued on next page)

(Bagger et al., 2013) (Hemaexplorer; http://servers.binf.ku.dk/bloodspot/). Of note, high expression of *USP15* is statistically associated with tissue-independent poor survival within the pan-cancer (PANCAN) patient cohort, a feature generally associated with oncogenes (Figure 5D; Table S8).

To test whether these data are reflected in human cancer models, we next analyzed *USP15* expression in the large panel of comprehensively characterized Cancer Cell Line Encyclopedia (CCLE). In line with the previous analyses, the highest expression was found in leukemia cell lines, including multiple AML and CML cell lines, compared to all other tissues (Figure 5E). To experimentally validate these analyses, we profiled USP15 expression in a panel of 23 leukemia cell lines, including all maturation stages and chemotherapy-resistant CML lines. With the sole exception of the KG1/KG1a cell line, USP15 mRNA was high in all the tested lines and independent of the leukemia stage. Interestingly, K562 and KBM7 blast crisis lines have very high *USP15* expression (Figure 5F).

To test whether *USP15* gene expression correlates with its genetic dependency, we ranked the dependency scores calculated by DEMETER2 (D2) for USP15 RNAi in CCLE lines (McFarland et al., 2018). According to DepMap (https://depmap.org/portal), USP15 expression and dependency varied across cell lines but were not linearly correlated, and leukemia cell lines were not specifically sensitive compared to other cancers (Figure S6A). Next, we investigated whether cancer-related biological pathway activation would be informative as a biomarker for USP15 dependency. To this end, we compiled a list of cell lines in which sensitivity to USP15 depletion was experimentally tested and could be classified as relatively high (<-0.2) or low (>0.2) by D2 score. Among the leukemia cell lines, MV-4-11 and Kasumi-1 featured highly sensitive and SEM and K562 featured as less sensitive cell lines (Figure S6A). Using PROGENy (Schubert et al., 2018), differential pathway activation between cell lines with varying degrees of sensitivity indicate that several RTK (receptor tyrosine kinase), JAK/STAT, and phosphatidylinositol 3-kinase (PI3K) signaling pathways tend to anticorrelate with sensitivity to USP15 depletion, whereas VEGFA, HIF1A, and transforming growth factor beta (TGF-β) signaling were found more active in highly sensitive cell lines (Figure S6B). Across the whole spectrum of CCLE cell lines, however, there was no evident biomarker for response, except a trend for activation of the Trail pathway (Figure S6C), suggesting that USP15 depletion may operate in context-dependent manner. To experimentally address the potential impact of the regulation of these pathways in response to USP15 depletion, we next performed RNAi of USP15 on highly expressing KBM7 and K562 CML cell lines. The K562 cell line is considered to have low sensitivity within the DepMap dataset, and therefore, response to USP15 RNAi may be uncoupled from survival. Ingenuity pathway analysis identified 657 and 330 differentially regulated genes in KBM7 and K562, respectively. In line with PROGENy analysis, RNAi of USP15 led to activation of inflammation-related pathways, which involve JAK/STAT and PI3K signal transduction (Figures S6D–S6G). In K562, we also measured significant down-modulation of TGF-β signaling (Figures S6H and S6I).

USP15 Loss Enhances Genotoxic Stress in Leukemia Cell Lines and Mice

Given the context-dependent responses to USP15 depletion in CML cells and that reversal of ubiquitination often contributes to fine-tuning of the DDR (Nishi et al., 2014), we next focused on exploring a potential role for USP15 in genome maintenance.

USP15 depletion by USP15-targeting small interfering RNAs (siRNAs) mildly but reproducibly reduced the viability of both "less sensitive" K562 and KBM7 and "more sensitive" MV411 and Kasumi-1 cell lines (Figures 6A, 6B, and S6A; see below). Despite the predicted low sensitivity to USP15 depletion, USP15 loss was accompanied by a significant increase in the number of spontaneous nuclear foci of the DDR factor 53BP1 as well as an increase in the basal levels of γ-H2AX, a DNA damage marker, and the frequency of micronuclei in both K562 and KBM7 cells (Figures 6C–6F), all indicative of enhanced genotoxic stress. This mirrors the increase in micronucleation, as well as bi- and multinucleation and apoptotic/necrotic cells observed in FACS-sorted LSKs from the BM of *Usp15*$^{-/-}$ mice upon culturing (Figure 6G) and their increase in spontaneous γ-H2AX nuclear foci (Figure 6H), thereby indicating that USP15 loss affects genome integrity in all of these settings. Spontaneous genotoxic stress was also observed in USP15 depleted osteosarcoma cells (Figures S7A–S7H), thereby extending the validity of USP15 expression as genome integrity safeguard mechanism to multiple tissue neoplasia.

These data supported the hypothesis that USP15 depletion would render normal HSPCs more sensitive to genotoxic stress *in vivo*. To test this, we injected mice with the chemotherapeutic agent cisplatin (Pilzecker et al., 2017) intravenously (i.v.), or with PBS, and analyzed the BM after 2 days. Upon cisplatin treatment, USP15 KO BM cells produced significantly fewer CFUs compared to WT (Figure S7I), suggesting higher sensitivity of their HSPC compartment. Deeper BM analysis unmasked a broader sensitivity of the primitive progenitor compartment in *Usp15*$^{-/-}$ mice, including HSCs and LSKs and the more proliferative LKS^{-}, myeloid (GMP), and lymphoid (CLP) progenitor populations, to genotoxic stress (Figures S7J and S7K).

Finally, we sought to translate these findings into a potential combination setting in leukemia. In leukemia cells originated by blast crisis such as KBM7 cells, we combined depletion of USP15 by doxycycline (dox)-inducible RNAi and DNA breaks induction by ionizing radiation (IR). USP15 depletion by a dox-inducible shRNA sensitized KBM7 cells to IR (Figure 6I). In keeping with a role of USP15 in DDR (Peng et al., 2019), Rad51 protein levels were diminished by USP15 knockdown in MV4-11 and Kasumi-1 leukemia cells (Figure S7L). A broader chemo-profiling in CCLE cancer cell lines indicated that leukemia cell lines are generally more sensitive than others to the DNA damage

(E) Expression of *USP15* from transcriptional profiling of human cancer cell lines (CCLE, Broad Institute).
(F) USP15 normalized expression levels in a panel of leukemia cell lines as assessed by qRT-PCR and RNA-seq by CCLE. The right column indicates the relative FAB (French-American-British) stage for leukemia subtype classification.
See also Figure S6.

CellPress
OPEN ACCESS

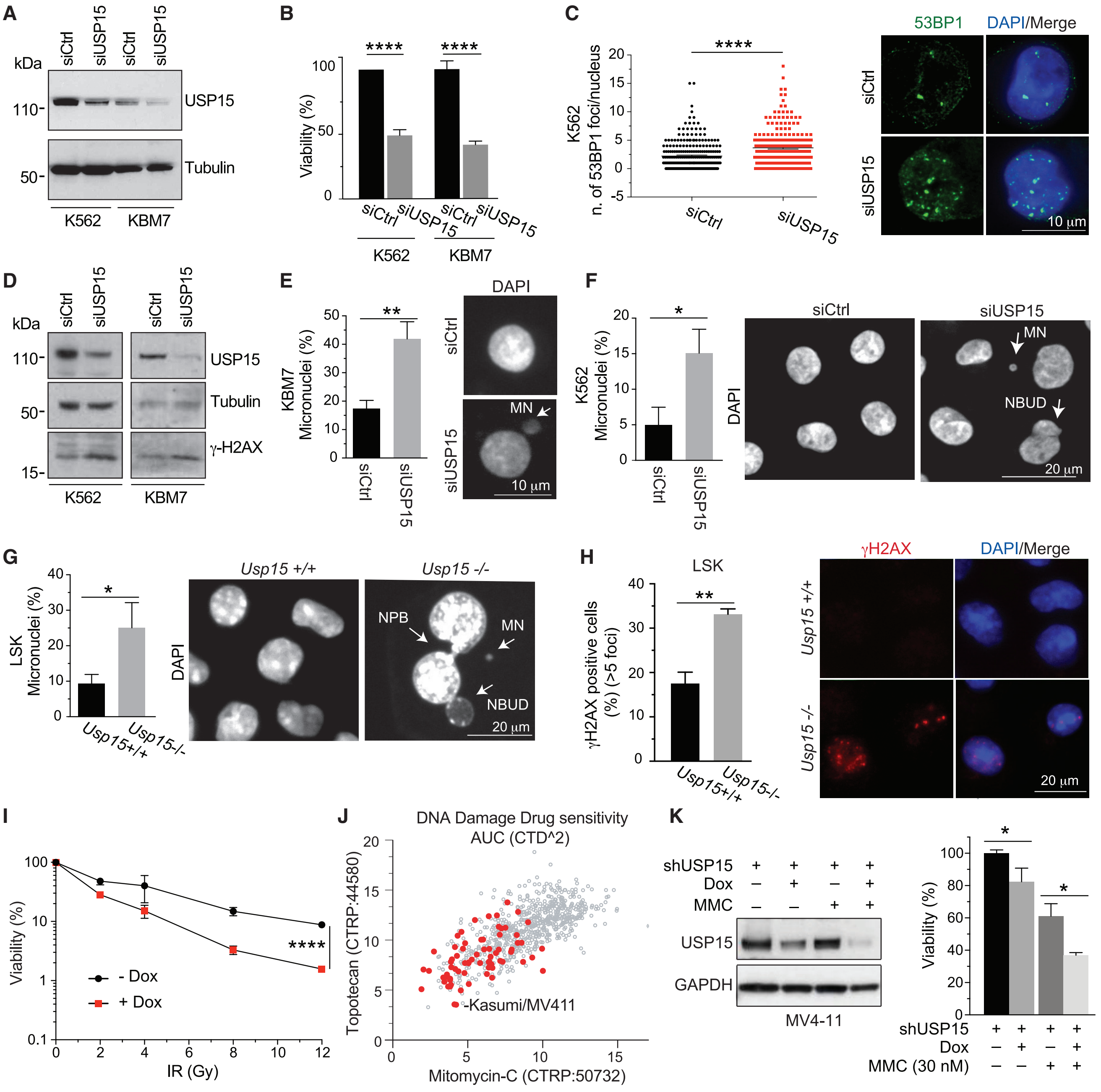

Figure 6. USP15 Loss Enhances Genotoxic Stress in Human CML Leukemic Cells and Mouse Normal Hematopoietic Progenitors

(A–F) K562 and KBM7 CML cell lines transfected with USP15 (siUSP15) or non-targeting (siCtrl) siRNAs and assayed at 72 h after transfection.

(A) Immunoblotting on whole-cell extracts.

(B) Cell viability. Mean ± SD from three independent experiments are shown.

(C) Representative images and quantification by ImageJ of the number of spontaneous 53BP1 foci/cell. Mean values ± SEM are shown. n = 2. A minimum of 250 cells per sample was counted over two independent experiments.

(D) Immunoblotting of USP15-depleted cell lines.

(E and F) Quantification and representative images of micronuclei (MN; arrows) in KBM7 (E) and K562 (F). Results are mean ± SD from three independent experiments. A minimum of 150 (KBM7) or 450 (K562) cells was scored.

(G) Percentage of MN in FACS sorted, murine LSK after 11 d in culture. A minimum of 60 cells/genotype was scored in two independent experiments (each experiment: *Usp15*$^{+/+}$, n = 3; *Usp15*$^{-/-}$, n = 2). Mean ± SD is shown.

(H) Immunofluorescence staining for γH2AX on LSK after 5d in culture. Percentage of cells containing >5 spontaneous γH2AX nuclear foci and representative images are shown. A minimum of 60 cells/genotype/sample was scored per experiment in two independent experiments (each experiment: *Usp15*$^{+}$/$^{+}$, n = 3; *Usp15*$^{-/-}$, n = 2). Mean ± SEM is shown.

(legend continued on next page)

inducers topotecan and mitomycin-C (MMC), two chemotherapeutic clastogenic agents (Figure 6J). Notably USP15-depletion cooperated with MMC to reduce cell viability in MV4-11 (Figure 6K).

USP15 Regulates FUS Stability in Leukemia Cells

To gain mechanistic insight into how USP15 contributes to preserve genome integrity, we next determined USP15 interactors in MV4-11 and Kasumi-1 cells, which are sensitive to acute USP15 depletion (Figure S6A). To isolate USP15 direct interactors, we immunoprecipitated endogenous USP15 in both naive and DNA stress conditions (MMC; Figures 7A and 7B). By mass spectrometry, we identified 355 candidates that co-immunoprecipitated with USP15 in all the conditions. Stringent filtering of high-confidence interactors (n = 4/condition, adjusted $p < 0.05$ against immunoglobulin G [IgG]) returned 38 USP15 interactors shared by MV4-11 and Kasumi-1 cell lines, including known interactors (e.g., USP4 and USP11; Figure 7C). Importantly, 33 (~87%) were not previously reported as USP15 interactors in BioGRID (Figure 7C). To focus on DDR-related processes, we used pathway analysis of the 38 candidates by Reactome. Consistent with a potential role for USP15 in DDR, we found that FUS, TAF15, USP11, USP4, and CHMP4B proteins are associated with DNA repair, and MCM5 is associated with DNA replication processes (Figure 7D). We focused on FUS, a bona fide USP15 interactor based on identity score, peptide number, and interaction intensity in both MV4-11 and Kasumi-1, including under DNA stress conditions (Figure 7D).

FUS is an RNA/DNA-binding protein that is reported to promote HSC self-renewal (Sugawara et al., 2010) and is highly expressed in leukemia cell lines (https://depmap.org/portal). FUS contributes to DNA repair by promoting DNA homologous pairing (Bertrand et al., 1999) and D-loop formation (Baechtold et al., 1999), as well as by facilitating DDR site loading with HDAC1 (Wang et al., 2013) and compartmentalization of damaged DNA (Singatulina et al., 2019). We validated the endogenous interaction between USP15 and FUS by direct and reverse co-immunoprecipitation in both MV4-11 and Kasumi-1 (Figure 7E-G). Given that USP15 can potentially regulate the stability of its interactors and FUS is exported from the nucleus to the cytoplasm after DNA repair (Singatulina et al., 2019), we investigated whether USP15 was altering FUS stability or location and in which cellular compartment. To this end, we generated MV4-11 USP15 KO cells by CRISPR-Cas9 KO, and we analyzed the nuclear and the cytoplasmic fractions by immunoblot. USP15 depletion reduced FUS levels in the cytoplasm, but not in the nucleus (Figure 7H). In line with previous reports, USP15 was mainly localized in the cytoplasm, whereas FUS was more nuclear (Urbé et al., 2012). Of note, FUS cytoplasmatic depletion in USP15 KO cells occurred without altering FUS nuclear levels (Figure 7H). Importantly, proteasome inhibition by low-dose bortezomib restored FUS levels in the cytoplasm of USP15 KO cells, supporting a role for USP15 in protecting FUS from proteasomal degradation (Figure 7I).

DISCUSSION

We report on the comprehensive assessment of the role for DUBs in early hematopoiesis through pooled *in vivo* shRNA screens in the mouse. Using this unbiased approach, we uncovered several genes within the family of DUBs whose loss increases or decreases mouse HSPC fitness *in vivo*. The top hit in our screens was USP15, which we herewith report as a DUB required for early hematopoietic progenitor proliferation and for HSC homeostasis *in vivo*. USP15 had a positive role in preserving normal stem and leukemic cell genome integrity and mediated the stability of a HSC self-renewal and DNA repair factor, FUS (fused in sarcoma).

Pooled *in vivo* screens in early progenitors pose specific technical challenges. The success of our shRNA screening approach is underscored by the maintenance of our shRNA library representation *in vitro* and *in vivo* and the ability to identify established regulators of HSC biology, including known DUBs. Together with the extensive genetic validation, these examples raise confidence in the identification of USP15 as critical regulator of HSCs *in vivo*.

Loss of USP15 in adult murine hematopoietic progenitors by RNAi or germline deletion impaired their growth *in vitro* and repopulation ability *in vivo*. Our data support the defective initial and long-term hematopoietic engraftment to contribute to USP15-deficient HSC loss during transplantation. HSC/HSPC cells under physiological conditions *in vivo* did not display measurable cell-cycle abnormalities, which is consistent with either a role for USP15 during active replication or with technical limitations in the sensitivity of the assay. Future studies to address the proliferative status/cell-cycle progression will require single-cell assays of purified primary USP15 deficient HSC *ex vivo* or intravital imaging.

Under homeostatic conditions, genetic deletion of USP15 specifically affected the HSC reservoir in adult mice, while the more differentiated progenitors were largely maintained. Of note, the functional defect we observed in BM transplantation upon USP15 knockdown is reasonably comparable to that observed in *Usp15* KO cells under competitive repopulation

(I) KBM7 cells transduced with a doxycycline (dox)-inducible shUSP15 were grown with or without dox for 5 days and seeded for IR treatment. Cell viability was measured 3 days after IR. Values represent mean ± SD of two independent experiments (each with n = 5 replicates/sample) (two-way ANOVA, ****$p < 0.0001$).
(J) Scatterplot of area under the dose-response curve (AUC) scores indicating sensitivity of individual cell lines to either topotecan or mitomycin-C (MMC). Red dots indicate leukemia cell lines. Data are generated by Cancer Target Discovery and Development (CTD2) Network and taken from the Cancer Therapeutics Response Portal (CTRP).
(K) MV4-11 cells harboring USP15 shRNA were kept in medium with or without dox for 5 days and plated with 30 nM MMC. Western blot and cell viability assays were performed at 72 h of MMC treatment. Results are the mean ± SEM of three (−MMC) or two (+MMC) independent experiments (each with n = 3 replicates/sample).
*$p \leq 0.05$; **$p \leq 0.01$; ****$p \leq 0.0001$. In (I), ****$p < 0.0001$ (assessed by two-way ANOVA). Arrows indicate MN, nucleoplasmatic bridges (NPBs), and the nucleoplasmatic bud (NBUD). See also Figure S7.

CellPress
OPEN ACCESS

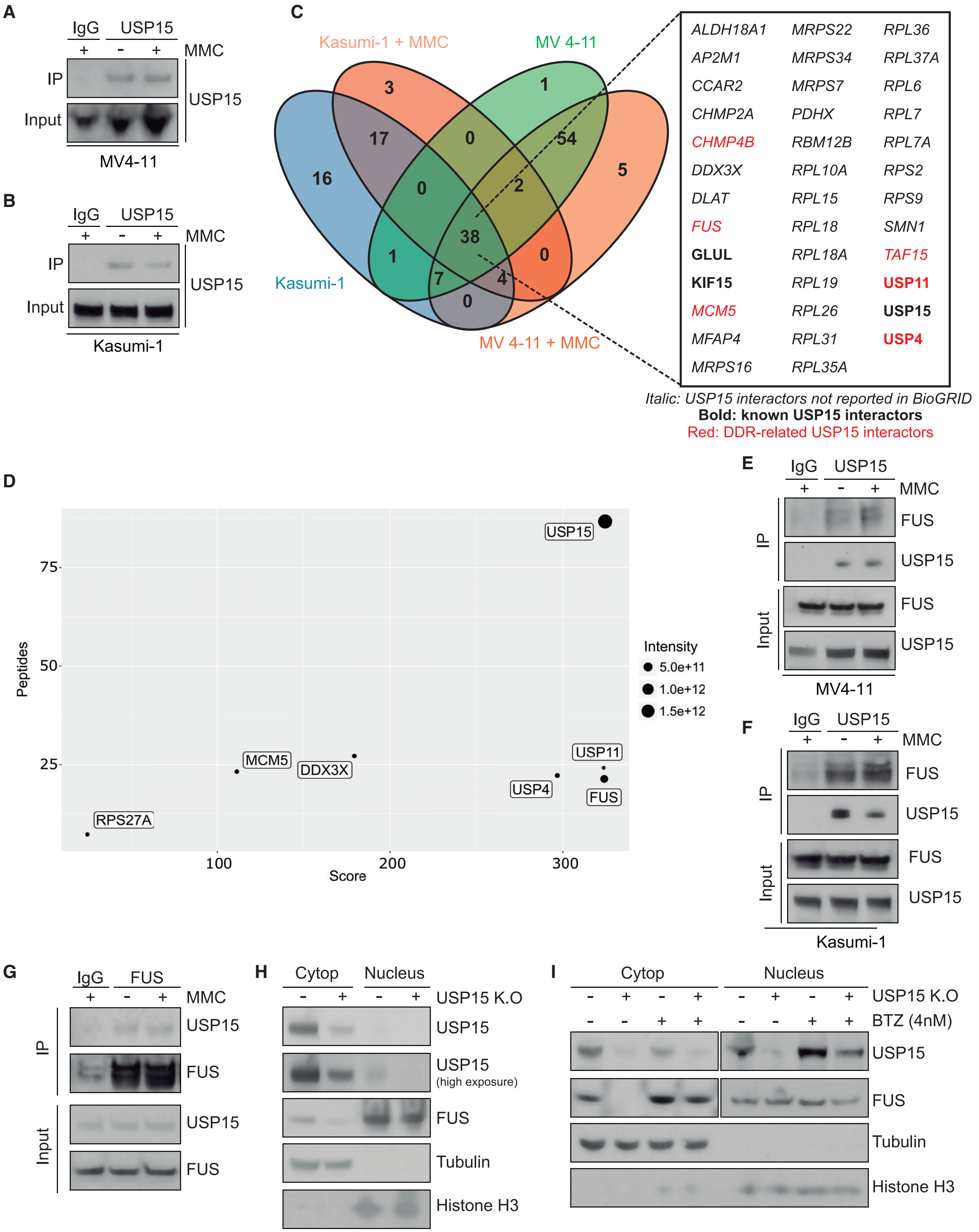

(legend on next page)

stress. We interpreted these data as the chronic lack of USP15 is compensated by protective pathways/adaptation to ensure hematopoiesis at steady state, whereas the acute loss of USP15 along with the repopulation stress unleashed a stronger phenotype. The net outcome is that USP15 is still required, but the extent of its requirement depends on the context (Chen et al., 2020). These data are consistent with a role for USP15 in contributing to homeostasis through the maintenance of HSCs, which are largely quiescent (Bakker and Passegué, 2013).

We report that spontaneous genotoxic stress and enhanced sensitivity to clastogenic agents accompanied the decrease in viability of USP15-deficient hematopoietic progenitors and leukemia cells *in vitro* and mouse primitive hematopoietic progenitors *in vivo*. These data link USP15 to the DDR and are consistent with previous work in cancer cell lines (Fielding et al., 2018; Mu et al., 2007; Nishi et al., 2014; Peng et al., 2019). Through *de novo* proteomics, we determined the USP15 interactome in leukemia cells, directly linking USP15 to the regulation of known DDR factors. In particular, USP15 stabilizes FUS, identified and validated as a functional USP15 interactor. While FUS's contribution to DNA repair is ultimately expected to take place in the nucleus (Singatulina et al., 2019; Wang et al., 2013), we observed that USP15 loss selectively affects cytoplasmic FUS. Physiological FUS function depends on proper shuttling between the nucleus and the cytoplasm (Naumann et al., 2018). Though several mechanisms may mediate FUS nucleo-cytoplasmatic shuttling (Deng et al., 2014; Kaneb et al., 2012; Monahan et al., 2017; Singatulina et al., 2019), its significance remains to be clarified (Rhoads et al., 2018). The interaction between USP15 and FUS resulted in lowering FUS cytoplasmic concentration, which may either affect protein function or more simply reduce the overall amount of protein available for nuclear shuttling. Of note, immunoprecipitated FUS was detected as two protein bands. This is in line with FUS being regulated by several post-translational modifications (Rhoads et al., 2018). Identifying these modifications may indicate the activation by specific pathways and help to elucidate the molecular mechanism linking FUS activity to USP15 in DDR.

Whereas USP15 is known to interact with MDM2 (Zou et al., 2014), in our experimental settings, we did not find evidence of USP15 phenotypes being dependent on the p53 pathway, and endogenous USP15 did not interact with MDM2 in our stringent proteomic analysis. Together, the data suggest that USP15 may support HSC self-renewal by contributing to swift DNA repair, which is in line with HSC relying on fine-tuning of DDR (Bakker and Passegué, 2013).

A functional role for USP15 in various cancers was previously described (Eichhorn et al., 2012; Fielding et al., 2018; Padmanabhan et al., 2018; Peng et al., 2019). Here, we provide functional ground for investigating the role for USP15 as gatekeeper in leukemia. The functional interaction between USP15 and FUS in blood cancer cells suggests that USP15 regulates DDR pathways in context-dependent manner. Hence, the role for USP15 in cell homeostasis is mechanistically broader than previously anticipated. Understanding how USP15 loss precisely impacts HSC and cancer cell maintenance and modulates their damage response may help to identify combinatorial treatment that affect leukemia self-renewal while sparing normal HSC from the side effects of conventional chemotherapy.

USP15 is involved in multiple cellular processes, including p53 (Liu et al., 2017; Niederkorn et al., 2020; Zou et al., 2014) and nuclear factor κB (NF-κB) (Schweitzer et al., 2007) signaling. USP15 regulates inflammation in experimental models (Torre et al., 2017; Zou et al., 2015) and promotes glioblastoma cell proliferation through stabilizing TGF-β signaling (Eichhorn et al., 2012). Although the regulation of inflammatory signals and TGF-β are relevant in both normal HSC and malignant development (Blank and Karlsson, 2015), the limited changes in gene expression detected in *Usp15*$^{-/-}$ LSKs suggest that USP15's function in preserving genome integrity is dominant in this compartment. However, our data raise the therapeutically interesting opportunity to investigate whether the role for USP15 in preserving self-renewal through genome integrity contributes to its functions in glioblastoma.

The function of USP15 in development is still poorly characterized. In addition to requirement for USP15 in HSC maintenance, our KO mice had impaired Mendelian transmission and lower lifespan. This phenotype is not obvious when compared with reports in a USP15 gene-trap model (Zou et al., 2014) but is in line with recent findings (Peng et al., 2019). Our data warrant further investigation of the role of USP15 at the organismal level.

In summary, we employed an unbiased approach to sensitively and selectively screen for DUB function in hematopoietic progenitors *in vivo*, through which we identified several DUB candidates. Major investments in DUB drug discovery have been made in the last 5–10 years, and more than 40 small molecules against DUBs have already been developed (Harrigan et al., 2018; Heideker and Wertz, 2015). Our data argue in favor of developing specific USP15 inhibitors, which are only starting to emerge (Teyra et al., 2019).

Figure 7. USP15 Interacts with FUS and Promotes Its Stabilization in Leukemia Cells

(A–D) USP15 interactome in MV4-11 and Kasumi-1 cell lines.

(A and B) Validation of endogenous USP15 immunoprecipitation previous to mass spectrometry analysis in MV4-11 cells (A) and Kasumi-1 cells (B) treated with 30 nM MMC for 1 h.

(C) Venn diagram of USP15 interactors for each condition (adjusted p value $\leq$ 0.05 compared to IgG; n = 4) showing the 38 common interactors. Italic text shows USP15 interactors not previously reported in the BioGRID database. Bold text shows known USP15 interactors according to the BioGRID database. Red indicates DDR-related proteins as per Gene Ontology categories based on the Reactome database.

(D) Plot showing the parameters of identification for the indicated USP15 interactors, defined by the number of peptides, score for the identification and intensity.

(E and F) Endogenous immunoprecipitation of USP15 in untreated or MMC-treated MV4-11 cells (E) and Kasumi-1 (F) cells followed by FUS detection.

(G) Reverse immunoprecipitation of endogenous FUS from MV4-11 cells co-immunoprecipitates endogenous USP15 with and without MMC treatment.

(H) Immunoblot of cytoplasmic and nuclear fractions in MV4-11 and MV4-11 USP15 KO cells.

(I) Immunoblot of USP15 WT and USP15 KO MV4-11 cells in the cytoplasmic and nuclear fractions after treatment with 4 nM bortezomib (BTZ) for 24 h. Cropped blots for FUS and USP15 correspond to different exposures.

USP15 is, together with USP4 and USP11, part of a closely related family of USPs (Nishi et al., 2014; Vlasschaert et al., 2015; Wijnhoven et al., 2015). They are all expressed in hematopoietic early progenitors (Cabezas-Wallscheid et al., 2014; Lancini et al., 2016), but only USP15 was linked to HSC activity (Niederkorn et al., 2020). All three genes scored as hits in our genetic screen and where found in complex in leukemia cells, suggesting that they may cooperatively contribute to HSC homeostasis. The potential biochemical interaction between USP15 and USP11 and their specific and redundant roles in a physiological setting support the rational design of allosteric degraders, which would have a stronger impact than individually targeted small molecules. More broadly, our study calls for a more systematic effort in understanding how DUBs regulate normal and malignant HSC biology as a critical route toward the selection of effective drug targets and targeted treatment combinations.

STAR★METHODS

Detailed methods are provided in the online version of this paper and include the following:

- KEY RESOURCES TABLE
- RESOURCE AVAILABILITY
  - Lead Contact
  - Materials Availability
  - Data and Code Availability
- EXPERIMENTAL MODELS AND SUBJECT DETAILS
  - Mice: generation and breeding of USP15 knockout mice
  - Leukemia cells
  - KPE cell line
  - U2OS cell line
- METHOD DETAILS
  - shRNA libraries
  - *In vivo* shRNA screens
  - PCR and next-generation sequencing
  - Bone marrow transplantation assays
  - Flow cytometry
  - Analyses and cell sorting of hematopoietic precursors
  - Cell cycle analysis of BM populations
  - Assessing Cleaved Caspase-3 levels of BM populations
  - Hematopoietic stem and progenitor cells liquid culture, time-lapse imaging and proliferation assays
  - CFU-C colony-forming assay
  - Leukemia cells RNA interference and cell viability assays
  - TCGA pan-cancer gene expression analysis and Single Cell Expression analysis
  - RNA-seq gene expression analysis
  - Sensitivity assays
  - Immunofluorescence and quantitative image analysis
  - Protein analysis
  - Quantitative real-time (qRT) PCR
  - Antibodies
  - U2OS cells genome editing: sgRNA design and cloning
  - Transfection and fluorescence activated cell sorting (FACS)
  - MV4-11 cells genome editing and electroporation
  - Genomic DNA extraction, PCR and Sanger sequencing and TIDE analysis
  - Sample preparation for mass spectrometry
  - Data analysis
  - Immunoprecipitation
  - Cellular fractionation
- QUANTIFICATION AND STATISTICAL ANALYSIS

SUPPLEMENTAL INFORMATION

Supplemental Information can be found online at https://doi.org/10.1016/j.celrep.2020.108533.

ACKNOWLEDGMENTS

We thank T. Sixma for critical reading of the manuscript; R. Kerkhoven, I. de Rink, and W. Brugman from the NKI Genomic Core Facility for deep sequencing; A. Pfauth, F. van Diepen, and M. van Baalen for assistance in flow cytometry; G. Chandrasekaran for data visualization; and F. Alemdehy, the MCCA transgenic facility, M. Massaro, B. van den Broek, the NKI Bio-Imaging Facility, the NKI Intervention Unit, the animal facility, and the Animal Pathology Department for assistance. Drug sensitivity data were taken from the CTD2 Network (https://ocg.cancer.gov/programs/ctd2/data-portal) established by the National Cancer Institute's Office of Cancer Genomics. This study was supported by the Dutch Cancer Society (KWF) grant to E.C. and M.v.L. (NKI-2012-5665 grant) and the ONCODE Institute to M.v.L. The G.G. lab acknowledges funding from the Max-Delbrück-Center for Molecular Medicine (MDC), the Helmholtz Association (VH-NG-1153) - Germany, and the European Research Council - ERC (714922).

AUTHOR CONTRIBUTIONS

E.C. and G.G. contributed to conceptualization and validation; E.C., P.v.d.B., C.L., C.C., D.H., M.S., J.Y.S., M.J.S., G.G., P.S., O.P., and P.M. contributed to investigation and methodology, helping with design and analysis of mass-spectrometry-based proteomics pull-down experiments; P.v.d.B., C.C., M.S., C.P., J.Y.S., I.J.H., and H.J. provided resources; P.v.d.B., C.L., C.C., G.G., and E.C. contributed to formal analysis; C.C. and G.G. provided software; E.C. wrote the original manuscript; G.G., P.v.d.B., H.J., and M.S. revised and edited the manuscript; E.C., G.G., and M.v.L. provided supervision and funding acquisition; and all authors read and commented on the manuscript and were involved during the editing of the manuscript.

DECLARATION OF INTERESTS

The authors declare no competing financial interests.

Received: January 29, 2020
Revised: July 28, 2020
Accepted: November 25, 2020
Published: December 29, 2020

REFERENCES

Adorno, M., Sikandar, S., Mitra, S.S., Kuo, A., Nicolis Di Robilant, B., Haro-Acosta, V., Ouadah, Y., Quarta, M., Rodriguez, J., Qian, D., et al. (2013). Usp16 contributes to somatic stem-cell defects in Down's syndrome. Nature *501*, 380–384.

Baechtold, H., Kuroda, M., Sok, J., Ron, D., Lopez, B.S., and Akhmedov, A.T. (1999). Human 75-kDa DNA-pairing protein is identical to the pro-oncoprotein TLS/FUS and is able to promote D-loop formation. J. Biol. Chem. *274*, 34337–34342.

Bagger, F.O., Rapin, N., Theilgaard-Mönch, K., Kaczkowski, B., Thoren, L.A., Jendholm, J., Winther, O., and Porse, B.T. (2013). HemaExplorer: a database of mRNA expression profiles in normal and malignant haematopoiesis. Nucleic Acids Res. *41*, D1034–D1039.

Baker, R.T., Wang, X.W., Woollatt, E., White, J.A., and Sutherland, G.R. (1999). Identification, functional characterization, and chromosomal localization of USP15, a novel human ubiquitin-specific protease related to the UNP oncoprotein, and a systematic nomenclature for human ubiquitin-specific proteases. Genomics *59*, 264–274.

Bakker, S.T., and Passegué, E. (2013). Resilient and resourceful: genome maintenance strategies in hematopoietic stem cells. Exp. Hematol. *41*, 915–923.

Bertrand, P., Akhmedov, A.T., Delacote, F., Durrbach, A., and Lopez, B.S. (1999). Human POMp75 is identified as the pro-oncoprotein TLS/FUS: both POMp75 and POMp100 DNA homologous pairing activities are associated to cell proliferation. Oncogene *18*, 4515–4521.

Biechonski, S., Yassin, M., and Milyavsky, M. (2017). DNA-damage response in hematopoietic stem cells: an evolutionary trade-off between blood regeneration and leukemia suppression. Carcinogenesis *38*, 367–377.

Blank, U., and Karlsson, S. (2015). TGF-β signaling in the control of hematopoietic stem cells. Blood *125*, 3542–3550.

Blomen, V.A., Májek, P., Jae, L.T., Bigenzahn, J.W., Nieuwenhuis, J., Staring, J., Sacco, R., van Diemen, F.R., Olk, N., Stukalov, A., et al. (2015). Gene essentiality and synthetic lethality in haploid human cells. Science *350*, 1092–1096.

Cabezas-Wallscheid, N., Klimmeck, D., Hansson, J., Lipka, D.B., Reyes, A., Wang, Q., Weichenhan, D., Lier, A., von Paleske, L., Renders, S., et al. (2014). Identification of regulatory networks in HSCs and their immediate progeny via integrated proteome, transcriptome, and DNA methylome analysis. Cell Stem Cell *15*, 507–522.

Cellot, S., Hope, K.J., Chagraoui, J., Sauvageau, M., Deneault, É., MacRae, T., Mayotte, N., Wilhelm, B.T., Landry, J.R., Ting, S.B., et al. (2013). RNAi screen identifies Jarid1b as a major regulator of mouse HSC activity. Blood *122*, 1545–1555.

Cerami, E., Gao, J., Dogrusoz, U., Gross, B.E., Sumer, S.O., Aksoy, B.A., Jacobsen, A., Byrne, C.J., Heuer, M.L., Larsson, E., et al. (2012). The cBio cancer genomics portal: an open platform for exploring multidimensional cancer genomics data. Cancer Discov. *2*, 401–404.

Chen, L.L., Smith, M.D., Lv, L., Nakagawa, T., Li, Z., Sun, S.C., Brown, N.G., Xiong, Y., and Xu, Y.P. (2020). USP15 suppresses tumor immunity via deubiquitylation and inactivation of TET2. Sci. Adv. *6*, 6.

Christensen, J.L., and Weissman, I.L. (2001). Flk-2 is a marker in hematopoietic stem cell differentiation: a simple method to isolate long-term stem cells. Proc. Natl. Acad. Sci. USA *98*, 14541–14546.

Citterio, E. (2015). Fine-tuning the ubiquitin code at DNA double-strand breaks: deubiquitinating enzymes at work. Front. Genet. *6*, 282.

Cox, J., and Mann, M. (2008). MaxQuant enables high peptide identification rates, individualized p.p.b.-range mass accuracies and proteome-wide protein quantification. Nat. Biotechnol. *26*, 1367–1372.

de Haan, G., and Lazare, S.S. (2018). Aging of hematopoietic stem cells. Blood *131*, 479–487.

Deng, Q., Holler, C.J., Taylor, G., Hudson, K.F., Watkins, W., Gearing, M., Ito, D., Murray, M.E., Dickson, D.W., Seyfried, N.T., and Kukar, T. (2014). FUS is phosphorylated by DNA-PK and accumulates in the cytoplasm after DNA damage. J. Neurosci. *34*, 7802–7813.

Dey, A., Seshasayee, D., Noubade, R., French, D.M., Liu, J., Chaurushiya, M.S., Kirkpatrick, D.S., Pham, V.C., Lill, J.R., Bakalarski, C.E., et al. (2012). Loss of the tumor suppressor BAP1 causes myeloid transformation. Science *337*, 1541–1546.

Doench, J.G., Fusi, N., Sullender, M., Hegde, M., Vaimberg, E.W., Donovan, K.F., Smith, I., Tothova, Z., Wilen, C., Orchard, R., et al. (2016). Optimized sgRNA design to maximize activity and minimize off-target effects of CRISPR-Cas9. Nat. Biotechnol. *34*, 184–191.

Edgar, R., Domrachev, M., and Lash, A.E. (2002). Gene Expression Omnibus: NCBI gene expression and hybridization array data repository. Nucleic Acids Res. *30*, 207–210.

Eichhorn, P.J., Rodón, L., Gonzàlez-Juncà, A., Dirac, A., Gili, M., Martínez-Sáez, E., Aura, C., Barba, I., Peg, V., Prat, A., et al. (2012). USP15 stabilizes TGF-β receptor I and promotes oncogenesis through the activation of TGF-β signaling in glioblastoma. Nat. Med. *18*, 429–435.

Fabregat, A., Sidiropoulos, K., Viteri, G., Forner, O., Marin-Garcia, P., Arnau, V., D'Eustachio, P., Stein, L., and Hermjakob, H. (2017). Reactome pathway analysis: a high-performance in-memory approach. BMC Bioinformatics *18*, 142.

Fielding, A.B., Concannon, M., Darling, S., Rusilowicz-Jones, E.V., Sacco, J.J., Prior, I.A., Clague, M.J., Urbé, S., and Coulson, J.M. (2018). The deubiquitylase USP15 regulates topoisomerase II alpha to maintain genome integrity. Oncogene *37*, 2326–2342.

Flach, J., Bakker, S.T., Mohrin, M., Conroy, P.C., Pietras, E.M., Reynaud, D., Alvarez, S., Diolaiti, M.E., Ugarte, F., Forsberg, E.C., et al. (2014). Replication stress is a potent driver of functional decline in ageing haematopoietic stem cells. Nature *512*, 198–202.

Galeev, R., Baudet, A., Kumar, P., Rundberg Nilsson, A., Nilsson, B., Soneji, S., Törngren, T., Borg, Å., Kvist, A., and Larsson, J. (2016). Genome-wide RNAi screen identifies cohesin genes as modifiers of renewal and differentiation in human HSCs. Cell Rep. *14*, 2988–3000.

Gargiulo, G., Serresi, M., Cesaroni, M., Hulsman, D., and van Lohuizen, M. (2014). In vivo shRNA screens in solid tumors. Nat. Protoc. *9*, 2880–2902.

Gu, Y., Jones, A.E., Yang, W., Liu, S., Dai, Q., Liu, Y., Swindle, C.S., Zhou, D., Zhang, Z., Ryan, T.M., et al. (2016). The histone H2A deubiquitinase Usp16 regulates hematopoiesis and hematopoietic stem cell function. Proc. Natl. Acad. Sci. USA *113*, E51–E60.

Haahr, P., Borgermann, N., Guo, X., Typas, D., Achuthankutty, D., Hoffmann, S., Shearer, R., Sixma, T.K., and Mailand, N. (2018). ZUFSP deubiquitylates K63-linked polyubiquitin chains to promote genome stability. Mol. Cell *70*, 165–174.e166.

Harrigan, J.A., Jacq, X., Martin, N.M., and Jackson, S.P. (2018). Deubiquitylating enzymes and drug discovery: emerging opportunities. Nat. Rev. Drug Discov. *17*, 57–78.

Heideker, J., and Wertz, I.E. (2015). DUBs, the regulation of cell identity and disease. Biochem. J. *465*, 1–26.

Hubner, N.C., Bird, A.W., Cox, J., Splettstoesser, B., Bandilla, P., Poser, I., Hyman, A., and Mann, M. (2010). Quantitative proteomics combined with BAC TransgeneOmics reveals in vivo protein interactions. J. Cell Biol. *189*, 739–754.

Kaneb, H.M., Dion, P.A., and Rouleau, G.A. (2012). The FUS about arginine methylation in ALS and FTLD. EMBO J. *31*, 4249–4251.

Kiel, M.J., Yilmaz, O.H., Iwashita, T., Yilmaz, O.H., Terhorst, C., and Morrison, S.J. (2005). SLAM family receptors distinguish hematopoietic stem and progenitor cells and reveal endothelial niches for stem cells. Cell *121*, 1109–1121.

Kreso, A., and Dick, J.E. (2014). Evolution of the cancer stem cell model. Cell Stem Cell *14*, 275–291.

Kwasna, D., Abdul Rehman, S.A., Natarajan, J., Matthews, S., Madden, R., De Cesare, V., Weidlich, S., Virdee, S., Ahel, I., Gibbs-Seymour, I., et al. (2018). Discovery and characterization of ZUFSP/ZUP1, a distinct deubiquitinase class important for genome stability. Mol. Cell *70*, 150–164.e156.

Lancini, C., van den Berk, P.C., Vissers, J.H., Gargiulo, G., Song, J.Y., Hulsman, D., Serresi, M., Tanger, E., Blom, M., Vens, C., et al. (2014). Tight regulation of ubiquitin-mediated DNA damage response by USP3 preserves the functional integrity of hematopoietic stem cells. J. Exp. Med. *211*, 1759–1777.

Lancini, C., Gargiulo, G., van den Berk, P.C., and Citterio, E. (2016). Quantitative analysis by next generation sequencing of hematopoietic stem and progenitor cells (LSK) and of splenic B cells transcriptomes from wild-type and Usp3-knockout mice. Data Brief *6*, 556–561.

Laurenti, E., and Göttgens, B. (2018). From haematopoietic stem cells to complex differentiation landscapes. Nature *553*, 418–426.

CellPress
OPEN ACCESS

Liu, W.T., Huang, K.Y., Lu, M.C., Huang, H.L., Chen, C.Y., Cheng, Y.L., Yu, H.C., Liu, S.Q., Lai, N.S., and Huang, H.B. (2017). TGF-β upregulates the translation of USP15 via the PI3K/AKT pathway to promote p53 stability. Oncogene *36*, 2715–2723.

Lukas, J., Lukas, C., and Bartek, J. (2011). More than just a focus: the chromatin response to DNA damage and its role in genome integrity maintenance. Nat. Cell Biol. *13*, 1161–1169.

McFarland, J.M., Ho, Z.V., Kugener, G., Dempster, J.M., Montgomery, P.G., Bryan, J.G., Krill-Burger, J.M., Green, T.M., Vazquez, F., Boehm, J.S., et al. (2018). Improved estimation of cancer dependencies from large-scale RNAi screens using model-based normalization and data integration. Nat. Commun. *9*, 4610.

Mevissen, T.E.T., and Komander, D. (2017). Mechanisms of deubiquitinase specificity and regulation. Annu. Rev. Biochem. *86*, 159–192.

Miller, P.G., Al-Shahrour, F., Hartwell, K.A., Chu, L.P., Järås, M., Puram, R.V., Puissant, A., Callahan, K.P., Ashton, J., McConkey, M.E., et al. (2013). In Vivo RNAi screening identifies a leukemia-specific dependence on integrin beta 3 signaling. Cancer Cell *24*, 45–58.

Monahan, Z., Ryan, V.H., Janke, A.M., Burke, K.A., Rhoads, S.N., Zerze, G.H., O'Meally, R., Dignon, G.L., Conicella, A.E., Zheng, W., et al. (2017). Phosphorylation of the FUS low-complexity domain disrupts phase separation, aggregation, and toxicity. EMBO J. *36*, 2951–2967.

Morrison, S.J., and Spradling, A.C. (2008). Stem cells and niches: mechanisms that promote stem cell maintenance throughout life. Cell *132*, 598–611.

Mu, J.J., Wang, Y., Luo, H., Leng, M., Zhang, J., Yang, T., Besusso, D., Jung, S.Y., and Qin, J. (2007). A proteomic analysis of ataxia telangiectasia-mutated (ATM)/ATM-Rad3-related (ATR) substrates identifies the ubiquitin-proteasome system as a regulator for DNA damage checkpoints. J. Biol. Chem. *282*, 17330–17334.

Naik, S.H., Perié, L., Swart, E., Gerlach, C., van Rooij, N., de Boer, R.J., and Schumacher, T.N. (2013). Diverse and heritable lineage imprinting of early haematopoietic progenitors. Nature *496*, 229–232.

Naumann, M., Pal, A., Goswami, A., Lojewski, X., Japtok, J., Vehlow, A., Naujock, M., Günther, R., Jin, M., Stanslowsky, N., et al. (2018). Impaired DNA damage response signaling by FUS-NLS mutations leads to neurodegeneration and FUS aggregate formation. Nat. Commun. *9*, 335.

Navarro, S., Meza, N.W., Quintana-Bustamante, O., Casado, J.A., Jacome, A., McAllister, K., Puerto, S., Surrallés, J., Segovia, J.C., and Bueren, J.A. (2006). Hematopoietic dysfunction in a mouse model for Fanconi anemia group D1. Mol. Ther. *14*, 525–535.

Nestorowa, S., Hamey, F.K., Pijuan Sala, B., Diamanti, E., Shepherd, M., Laurenti, E., Wilson, N.K., Kent, D.G., and Göttgens, B. (2016). A single-cell resolution map of mouse hematopoietic stem and progenitor cell differentiation. Blood *128*, e20–e31.

Niederkorn, M., Hueneman, K., Choi, K., Varney, M.E., Romano, L., Pujato, M.A., Greis, K.D., Inoue, J.I., Meetei, R., and Starczynowski, D.T. (2020). TIFAB regulates USP15-mediated p53 signaling during stressed and malignant hematopoiesis. Cell Rep. *30*, 2776–2790.e2776.

Nishi, R., Wijnhoven, P., le Sage, C., Tjeertes, J., Galanty, Y., Forment, J.V., Clague, M.J., Urbe, S., and Jackson, S.P. (2014). Systematic characterization of deubiquitylating enzymes for roles in maintaining genome integrity. Nat. Cell Biol. *16*, 1016–1026, 1011–1018.

Oguro, H., Ding, L., and Morrison, S.J. (2013). SLAM family markers resolve functionally distinct subpopulations of hematopoietic stem cells and multipotent progenitors. Cell Stem Cell *13*, 102–116.

Olsson, A., Venkatasubramanian, M., Chaudhri, V.K., Aronow, B.J., Salomonis, N., Singh, H., and Grimes, H.L. (2016). Single-cell analysis of mixed-lineage states leading to a binary cell fate choice. Nature *537*, 698–702.

Padmanabhan, A., Candelaria, N., Wong, K.K., Nikolai, B.C., Lonard, D.M., O'Malley, B.W., and Richards, J.S. (2018). USP15-dependent lysosomal pathway controls p53-R175H turnover in ovarian cancer cells. Nat. Commun. *9*, 1270.

Papatheodorou, I., Moreno, P., Manning, J., Fuentes, A.M., George, N., Fexova, S., Fonseca, N.A., Füllgrabe, A., Green, M., Huang, N., et al. (2020). Expression Atlas update: from tissues to single cells. Nucleic Acids Res. *48* (*D1*), D77–D83.

Park, I.-K., Qian, D., Kiel, M., Becker, M.W., Pihalja, M., Weissman, I.L., Morrison, S.J., and Clarke, M.F. (2003). Bmi-1 is required for maintenance of adult self-renewing haematopoietic stem cells. Nature *423*, 302–305.

Parmar, K., Kim, J., Sykes, S.M., Shimamura, A., Stuckert, P., Zhu, K., Hamilton, A., Deloach, M.K., Kutok, J.L., Akashi, K., et al. (2010). Hematopoietic stem cell defects in mice with deficiency of Fancd2 or Usp1. Stem Cells *28*, 1186–1195.

Pellin, D., Loperfido, M., Baricordi, C., Wolock, S.L., Montepeloso, A., Weinberg, O.K., Biffi, A., Klein, A.M., and Biasco, L. (2019). A comprehensive single cell transcriptional landscape of human hematopoietic progenitors. Nat. Commun. *10*, 2395.

Peng, Y., Liao, Q., Tan, W., Peng, C., Hu, Z., Chen, Y., Li, Z., Li, J., Zhen, B., Zhu, W., et al. (2019). The deubiquitylating enzyme USP15 regulates homologous recombination repair and cancer cell response to PARP inhibitors. Nat. Commun. *10*, 1224.

Perez-Riverol, Y., Csordas, A., Bai, J., Bernal-Llinares, M., Hewapathirana, S., Kundu, D.J., Inuganti, A., Griss, J., Mayer, G., Eisenacher, M., et al. (2019). The PRIDE database and related tools and resources in 2019: improving support for quantification data. Nucleic Acids Res. *47* (*D1*), D442–D450.

Pilzecker, B., Buoninfante, O.A., van den Berk, P., Lancini, C., Song, J.Y., Citterio, E., and Jacobs, H. (2017). DNA damage tolerance in hematopoietic stem and progenitor cells in mice. Proc. Natl. Acad. Sci. USA *114*, E6875–E6883.

Pritchard, C.E.J., Kroese, L.J., and Huijbers, I.J. (2017). Direct generation of conditional alleles using CRISPR/Cas9 in mouse zygotes. Methods Mol. Biol. *1642*, 21–35.

Puram, R.V., Kowalczyk, M.S., de Boer, C.G., Schneider, R.K., Miller, P.G., McConkey, M., Tothova, Z., Tejero, H., Heckl, D., Järås, M., et al. (2016). Core circadian clock genes regulate leukemia stem cells in AML. Cell *165*, 303–316.

Ravasio, R., Ceccacci, E., Nicosia, L., Hosseini, A., Rossi, P.L., Barozzi, I., Fornasari, L., Zuffo, R.D., Valente, S., Fioravanti, R., et al. (2020). Targeting the scaffolding role of LSD1 (KDM1A) poises acute myeloid leukemia cells for retinoic acid-induced differentiation. Sci. Adv. *6*, eaax2746.

Rhoads, S.N., Monahan, Z.T., Yee, D.S., and Shewmaker, F.P. (2018). The role of post-translational modifications on prion-like aggregation and liquid-phase separation of FUS. Int. J. Mol. Sci. *19*, 886.

Ritchie, M.E., Phipson, B., Wu, D., Hu, Y., Law, C.W., Shi, W., and Smyth, G.K. (2015). limma powers differential expression analyses for RNA-sequencing and microarray studies. Nucleic Acids Res. *43*, e47.

Schepers, K., Campbell, T.B., and Passegué, E. (2015). Normal and leukemic stem cell niches: insights and therapeutic opportunities. Cell Stem Cell *16*, 254–267.

Schubert, M., Klinger, B., Klünemann, M., Sieber, A., Uhlitz, F., Sauer, S., Garnett, M.J., Blüthgen, N., and Saez-Rodriguez, J. (2018). Perturbation-response genes reveal signaling footprints in cancer gene expression. Nat. Commun. *9*, 20.

Schweitzer, K., Bozko, P.M., Dubiel, W., and Naumann, M. (2007). CSN controls NF-kappaB by deubiquitinylation of IkappaBalpha. EMBO J. *26*, 1532–1541.

Schwertman, P., Bekker-Jensen, S., and Mailand, N. (2016). Regulation of DNA double-strand break repair by ubiquitin and ubiquitin-like modifiers. Nat. Rev. Mol. Cell Biol. *17*, 379–394.

Serresi, M., Gargiulo, G., Proost, N., Siteur, B., Cesaroni, M., Koppens, M., Xie, H., Sutherland, K.D., Hulsman, D., Citterio, E., et al. (2016). Polycomb repressive complex 2 is a barrier to KRAS-driven inflammation and epithelial-mesenchymal transition in non-small-cell lung cancer. Cancer Cell *29*, 17–31.

Serresi, M., Siteur, B., Hulsman, D., Company, C., Schmitt, M.J., Lieftink, C., Morris, B., Cesaroni, M., Proost, N., Beijersbergen, R.L., et al. (2018). Ezh2

inhibition in Kras-driven lung cancer amplifies inflammation and associated vulnerabilities. J. Exp. Med. *215*, 3115–3135.

Singatulina, A.S., Hamon, L., Sukhanova, M.V., Desforges, B., Joshi, V., Bouhss, A., Lavrik, O.I., and Pastre, D. (2019). PARP-1 activation directs FUS to DNA damage sites to form PARG-reversible compartments enriched in damaged DNA. Cell Rep. *27*, 1809–1821.e1805.

Sugawara, T., Oguro, H., Negishi, M., Morita, Y., Ichikawa, H., Iseki, T., Yokosuka, O., Nakauchi, H., and Iwama, A. (2010). FET family proto-oncogene Fus contributes to self-renewal of hematopoietic stem cells. Exp. Hematol. *38*, 696–706.

Teyra, J., Singer, A.U., Schmitges, F.W., Jaynes, P., Kit Leng Lui, S., Polyak, M.J., Fodil, N., Krieger, J.R., Tong, J., Schwerdtfeger, C., et al. (2019). Structural and functional characterization of ubiquitin variant inhibitors of USP15. Structure *27*, 590–605.e595.

Torre, S., Polyak, M.J., Langlais, D., Fodil, N., Kennedy, J.M., Radovanovic, I., Berghout, J., Leiva-Torres, G.A., Krawczyk, C.M., Ilangumaran, S., et al. (2017). USP15 regulates type I interferon response and is required for pathogenesis of neuroinflammation. Nat. Immunol. *18*, 54–63.

Urbé, S., Liu, H., Hayes, S.D., Heride, C., Rigden, D.J., and Clague, M.J. (2012). Systematic survey of deubiquitinase localization identifies USP21 as a regulator of centrosome- and microtubule-associated functions. Mol. Biol. Cell *23*, 1095–1103.

Vasanthakumar, A., Arnovitz, S., Marquez, R., Lepore, J., Rafidi, G., Asom, A., Weatherly, M., Davis, E.M., Neistadt, B., Duszynski, R., et al. (2016). Brca1 deficiency causes bone marrow failure and spontaneous hematologic malignancies in mice. Blood *127*, 310–313.

Vlasschaert, C., Xia, X., Coulombe, J., and Gray, D.A. (2015). Evolution of the highly networked deubiquitinating enzymes USP4, USP15, and USP11. BMC Evol. Biol. *15*, 230.

Wang, J., Sun, Q., Morita, Y., Jiang, H., Gross, A., Lechel, A., Hildner, K., Guachalla, L.M., Gompf, A., Hartmann, D., et al. (2012). A differentiation checkpoint limits hematopoietic stem cell self-renewal in response to DNA damage. Cell *148*, 1001–1014.

Wang, W.Y., Pan, L., Su, S.C., Quinn, E.J., Sasaki, M., Jimenez, J.C., Mackenzie, I.R., Huang, E.J., and Tsai, L.H. (2013). Interaction of FUS and HDAC1 regulates DNA damage response and repair in neurons. Nat. Neurosci. *16*, 1383–1391.

Warr, M.R., Pietras, E.M., and Passegué, E. (2011). Mechanisms controlling hematopoietic stem cell functions during normal hematopoiesis and hematological malignancies. Wiley Interdiscip. Rev. Syst. Biol. Med. *3*, 681–701.

Wijnhoven, P., Konietzny, R., Blackford, A.N., Travers, J., Kessler, B.M., Nishi, R., and Jackson, S.P. (2015). USP4 auto-deubiquitylation promotes homologous recombination. Mol. Cell *60*, 362–373.

Wilson, A., Laurenti, E., Oser, G., van der Wath, R.C., Blanco-Bose, W., Jaworski, M., Offner, S., Dunant, C.F., Eshkind, L., Bockamp, E., et al. (2008). Hematopoietic stem cells reversibly switch from dormancy to self-renewal during homeostasis and repair. Cell *135*, 1118–1129.

Yau, R., and Rape, M. (2016). The increasing complexity of the ubiquitin code. Nat. Cell Biol. *18*, 579–586.

Ye, Z., Yu, X., and Cheng, L. (2008). Lentiviral gene transduction of mouse and human stem cells. Methods Mol. Biol. *430*, 243–253.

Yeung, J., and So, C.W. (2009). Identification and characterization of hematopoietic stem and progenitor cell populations in mouse bone marrow by flow cytometry. Methods Mol. Biol. *538*, 301–315.

Zou, Q., Jin, J., Hu, H., Li, H.S., Romano, S., Xiao, Y., Nakaya, M., Zhou, X., Cheng, X., Yang, P., et al. (2014). USP15 stabilizes MDM2 to mediate cancer-cell survival and inhibit antitumor T cell responses. Nat. Immunol. *15*, 562–570.

Zou, Q., Jin, J., Xiao, Y., Zhou, X., Hu, H., Cheng, X., Kazimi, N., Ullrich, S.E., and Sun, S.C. (2015). T cell intrinsic USP15 deficiency promotes excessive IFN-γ production and an immunosuppressive tumor microenvironment in MCA-induced fibrosarcoma. Cell Rep. *13*, 2470–2479.

Zuber, J., Shi, J., Wang, E., Rappaport, A.R., Herrmann, H., Sison, E.A., Magoon, D., Qi, J., Blatt, K., Wunderlich, M., et al. (2011). RNAi screen identifies Brd4 as a therapeutic target in acute myeloid leukaemia. Nature *478*, 524–528.

CellPress
OPEN ACCESS

STAR★METHODS

KEY RESOURCES TABLE

REAGENT or RESOURCE	SOURCE	IDENTIFIER
Antibodies		
Lineage Cell Detection Mixture-Biotin, mouse	Miltenyi Biotech	Cat#130-092-613
Streptavidin APC/Cy7	Southern Biotech	Cat#7100-19S
cKit-APC	eBioscience	Cat#17-1171-83
Sca1-PerCp-Cy5.5	Biolegend	Cat#108124
CD48-PE-Dazzle 594	Biolegend	Cat#103438
CD135-PE	Biolegend	Cat#135305
CD150-PE-Cy7	Biolegend	Cat#115913
CD16/32-PE-Cy7	eBioscience	Cat#25-0161
CD34-FITC	eBioscience	Cat#11-0341
CD45.1(Ly5.1)-EF450	eBioscience	Cat#48-0453
CD45.2(Ly5.2)-PE	Biolegend	Cat#12-0454-82
CD45.1(Ly5.1)-PE	BD Biosciences	Cat#553776
CD45.2(Ly5.2)-PE-Cy7	Biolegend	Cat#109829
CD3-FITC	Biolegend	Cat# 100203
CD19-APC	BD Biosciences	Cat#550993
Gr1-APC-Cy7	Biolegend	Cat#108423
CD11b-PerCp-Cy5.5	BD Biosciences	Cat#561114
CD4-APC	Biolegend	Cat#100516
CD8a-PerCp-C5.5	BD Biosciences	Cat#551162
CD19-APC-H7	BD Biosciences	Cat#560143
CD43 (Ly-48) MicroBeads, mouse	Miltenyi Biotech	Cat#130-049-801
CD43-biotin (clone S7, RUO)	BD Biosciences	Cat#553269
B220-Pacific Blue	BD Biosciences	Cat#558108
CD45.2-FITC	Biolegend	Cat#11-0454-82
Anti-phospho-Histone H2A.X (Ser139) Antibody, clone JBW301	Millipore	Cat#05-636-I; RRID:AB_2755003
Rabbit Polyclonal anti-53BP1 antibody	Novus Biologicals	Cat#NB100-304; RRID:AB_1659862
Mouse Monoclonal Anti-USP15 antibody	Abcam	Cat#ab56900
Rabbit Polyclonal Anti-USP15 antibody	Abcam	Cat#ab71713
Rabbit Polyclonal Anti-FUS antibody	Novus Biologicals	Cat# NB100-565
Alpha-Tubulin Mouse monoclonal antibody	Sigma- Aldrich	Cat#T90026
Goat anti-mouse HRP	Life technologies	Cat# 626520
Normal Rabbit IgG 2729S	Cell Signaling Technology	Cat#2729S
Alexa Fluor 568 goat anti–mouse	Life Technologies	Cat#A-11004
Alexa Fluor 488 goat anti–rabbit	Life Technologies	Cat#A-11008
Chemicals, Peptides, and Recombinant Proteins		
Mitomycin C	Santa Cruz Biotechnology, Inc.	Cat# sc-3514
Topotecan	Santa Cruz Biotechnology, Inc.	Cat# sc-204919A
Resazurin Sodium Salt	Sigma	Cat# R7017
Bortezomid	Biomol	Cat#Cay10008822
Cisplatin solution	Accord-Healthcare	https://www.accord-healthcare.com/
Recombinant murine SCF	PrepoTech	Cat# 250-03
Recombinant murine TPO	PrepoTech	Cat# 315-14
Recombinant murine Flt3 ligand	PrepoTech	Cat#250-31L

(Continued on next page)

Continued

REAGENT or RESOURCE	SOURCE	IDENTIFIER
Critical Commercial Assays		
4D-Nucleofector® Kit	Lonza	Cat# V4XP-3032
USP15 Gene Knockout Kit V2 (MV4-11 cells genome editing, Figures 7H and 7I).	Synthego	N/A
DNeasy Blood & Tissue Kit	QIAGEN	Cat# 69504
FuGENE® HD Transfection Reagent	Promega	Cat# E2311
AlamarBlueTM Cell Viability reagent	Thermo Fisher Scientific	Cat#DAL1100
Streptavidin MicroBeads	Miltenyi Bio- tec	Cat#130-074-101
LS MACS® Columns for magnetic cell isolation	Miltenyi Bio- tec	Cat# 130-042-401
AMPure XP solid-phase reversible immobilization kit (SPRI)	Beckman Coulter	Cat# A63881
Deposited Data		
RNA sequencing data from K562 and KBM7 cells upon USP15 knockdown by siRNAs	This paper	GEO: GSE160524
RNA sequencing data from LSK cells from *Usp15*$^{+/+}$ and *Usp15*−/− mice	This paper	GEO: GSE160525
RNA sequencing data from LSK cells and B cells from Wild Type mice	Lancini et al., 2016	GEO: GSE58495
Mass spectrometry data, USP15 interactome	This paper	ProteomeXchange: PXD020612
CCLE DeMap v19q1	Broad Institute	https://depmap.org/portal/download/
cBioPortal	Cerami et al., 2012	https://www.cbioportal.org/
Cancer Cell Line Encyclopedia (CCLE)	Broad Institute	https://portals.broadinstitute.org/ccle
Database of hematopoietic cells in health and disease	Hemaexplorer	http://servers.binf.ku.dk/bloodspot/
Single Cell Expression Atlas	Papatheodorou et al., 2020	https://www.ebi.ac.uk/gxa/sc/home
Experimental Models: Cell Lines		
KBM7 chronic myelogenous leukemia (CML) cells	Dr. Thijn R. Brummelkamp lab (NKI, Amsterdam)	Blomen et al., 2015
K562 chronic myelogenous leukemia (CML) cells	Dr. Thijn R. Brummelkamp lab (NKI, Amsterdam)	Blomen et al., 2015
MV4-11 Acute myeloid leukemia (AML) cells	Dr. Saverio Minucci lab (IEO, Milan)	Ravasio et al., 2020
Kasumi 1 Acute myeloid leukemia (AML) cells	Dr. Saverio Minucci lab (IEO, Milan)	Ravasio et al., 2020
MONO-MAC 6 Acute myeloid leukemia (AML) cells	Dr. Saverio Minucci lab (IEO, Milan)	Ravasio et al., 2020
GDM-1 Acute myeloid leukemia (AML) cells	Dr. Saverio Minucci lab (IEO, Milan)	Ravasio et al., 2020
HL-60 Acute myeloid leukemia (AML) cells	Dr. Saverio Minucci lab (IEO, Milan)	Ravasio et al., 2020
GF-D8 Acute myeloid leukemia (AML) cells	Dr. Saverio Minucci lab (IEO, Milan)	Ravasio et al., 2020
THP-1 Acute myeloid leukemia (AML) cells	Dr. Saverio Minucci lab (IEO, Milan)	Ravasio et al., 2020
KG-1 Acute myeloid leukemia (AML) cells	Dr. Saverio Minucci lab (IEO, Milan)	Ravasio et al., 2020
KG-1A Acute myeloid leukemia (AML) cells	Dr. Saverio Minucci lab (IEO, Milan)	Ravasio et al., 2020
ML-2 Acute myeloid leukemia (AML) cells	Dr. Saverio Minucci lab (IEO, Milan)	Ravasio et al., 2020
MONO-MAC-1 Acute myeloid leukemia (AML) cells	Dr. Saverio Minucci lab (IEO, Milan)	Ravasio et al., 2020
OCI-AML2 Acute myeloid leukemia (AML) cells	Dr. Saverio Minucci lab (IEO, Milan)	Ravasio et al., 2020
OCI-AML5 Acute myeloid leukemia (AML) cells	Dr. Saverio Minucci lab (IEO, Milan)	Ravasio et al., 2020
M-07e Acute myeloid leukemia (AML) cells	Dr. Saverio Minucci lab (IEO, Milan)	Ravasio et al., 2020
EOL-1 Acute myeloid leukemia (AML) cells	Dr. Saverio Minucci lab (IEO, Milan)	Ravasio et al., 2020
PLB-985 Acute myeloid leukemia (AML) cells	Dr. Saverio Minucci lab (IEO, Milan)	Ravasio et al., 2020
SKNO-1 Acute myeloid leukemia (AML) cells	Dr. Saverio Minucci lab (IEO, Milan)	Ravasio et al., 2020
MOLM-13 Acute myeloid leukemia (AML) cells	Dr. Saverio Minucci lab (IEO, Milan)	Ravasio et al., 2020
PL-21 Acute myeloid leukemia (AML) cells	Dr. Saverio Minucci lab (IEO, Milan)	Ravasio et al., 2020
MOLM-14 Acute myeloid leukemia (AML) cells	Dr. Saverio Minucci lab (IEO, Milan)	Ravasio et al., 2020

(*Continued on next page*)

Continued

REAGENT or RESOURCE	SOURCE	IDENTIFIER
NB4	Dr. Saverio Minucci lab (IEO, Milan)	Ravasio et al., 2020
Primary mouse Non-small Cell Lung Cancer (NSCLC) KPE cells KrasG12D/+;Trp53−/−;EED−/− genetic background	Dr. Michela Serresi, Dr. Gaetano Gargiulo (MDC, Berlin)	Serresi et al., 2016
U2OS osteosarcoma cells	Dr. Maarten van Lohuizen lab (NKI, Amsterdam)	N/A
Experimental Models: Organisms/Strains		
Mouse: Usp15 knockout	This paper	MGI: Usp15 < em1Nki > ; MGI:5810631; B6J-Usp15 < em1Nki >
Mouse: C57BL/6J-Ly5.2 (C57BL/6J) Wild Type	The Jackson Laboratory	Stock No: 000664
Mouse: C57BL/6-Ly5.1 Wild Type	The Jackson Laboratory	Stock No: 002014
Oligonucleotides		
Primer sequences for Illumina sequencing multiplexing strategy: see Table S4	This paper	N/A
Mouse Usp15 knockout: 5′ CRISPR-guide (gRNA): TCTTCTTCCACTAGCCGTAGCGG	This paper	N/A
Mouse Usp15 knockout 3′: CRISPR-guide (gRNA): GTCACTTGATACGATAGCGCCGG	This paper	N/A
Mouse Usp15: Forward: 5′-TCCAGTAGGAGTGAACCCGC-3′	This paper	N/A
Mouse Usp15: Reverse knockout allele: 5′-AGGTGGCTGAGAGTGAGAGCAGG-3′	This paper	N/A
Mouse Usp15, Reverse Wild type allele: 5′-GCCTTCCGCCATCTTCTTCCAC-3′	This paper	N/A
Human USP15 siRNAs: siGENOME Human USP15 (9958) *siRNA-SMART pool*	Dharmacon	M-006066-01
siGENOME Non-Targeting siRNA Control Pool#2	Dharmacon	D-001206-14-05
Human USP15 shRNA#1: TAAACCAGCATCCTGAATGG	This paper	N/A
Human USP15 shRNA#2: TTTCATGAACTCAGCTATTC	This paper	N/A
Human sgRNA (sg01) targeting sequence: *USP15*ex3, 5′-AAGGTGTTCCTTAAGTGACT-3′ (U2OS cells genome editing; Figure S7).	Human Brunello CRISPR knockout pooled library; CRISPR Design; CRISPRscan	Addgene #73178; http://crispr.mit.edu/http://www.crisprscan.org
Human USP15, Pair of complementary DNA oligos: *USP15* Forward 5′-caccgAAGGTGTTCCTTAAGTGACT-3′ (U2OS cells genome editing; Figure S7).	This paper	N/A
Human USP15, Pair of complementary DNA oligos: *USP15* Reverse 5′-aaacAGTCACTTAAGGAACACCTTc-3′ (U2OS cells genome editing; Figure S7).	This paper	N/A
ASK-FN2 5′-CGGCCTTTTTACGGTTCCTG-3′ (U2OS cells genome editing; Figure S7).	Addgene	https://www.addgene.org/53062/sequences/
TIDE analysis: Human genomic region surrounding USP15 gRNA-targeted region: Forward 5′-GTTAGTGTTACAATTCTTCCAATACGG-3′	This paper	N/A
TIDE analysis: Human genomic region surrounding USP15 gRNA-targeted region: Reverse, 5′GTTTTATCAAAAACAGTGCAGCACAG-3′	This paper	N/A
TIDE analysis: Sanger sequencing Primer 5′-TTACAATTCTTCCAATACGGCCCAG-3′	This paper	N/A

(*Continued on next page*)

Continued		
REAGENT or RESOURCE	SOURCE	IDENTIFIER
qRT-PCR – mouse HPRT Forward: 5′- CTGGTGAAAGGACCTCTCG-3′	This paper	N/A
qRT-PCR - mouse HPRT Reverse: 5′-TGAAGTACTCATTATAGTCAAGGGCA-3′	This paper	N/A
qRT-PCR mouse Usp15 A Forward: 5′-TGTGGCTTAAGTAACTTGGGAAA-3′	This paper	N/A
qRT-PCR mouse Usp15 A Reverse: 5′-AAGTGGAGGTGTGTTGCTCA-3′	This paper	N/A
qRT-PCR mouse Usp15 B Forward: 5′-TCAGCTGGTACACACTGATGG-3′	This paper	N/A
qRT-PCR mouse Usp15 B Reverse: 5′-TGCTTTACAAACATACCCTGTTCT-3′	This paper	N/A
Primers used in Figures S6G and S6H for validation of RNA-seq results are available upon request.	This paper	N/A
Recombinant DNA		
RNAi Consortium library (TRC Mm1.0)	Sigma-Aldrich, MO	MISSION® *TRC Mm1.0*)
pLKO.1-puro	Sigma-Aldrich, MO	SHC001
pLKO.1-puro USP15sh15	Sigma-Aldrich, MO	TRCN0000033215
pLKO.1-puro USP15sh16	Sigma-Aldrich, MO	TRCN0000033216
pLKO.1-puro USP15sh17	Sigma-Aldrich, MO	TRCN0000033217
pSpCas9(BB)-2A-GFP (PX458) plasmid	Addgene	Cat#48138
Plasmid: pLV[Exp]-Puro-H1/TO > hUSP15shRNA#1-UBC > TetR(ns):T2A:EGFP	This paper	N/A
Plasmid: pLV[Exp]-Puro-H1/TO > hUSP15shRNA#2-UBC > TetR(ns):T2A:EGFP	This paper	N/A
Software and Algorithms		
TIDE software	Dr. Bas van Steensel lab (NKI, Amsterdam)	https://tide.nki.nl/
MaxQuant software package version 1.6.3.4	Max Planck Institute of Biochemistry (Cox and Mann, 2008)	https://www.maxquant.org
Reactome pathway analysis tool v3.7, database release 73	Fabregat et al., 2017	https://reactome.org/
R v3.5	https://cran.r-project.org/	https://cran.r-project.org/
Progeny v1.6	Schubert et al., 2018	https://bioconductor.org/packages/release/bioc/html/progeny.html
Limma v3.36	Ritchie et al., 2015	https://bioconductor.org/packages/release/bioc/html/limma.html
ImageJ software (version:2.0)	NIH; Dr. Bram van den Broek (NKI, Amsterdam)	https://imagej.nih.gov/ij/
FACS data analysis	FlowJo	https://www.flowjo.com/ FlowJo Software version 10.0.8r1. (Tree Star)
RNA-seq mapping pipeline	TopHat2.1, Genome build 38 Ensembl gtf version 77	https://ccb.jhu.edu/software/tophat/index.shtml
RNA-seq differential expression analysis	R package	R; DEGseq 10.18129/B9.bioc.DEGseq
RNA-seq genecounts	Itreecount	https://github.com/NKI-GCF/itreecount

(*Continued on next page*)

Continued

REAGENT or RESOURCE	SOURCE	IDENTIFIER
Prism 7.0	GraphPad	https://www.graphpad.com/scientific-software/prism/
CRISPR/Cas9 design tools	CRISPR Design; CRISPRscan	http://crispr.mit.edu/http://www.crisprscan.org
Other		
X-ray irradiation	Faxitron	MultiRad 225 X-ray irradiation system
Time-lapse imaging and proliferation assays	Essen BioScience	IncuCyte FRL

RESOURCE AVAILABILITY

Lead Contact

Further information and requests for resources and reagents should be directed to and will be fulfilled by the lead author Elisabetta Citterio (elisabetta.citterio@gmail.com).

Materials Availability

Plasmid generated in this study and primers sequences are available upon request.

Data and Code Availability

The sequencing data discussed in this publication have been deposited in NCBI's Gene Expression Omnibus (Edgar et al., 2002) and are accessible through GEO Series accession numbers GSE160524 (https://www.ncbi.nlm.nih.gov/geo/query/acc.cgi?acc=GSE160524) and GSE160525 (https://www.ncbi.nlm.nih.gov/geo/query/acc.cgi?acc=GSE160525).

The mass spectrometry proteomics data are available via ProteomeXchange with identifier PXD020612.

EXPERIMENTAL MODELS AND SUBJECT DETAILS

Mice: generation and breeding of USP15 knockout mice

This study utilized murine animal models, consisting of adult mice between 4 and 18 weeks of age. Age and sex matched mice were used in experimental settings, as specified.

Full *Usp15* knockout (KO) mice (MGI: Usp15 < em1Nki > ; MGI:5810631; B6J-Usp15 < em1Nki >) were generated by CRISPR-Cas9-mediated deletion of the *Usp15* locus in C57BL/6J zygotes as described (Pritchard et al., 2017). Two CRISPR-guides (gRNAs) were used that target Cas9 mediated double stranded DNA cleavage at both the 5′ and 3′ UTR of *Usp15*. The sequence of the 5′ and 3′ targets were TCTTCTTCCACTAGCCGTAGCGG and GTCACTTGATACGATAGCGCCGG, respectively. The expected cleavage sites (underlined) are 91.795 bp apart in the C57BL/6J genome (Figure S4A). Mice carrying a full *Usp15* knockout (KO) allele, in which the 91 kb of cleavage site intermitting sequence is missing, were identified by PCR and sequence analysis. The *Usp15* alleles were detected with the following primers: forward, 5′-TCCAGTAGGAGTGAACCCGC-3′; reverse KO, 5′-AGGTGGCTGAGAGTGAGAG-CAGG-3′; reverse wt, 5′-GCCTTCCGCCATCTTCTTCCAC-3′, yielding a product of 590 bp and 396 bp for *Usp15*-KO or *Usp15* wt, respectively. The predicted CRISPR-Cas9-mediated fusion product for the *Usp15*-KO allele is: ccgcta.c.tatcgtat. The 590bp *Usp15-KO* PCR fragment was sequenced, yielding the obtained fusion product: ccgtaTcGGatcgtat. Genotyping was performed by PCR of genomic tail DNA using the Extract PCR kit (Bioline, cat. No. BIO-21127). All mice were kept on C57BL/6J (The Jackson Laboratory) strain background (CD45.2^{+}) in a specific pathogen-free environment. *Usp15* mice were maintained heterozygous. All animal experiments comply with Dutch and European regulations and ethical guidelines and have been authorized by our local experimental animal committee at the Netherlands Cancer Institute (DEC-NKI). For determining survival, mice time to death was defined as the latency between birth and unexpected death or a terminal disease stage indicated by > 20% weight loss or other symptoms of severe sickness. Mice were sacrificed by CO_2 asphyxiation and underwent necroscopy. Organs were collected and fixed for histopathological analysis as described (Lancini et al., 2014).

Leukemia cells

KBM7 were grown in IMDM (GIBCO) medium. K562 were grown in RPMI 1640 (GIBCO) medium (Blomen et al., 2015). Culture medium was supplemented with 2 mM L-Glutamine (GIBCO 25030-164), 10% fetal calf serum (FCS, Thermo Scientific) 100 U ml^{-1} penicillin, and 100 μg ml^{-1} streptomycin (Pen/Strep GIBCO 15140-163). Cells were incubated at 37°C in a humidified atmosphere containing 5% CO_2.

NB4, HL60, THP-1, ML-2, MV4-11, EOL-1, PLB-985, KASUMI were grown in RPMI 1640(GIBCO), 10% fetal bovine serum (South American Origin, Thermofisher), 100 U ml−1 penicillin, and 100 μg ml−1 streptomycin (Pen/Strep GIBCO) 2mM of L-glutamine (GIBCO)

GDM-1, GF-D8, MOLM-13, PL-21, MOLM-14 were grown in RPMI 1640 (GIBCO), 20% fetal bovine serum (South American Origin, Thermofisher), 100 U ml−1 penicillin, and 100 μg ml−1 streptomycin (Pen/Strep GIBCO), 2mM of L-glutamine (GIBCO) (Ravasio et al., 2020).

OCI-AML5 were grown in alpha-MEM (GIBCO), 20% fetal bovine serum (South American Origin, Thermofisher), 100 U ml−1penicillin, and 100 μg ml−1 streptomycin (Pen/Strep GIBCO), 2mM of L-glutamine (GIBCO), 10 ng/ml GM-CSF

M-O7E were grown in in RPMI 1640 (GIBCO), 20% fetal bovine serum (South American Origin, Thermofisher), 100 U ml−1penicillin, and 100 μg ml−1 streptomycin (Pen/Strep GIBCO), 2mM of L-glutamine (GIBCO), 10 ng/ml GM-CSF

SKNO-1985 were grown in RPMI 1640(GIBCO), 10% fetal bovine serum (South American Origin, Thermofisher), 100 U ml−1 penicillin, and 100 μg ml−1 streptomycin (Pen/Strep GIBCO) 2mM of L-glutamine (GIBCO)), 10 ng/ml GM-CSF

OCI-AML2 were grown in alpha-MEM (GIBCO), 20% fetal bovine serum (South American Origin, Thermofisher), 100 U ml−1 penicillin, and 100 μg ml−1 streptomycin (Pen/Strep GIBCO), 2mM of L-glutamine (GIBCO)

KG1, KG- 1A were grown in RPMI 1640(GIBCO), 10% fetal bovine serum (South American Origin, Thermofisher), 100 U ml−1 penicillin, and 100 μg ml−1 streptomycin (Pen/Strep GIBCO) 2mM of L-glutamine (GIBCO), 1mM Sodium Pyruvate (GIBCO) and HEPES 10 mM

Mono-Mac1 were grown in RPMI 1640(GIBCO), 10% fetal bovine serum (South American Origin, Thermofisher), 100 U ml−1 penicillin, and 100 μg ml−1 streptomycin (Pen/Strep GIBCO) 2mM of L-glutamine (GIBCO), 1mM Sodium Pyruvate (GIBCO) and 0.1 mM Non Essential Amino Acids (NEAA, GIBCO)

Mono-Mac6 were grown in RPMI 1640(GIBCO), 10% fetal bovine serum (South American Origin, Thermofisher), 100 U ml−1 penicillin, and 100 μg ml−1 streptomycin (Pen/Strep GIBCO) 2mM of L-glutamine (GIBCO), 1mM Sodium Pyruvate (GIBCO) and 0.1 mM Non Essential Amino Acids (NEAA, GIBCO) and 9 ug/ml Insulin.

KPE cell line

Primary mouse Non-small Cell Lung Cancer (NSCLC) KPE cells were isolated from KrasG12D/$^{+}$;Trp53−/−;EED−/− genetic background as described (Serresi et al., 2016). Cells were propagated in DMEM/F12 medium supplemented with 10% FBS, and 5% penicillin and streptomycin, 4ug/ml of hydrocortisone (Sigma), 5 ng/ml murine EGF (Invitrogen), Insulin-Transferrin-Selenium mix/solution (GIBCO) and incubated at 37°C in a 5% CO2%–95% air incubator.

U2OS cell line

U2OS osteosarcoma cells were grown in Dulbecco's modified Eagle medium (DMEM; GIBCO), supplemented with 10% fetal calf serum (FCS, Thermo Scientific), 100 U ml^{-1} penicillin, and 100 μg ml^{-1} streptomycin (Pen/Strep GIBCO 15140-163). Cells were incubated at 37°C in a humidified atmosphere containing 5% CO_2.

METHOD DETAILS

shRNA libraries

Lentiviral hairpins (pLKO.1) targeting annotated DUB genes(Mevissen and Komander, 2017) and controls were selected (Table S1). Vectors were individually picked from glycerol stocks of The RNAi Consortium library (TRC Mm1.0) (Sigma-Aldrich, MO), grown up on agar plates and combined before maxiprep DNA isolation. Pooled plasmid libraries were used to produce lentiviral particles using standard procedures (Gargiulo et al., 2014).

In vivo shRNA screens

Lineage negative (Lin-) hematopoietic stem and progenitors cells were isolated from the bone marrow (BM) of 8-10 weeks old wild-type (wt) mice (C57BL/6J-Ly5.2)(CD45.2) and plated in serum-free medium supplemented with cytokines as described below.

Puromycin selection and MOI calculations were performed following our previously set up and validated protocol for *in vivo* RNAi screens (Gargiulo et al., 2014). We adapted this protocol to mouse HSPC cells. Specifically, we first tested the sensitivity of HSPCs to puromycin. We titrated puromycin in wild-type freshly isolated mouse lineage negative (lin-) cells in order to determine the optimal concentration for selection, which we set at 1 μg/ml for 48 hours. This concentration was used for selection of Lin-cells infected with the titered shRNA DUB libraries. Live cells were counted before and after puromycin selection using TC20 Automated cell counter BIORAD and trypan blue.

Viral titer of the DUB shRNA libraries was first determined by serial dilution on 293T cells followed by 48 hours puromycin selection (1 μg/ml) and Alamar blue cell viability assay. Freshly isolated Lin- cells were counted and a virus MOI < < 1 was calculated for the infection (Gargiulo et al., 2014). Upon viral library infection (MOI < < 1) and puromycin selection we typically obtained 50%–80% live transduced cells after 48 hours, while control un-transduced cells were visually distressed and trypan blue-positive.

Specifically for the screens, Lin- cells were pre-stimulated for 24 hours (hr) and transduced with pooled lentiviral shRNAs at low multiplicity of infection (MOI < 0.5) using spin-inoculation at 1,800 rpm for 90 min at 32°C. Twenty-four hours after infection, the cells

stably expressing integrated shRNA were selected with puromycin (1 μg/ml). 48 hours later, cells were harvested, keeping 1×10^6 cells for DNA extraction of the INPUT(T0) sample. Next, $2x10^6$ Lin- cells for the primary screening or $1x10^6$ for the secondary screens (representing a minimum of 3,500-fold enrichment over the library), were mixed with $1x10^6$ freshly isolated BM cells from wt (C57BL/6-Ly5.1)(CD45.1) mice and injected into recipient mice as described below. Recipient mice were sacrificed at 4 weeks post transplantation (wpt) and femurs, tibia, and spleen were collected. Lin- cells from BM and CD43-, $CD19^+$, $CD220^+$, CD45.2 splenocytes were purified as described below and genomic DNA was extracted for PCR amplification of the shRNAs.

PCR and next-generation sequencing

shRNAs sequences were retrieved from genomic DNA by PCR amplification as described (Gargiulo et al., 2014). For every sample, a maximum of 8 μg genomic DNA was divided over 4 50 μL PCR reactions using barcoded forward primers (PCR1). The products of all reactions were pooled and a maximum of 1 μg from this PCR1 was used per reaction in subsequent PCR2 reactions using primers containing Indexes for next-generation sequencing. Barcodes and Indexes for deep sequencing (Illumina HiSeq 2000) were incorporated into PCR primers as listed in Table S4.

PCR mixture per reaction: 10 μL HF Buffer (NEB), 1.25 μL 10-μM forward primer, 1.25 μL 10-μM reverse primer, 1.25 μL Phusion Hot Start II polymerase (2U μl^{-1}; Thermo Scientific, cat.n. F-530L), 1 μL 10-mM dNTPs, DMSO 3% (vol/vol), adding mQ and template to 50 μl. PCR conditions were: 1' @ 98°C, 16 (PCR1) or 14 (PCR2) x (10 s @ 98°C, 30 s @60°C, 60 s @ 72°C), 5 min @ 72°C. PCR products were purified using the AMPure XP solid-phase reversible immobilization kit (SPRI; Beckman Coulter, cat. no. A63881) and subjected to Illumina next-generation sequencing. The shRNA sequence reads were aligned to the TRC library. Fold change in individual hairpin representation *in vivo* was determined by comparing shRNA representation in each sample to that in the control cell population remaining after tail vein injections during bone marrow transplantation (INPUT, T0). Each condition included in the preliminary analysis was matched to its corresponding shRNA library removing those shRNA that weren't present in any of the samples. Pairwise differential abundance analysis was performed between test sample and input using limma v3.36 (Ritchie et al., 2015) after outlier removal using PCA. shRNA were considered as enriched or dropped out if logFC was higher than $|1|$, adj.Pvalue ≤ 0.02 and avg. abundance > 2.5 . The analysis was done using R v3.5 programming language (https://cran.r-project.org/).

Bone marrow transplantation assays

For RNAi *in vivo* screens, puromycin selected, retroviral-transduced Lin- cells (CD45.2) were mixed with wt bone marrow cells (CD45.1) as described above and injected into lethally irradiated (2 doses of 5.5 Gy TBI separated by an interval of 3 hours) wt C57BL/6-CD45.1 recipient mice. Primary screen was performed with the full shRNA DUB library in five replicate mice. The DUB library was divided in two sub-pools, DUB1 and DUB2 sub-libraries, and two secondary screens were performed, DUB1 in four replicate mice, DUB2 in 7 replicate mice.

Donor contribution was assessed based on the expression of CD45.1/CD45.2 antigens. At 4 wpt primary recipients were sacrificed and the frequency of donor-derived CD45.2 peripheral blood cells, splenocytes, Lin- and LSK were assayed by phenotypic profiling. Lin- cells and CD45.2 splenic B cells for genomic DNA extraction and shRNA retrieval were isolated as described below.

In validation experiments, wt Lin- cells were transduced with individual lentiviral vectors and puromycin-selected as indicated above. The percentage of LSK in the Usp15 wt (shCtrl) and knockdown (shUsp15) transduced, puromycin selected live cell population was assessed by phenotypic profiling before mixing with support wt BM cells. The difference in LSK between shCtrl and shUsp15 transduced cells before mixing and transplantation into recipient mice was corrected in order to transplant LSK equivalents.

$1x10^6$ lentiviral-transduced, puromycin resistant cells (CD45.2) were transplanted together with $1x10^6$ total BM cells (CD45.1) into lethally irradiated recipient mice (CD45.1). Recipient mice peripheral blood was monitored by FACS analysis at 2 wpt and every 4 wk for 18 wk. Donor contribution and multilineage reconstitution were assessed based on the expression, respectively, of CD45.1/CD45.2 antigens or CD19, CD3, and Gr1 markers in the $CD45.2^+$ fraction. At 18 wpt, primary recipients were sacrificed and the frequency of donor-derived CD45.2 B and T cells in the spleen and Lin-, LSK, HSC and myeloid progenitors in the bone marrow was assayed by phenotypic profiling.

In competitive BM transplantation, BM was isolated from donor test animals (CD45.2, *Usp15*$^+$/$^+$ or *Usp15*$-/-$) and mixed in a 1:1 ratio with wt competitor cells (CD45.1)(8-10 weeks old mice). For each genotype and for the wt competitor BM, cells from 3 donors and from 3 wt competitor mice were isolated and pooled before 1:1 mixing. Thereafter, 1×10^6 CD45.1/CD5.2 mixed BM cells were transplanted into lethally irradiated CD45.1 recipient mice as described above. Flow cytometry staining for LSK and HSC of donors was performed to ensure that the HSC frequency in test and control BM would be comparable. Differences in HSC were corrected before transplantation to transplant stem cell equivalents. Chimerism in the blood of primary recipients and BM repopulation at 18 wpt were assessed as described above.

Mice were irradiated using Faxitron MultiRad 225 X-ray irradiation system. Irradiated mice were treated with Enrobactin for the first 4 wk after irradiation. Immunophenotyping, Lin- isolation and CD45.2 splenocytes purification were performed as described below.

Flow cytometry

Peripheral blood was collected into EDTA-coated micro-tubes. For FACS analysis, blood was depleted from red blood cells by hypotonic lysis and staining was performed with fluorochrome-labeled antibodies, CD3-FITC, CD11b-PerCp/Cy5.5, CD19-APC, Gr1-APC/Cy7.

Single cell suspension of spleen was obtained by smashing through a 70 μm filter. Suspension was depleted from red blood cells by hypotonic lysis. For isolation of immature and of mature (resting) B cells, CD43 positive cells were first depleted using anti-mouse CD43 (Ly-48) MicroBeads (MACS Miltenyi Biotec). Donor derived cells were then isolated by FACS sorting using fluorochrome-labeled antibodies CD45.2-FITC, CD45.1-PE, CD19-APC, B220-Pb, CD43-biotin and Streptavidin APC/Cy7. For immunophenotyping of spleen upon transplantation, staining was performed with fluorochrome-labeled antibodies, CD3-FITC, CD8a-PerCp/Cy5.5, CD4-APC, CD19-APCH7.

Analyses and cell sorting of hematopoietic precursors

To analyze Lin-, LSK, HSC, LKS-, CMP, GMP and MPP subpopulations (Cabezas-Wallscheid et al., 2014; Kiel et al., 2005; Oguro et al., 2013; Wilson et al., 2008; Yeung and So, 2009), BM freshly isolated mononuclear cells (MNC) were first stained with Lineage Cell Detection Cocktail-Biotinylated mouse antibody (MACS Miltenyi Biotec). For FACS analysis, cells were then directly stained with fluorochrome-conjugated antibodies. We used 5×10^6 MNCs per staining. For quantifying LSK and HSC populations, cKit-APC, Sca-1-PerCp/Cy5.5, CD48-FITC, CD150-PE/Cy7, CD135-PE and streptavidin-APC/Cy7 antibodies were used. For quantifying LKS^- progenitor populations, cKit-APC, Sca-1-PerCp/Cy5.5, CD34-FITC, CD16/32-PECy7, and streptavidin-APC/Cy7 antibodies were used. For purifying Lin- cells for shRNA library viral infection or for culturing, depletion of $lineage^+$ cells from MNCs was performed using Biotin labeled Lin^+ cocktail and Streptavidin MicroBeads (Macs; Miltenyi Bio- tec) and magnetic columns (Macs; Miltenyi Biotec). For cell sorting of LSK, depletion of $lineage^+$ cells was first performed as above before staining.

Cell cycle analysis of BM populations

Cell surface staining was performed as described above. Samples were then fixated in 3.7% Formaldehyde in PBS (Sigma)) for 30 min at RT. Cells were permeabilised in PBS/BSA(1%)/Tween20 (0.025%)(PBT) for 15min at RT and harvested in PBT containing 10 μg/mL DAPI for chromatin labeling. Cell cycle analysis was performed as described (Pilzecker et al., 2017).

Assessing Cleaved Caspase-3 levels of BM populations

Cell surface staining, fixation and permeabilization was performed as described above followed by staining with Cleaved Caspase-3-AF488 antibody for 30 min at RT in PBT. Cells were washed twice with PBT and harvested in PBT containing 10 μg/mL DAPI for chromatin labeling.

All FACS measurements were performed with a BD LSRFortessa cell analyzer (BD Biosciences). Cell sorting was performed with a FACSAria (BD). All FACS data were analyzed using FlowJo Software version 10.0.8r1. (Tree Star).

Hematopoietic stem and progenitor cells liquid culture, time-lapse imaging and proliferation assays

Mice were sacrificed at the indicated age (8-12 weeks). Lineage negative (Lin-) isolation, LSK FACS sorting and cell surface staining was performed as described above. Cells were plated on Ultra-Low Attachment multiwell plates (Corning®Costar®) in StemPan SFEM (StemSpan Serum-Free Expansion Medium (SFEM) STEMCELL Technologies) supplemented with mouse SCF 100 ng/ml, mouse thrombopoietin (mTpo) 50 ng/ml and mFlt3 ligand 50 ng/ml (PrepoTech) (Ye et al., 2008). Medium was replenished and cells were expanded in 3% oxygen to maintain optimal growth. To evaluate proliferation, 1,000 Lin- cells were plated in 96-well plates at day 7 of culture. 4 wells per conditions were imaged (phase-contrast) with a 4 hr interval for 6.5 d using the IncuCyte FRL (Essen BioScience). Confluence was determined by the IncuCyte software, based on area (confluence) metrics. Plating of 500 cells/well gave similar results. FACS sorted LSK cells from individual animals were grown individually. LSK were plated at day 8 of culture and monitored for growth by counting live cells by Trypan blue exclusion using a TC20™ Automated Cell Couter (BIORAD) at the indicated time. Four wells per condition were counted.

CFU-C colony-forming assay

BM MNCs cells were seeded on 35-mm culture dishes in triplicate in methylcellulose medium supplemented with cytokines (MethoCult GF M3434, STEMCELL Technologies). CFU-Cs (colony forming units in culture) include CFU-GEMM (granulocyte, erythroid, macrophage, megakaryocyte), multipotential progenitors and lineage-restricted progenitors of the erythroid (BFU-E, burst-forming unit–erythroid), and granulocytic, monocyte- macrophage (CFU-GM). Cultures were incubated at 37°C under 5% CO_2. Colonies were quantified at day 8.

Leukemia cells RNA interference and cell viability assays

USP15 siRNAs (siGENOME Human USP15 (9958) siRNA-SMART pool M-006066-01) and control (Ctrl) siRNAs (siGENOME Non-Targeting siRNA Control Pool#2 D-001206-14-05) were from Dharmacon. Cells were transfected using Lipofectamine® RNAiMAX Reagent from Life Technologies following the manufacturer's instructions and assayed at 72 hr after transfection in western blotting, viability assays and immunofluorescence or RNA-seq.

For inducible USP15 knockdown experiments, we have tested 4 shRNAs. To rule out potential off-target effects by one shRNA, we used two different shRNAs for experiments in Figures 6I and 6K: human USP15 shRNA#1 'TAAACCAGCATCCTGAATGG' and shRNA#2 'TTTCATGAACTCAGCTATTC', respectively. The additional shRNA tested were: 'GCATTAGGCTGCCGTATATA', and 'CGCTTATAAGAACTATGATT' and these were found insufficiently potent on-target. The shRNA-containing bicistronic vectors

were either subcloned or synthetized at VectorBuilder, and included a H1 promoter and Tet operator shRNA cassette as well as a Tet repressor-T2A-eGFP for FACS sorting of TetR positive cells.

Viability assays were performed using AlamarBlueTM Cell Viability reagent (Thermo Fisher Scientific). Relative viability was normalized to the control siRNA transfected cells and corrected for back-ground signal.

TCGA pan-cancer gene expression analysis and Single Cell Expression analysis

Gene expression analysis by RNaseq was compiled using data from all TCGA cohorts (Cerami et al., 2012). Gene expression was measured using the IlluminaHiSeq technology. Data from all TCGA cohorts are combined to produce this dataset. Values are PAN-CAN expression unit - (log(norm(exp) $^{+}$ 1)) transformed RSEM values. Single Cell Expression analysis for USP15 was performed using data from the Single Cell Expression Atlas (Papatheodorou et al., 2020) (https://www.ebi.ac.uk/gxa/sc/home).

RNA-seq gene expression analysis

For gene expression analysis, KBM7 or K562 cells were transfected with USP15 or control siRNAs as described above. Total RNA was extracted at 72 h after transfection. For LSKs, cells were FACS sorted from freshly isolated BM and total RNA was extracted. n = 3 *Usp15*$^{+}$/$^{+}$ and n = *2 Usp15−/−* littermates (2 months old). Samples were prepared using TruSeq protocols, and standard sample preparation protocols and RNA-seq was performed on a Hiseq2000 machine (Illumina) at the NKI Genomics Core Facility.

The sequencing data have been deposited in NCBI's Gene Expression Omnibus (Edgar et al., 2002) and are accessible through GEO Series accession numbers GSE160524 and GSE160525.

Sensitivity assays

Cells were cultured in the proper culture medium with doxycycline for 5 days (100ng/ml) to induce USP15 knockdown. Cells were then seeded in 384 well plates 24 hr before treatment. For IR, cells were irradiated with the indicated dose and cell viability was assessed 3 days after IR using a medical irradiator platform (XenX). Similar data were obtained with siUSP15. To determine the 30nM MMC concentration, MMC dose-response experiments were previously assessed and 30nM was determined as the IC_{50} dose for MV4-11 cells in our experimental conditions. Cell viability was assessed at 72 hours. Doxycycline and drug were refreshed daily.

In vivo cisplatin (CsPt) Sensitivity Assay. Mice were injected i.v. with 0.8 mg/kg cisplatin, a relative low dose (Pilzecker et al., 2017), or PBS. After 2 d, the BM was isolated and analyzed as described above.

Immunofluorescence and quantitative image analysis

In vitro cultured murine LSK or human K562 and KBM7 cells were deposited on charged slides (Superfrost Plus; Menzel-Glaser) by cytospin and directly fixed in 4% paraformaldehyde. Immunostaining with antibodies against 53BP1 was performed as previously described (Lancini et al., 2014). Counterstain was with Alexa Fluor 488–conjugated secondary antibodies and DAPI (200 ng/ml) (Sigma-Aldrich). Micronuclei were scored on fixed cells stained with DAPI. Digital images were acquired using a microscope (AxioObserver Z1; Carl Zeiss) with an ORCA-ER CCD charge-coupled device) camera (C4742-80-12AG; Hamamatsu) and Zen software (40x and 63x magnification). A macro in ImageJ software (version:2.0) (developed by Bram van den Broek, NKI, Amsterdam) was used for quantification of spontaneous 53BP1 DNA damage foci. The DAPI channel was used to select the nuclei of the cells in the field. Briefly, Z stacks are converted to two dimensional via one of several user-defined methods: maximum intensity projection, automatically select sharpest slice or manually select a slice. Region of interests (ROIs) of candidate nuclei are then automatically obtained throughout the image stack by auto-thresholding an outlier-removed median-filtered (0.7 mm radius) *z* projection of the nuclei channel, followed by a watershed command to separate touching nuclei and particle analyzer run with size (> 4 and 40 μm2), and circularity (> 0.25) constraints. In the detection of 53BP1 foci, the foci threshold level is defined by the signal-to-noise ratio (SNR): a (user-set) factor times the s.d. of the background fluorescence intensity of the nucleus. The latter property is approximated by first crudely removing signal outliers (the foci), and then taking the median and s.d. of the lower approximate 80% pixel values in the ROI, respectively. The background intensity is subtracted using a Difference of Gaussians filter. Foci are then identified as regions of adjacent pixels with gray values, exceeding the SNR threshold and area larger than a certain minimum. In the procedure, the SNR is the only user-defined parameter, and is iteratively optimized by comparing the detected foci with the original signal in an overlay image.

Protein analysis

Cell were lysed with RIPA buffer (10 mM Tris-Cl (pH 8.0), 1 mM EDTA, 1% Triton X-100, 0.1% sodium deoxycholate, 0.1% SDS, 140 mM NaCl, 1 mM PMSF) containing protease inhibitor cocktail (Complete, Roche) and phosphatase inhibitors 9Na Fluoride 10mM final concentration, Na orthovanadate 1 mM final concentration and NaPPi 1 mM final concentration) and whole cell extract was loaded on SDS-PAGE on NuPAGE gels, followed by western blotting with the indicated antibodies (Table S4). Filter blocking and antibody incubation were performed in PBS supplemented with 0.1(v/v) % Tween and 5%(w/v) bovine milk powder.

For validation of shRNAs, wild-type mouse Non-small Cell Lung Cancer (NSCLC) KPE cells (Serresi et al., 2016) were transduced with individual lentiviral vectors and puromycin-selected for 48hrs as indicated above, followed by protein extraction and immunoblot analysis.

Quantitative real-time (qRT) PCR

Total RNA was extracted using TRIZOL reagent (Life Technologies) or ReliaPrepTM RNA miniprep System (Promega) and cDNA was prepared using Superscript II RT and oligo(dT)$_n$ primers (Life technologies). qRT-PCR was performed on a StepOnePlusRT-PCRsystem(AppliedBiosystems) usingSYBR Green PCR Master Mix (Applied Biosystems). The amount of target, normalized to an endogenous reference (HPRT), was calculated by $2^{-\Delta\Delta C}$T. Primer sequences were as follows: m*Hprt* forward, 5′-CTGGTGAAAGGACCTCTCG-3′; m*Hprt* reverse, 5′-TGAAGTACTCATTATAGTCAAGGGCA-3′; two different pairs of USP15 primers were used, m*Usp15* A forward, 5′- TGTGGCTTAAGTAACTTGGGAAA-3′; m*Usp15* A reverse, 5′-AAGTGGAGGTGTGTTGCTCA-3′; m*Usp15* B forward, 5′-TCAGCTGGTACACACTGATGG-3′; m*Usp15* B reverse, 5′-TGCTTTACAAACATACCCTGTTCT-3′. Primers used in Figure S6G,H for validation of RNA-seq results are available upon request.

Antibodies

Antibody specifications are listed in Tables S5 and S6.

U2OS cells genome editing: sgRNA design and cloning

sgRNA sequences targeting human USP15 were selected from the Human Brunello CRISPR knockout pooled library (Doench et al., 2016) (Addgene #73178) and further selected on the basis of high quality score in two additional online tools: CRISPR Design (https://zlab.bio/guide-design-resources) and CRISPRscan (https://www.crisprscan.org/). The following sgRNA (sg01) targeting exon 3 of USP15 DUSP domain was used in this study: *USP15ex*3, 5′-AAGGTGTTCCTTAAGTGACT-3′. Pairs of complementary DNA oligos (forward: 5′-caccgAAGGTGTTCCTTAAGTGACT-3′; reverse: 5′-aaacAGTCACTTAAGGAACACCTTc-3′) were annealed and the DNA oligonucleotide duplex was cloned in the Bbs1 restriction site of pSpCas9(BB)-2A-GFP (PX458) plasmid (Plasmid #48138, Addgene). sgRNA sequence was verified by DNA Sanger sequencing using the following primer: *ASK-FN2* 5′-CGGCCTTTTTACGGTTCCTG-3′.

Transfection and fluorescence activated cell sorting (FACS)

Plasmids were transfected into U2OS cells using FuGENE® HD Transfection Reagent (Promega), according to the manufacture's instructions. After 48 hours in culture, cells were trypsinized, washed with PBS, and resuspended in PBS (supplemented with 3% BSA) and passed through a cell strainer (Falcon® Round-Bottom Tubes with Cell Strainer Cap, Catalog #38030). Cells were individually sorted (BD FACSAria, BD FACS Diva 8.0.1 sofware) based on EGFP signal into tissue culture 96-well plates (CELLSTAR-Greiner) at a single cell per well for clonal expansion. Viable individual clones were then transferred to 24 well plates for clonal expansion and screening.

MV4-11 cells genome editing and electroporation

Genome editing of MV4-11 cell lines described in the work was performed by electroporation using Amaxa 4D-Nucleofector® Kit accordingly to the manufacturer's instructions. Briefly 2x105 cells were counted and resuspended in SF buffer and supplement and electroporate with the program CM137. To knock out h-USP15 was used Synthego Gene Knockout Kit V2 following the manufacturer's instructions.

Genomic DNA extraction, PCR and Sanger sequencing and TIDE analysis

Genomic DNA was extracted using DNeasy Blood & Tissue Kit (QIAGEN). The human genomic region surrounding USP15 gRNA-targeted sequence was amplified using the following primers: forward, 5′- GTTAGTGTTACAATTCTTCCAATACGG-3′; reverse,5′GTTTTATCAAAAACAGTGCAGCACAG-3′. PCR was performed using Thermo Scientific® Phusion High-Fidelity DNA polymerase in GC buffer. PCR conditions were as follows: 30 s at 95°C, followed by 25 cycles of 30 s at 95°C, 30 s at 64°C, and 30 s at 72°C, followed by 3 min at 72°C. Primer 5′-TTACAATTCTTCCAATACGGCCCAG-3′ was used for Sanger sequencing. About 100-200 ng DNA from purified PCR samples was prepared for sequencing using BigDye terminator v3.1. Samples were analyzed by an Applied Bio- systems 3730x1 DNA Analyzer. The data obtained was analyzed using the TIDE software (https://tide.nki.nl). The decomposition window used for TIDE was set to indels of size 0-10 bp, p threshold of 0.001.

Sample preparation for mass spectrometry

For label-free proteomics analysis, samples were subjected to tryptic on-bead digest as described in Hubner et al. Briefly, washed beads were taken up in digestion buffer (2 M urea buffer / 50 mM Tris pH 7.0 / 1 mM DTT / 5 μg/mL trypsin) and pre-digested for one hour. The supernatant was subjected to reduction (4 mM DTT for 30 min), alkylation (10 mM iodoacetamide for 45 min) and further over-night digest with 0.5 μg trypsin. After desalting, samples were measured by LC-MS/MS on an Orbitrap Exploris 480 mass spectrometer (Thermo) connected to an EASY-nLC system (Thermo). A volume of 2 μl was injected and a 45 min gradient (5 to 55% acetonitrile) was applied. The peptides were separated on an in-house prepared nano-LC column (0.074 mm x 250 mm, 3 μm Reprosil C18, Dr Maisch GmbH) using a flow rate of 250 nL/min. MS acquisition was operated at an MS1 resolution of 60,000 and a scan range from 350 to 1800 m/z. For data-dependent MS2 acquisition a cycle time of 1 s was used and precursors were selected for fragmentation in data-dependent mode using an MS2 resolution of 15,000, a maximum injection time of 100 ms and an isolation width of 1.3 m/z.

Data analysis

For analysis the MaxQuant software package version 1.6.3.4 (Cox and Mann, 2008) was used. Carbamidomethylation on cysteine was set as a fixed modification and oxidized methionine, acetylated N-termini and deamidation on asparagine as well as glutamine were used as variable modifications. An FDR of 0.01 was applied for peptides and proteins and database search was performed using a mouse Uniprot database (July 2018).

The mass spectrometry proteomics data have been deposited to the ProteomeXchange Consortium via the PRIDE (Perez-Riverol et al., 2019) partner repository with the dataset identifier PXD020612.

MS intensities were normalized by the LFQ algorithm while using the match-between-runs feature and separating the cell lines in parameter groups for individual LFQ normalization. Further data analysis was done using R. A number of at least two peptides per protein and three valid values in the USP15 group was required. The resulting list was imputed using a column-wise Gaussian distribution, a width of 0.2 and a downshift of 1.8. Log2-transformed LFQ-intensities among the replicates of the groups to be related were taken for comparison by applying a moderated t test. Proteins with a Benjamini-Hochberg-adjusted p value smaller than 0.1 (i.e., 10% FDR) were considered as significantly enriched (Cox and Mann, 2008; Hubner et al., 2010). From the interactors, proteins with a p-adjusted value < 0.05 compared to IgG, for each condition, were taked into account. The 38 USP15 interactors were analyzed by using Reactome pathway analysis tool (Reactome.org; Pathway browser v3.7, database release 73; https://reactome.org/) (Fabregat et al., 2017).

Immunoprecipitation

For immunoprecipitation previous to mass spectrometry, cells were washed twice in PBS and lysed in RIPA buffer buffer (Thermo Fisher) supplemented with complete EDTA-free protease inhibitor cocktail (Sigma), 1mM Na3VO4, 10mM NaF, 1mM Sodium pyrophosphate and 1mM NEM. For validation immunoprecipitation 0.1% Igepal lysis buffer (150mM NaCl, 20mM Tris pH 7.5, 0.5mM Ethylenediaminetetraacetic acid (EDTA) supplemented with protease and phosphatase inhibitors) was used. Lysates were sonicated in water bath Bioruptor 30 s on/off for 5 cycles and after clearing the lysate by centrifugation, input was taken. USP15 ab71713, FUS or IgG (2.5 ug/mg) antibodies were added and incubated overnight at 4°C in rotation. For precipitation of immunocomplexes, protein A Dynabeads (Thermofisher) were added and incubated for 3h at 4°C in rotation. After extensive washing immunoprecipitates were kept on beads for digestion (Mass spectrometry) or eluted by boiling at 95°C for 5min with SB4x before analysis by SDS-PAGE.

Cellular fractionation

Cells were harvested and washed twice in PBS, lysed in buffer A (10 mM HEPES pH 7.9, 1.5mM MgCl2, 10mM KCl, 0.2% Igepal, 1mM DTT, complete EDTA-free protease inhibitor cocktail (Sigma), 1mM Na3VO4, 10mM NaF and 1mM NEM); incubated 5min on ice and spin down at 1600xg 5 min to extract cytoplasmatic and membrane proteins. The pellet, containing the nuclei, was washed once with buffer A, centrifuged again for 10min and lysed in RIPA buffer (Thermo Fisher) supplemented with protease and phosphatase inhibitors indicated above. Lysates were sonicated in water bath Bioruptor 30 s on/off for 5 cycles and centrifuged for 30min at 13000rpm; the supernatant contained nuclear extract was transferred to a fresh tube. Both extracts were boiled at 95°C for 5min with SB4x.

QUANTIFICATION AND STATISTICAL ANALYSIS

Statistical analysis was performed in GraphPad Prism 7.0, using an unpaired two-tailed Student's t test or Multiple t test were specified. Animal survival experiments were analyzed with a Log-rank nonparametric test and expressed as Kaplan-Meier survival curves. In all Figures: *, $p < 0.05$; **, $p < 0.01$; ***, $p < 0.001$; ****, $p < 0.0001$.

Cell Reports, Volume *33*

Supplemental Information

USP15 Deubiquitinase Safeguards Hematopoiesis and Genome Integrity in Hematopoietic Stem Cells and Leukemia Cells

Paul van den Berk, Cesare Lancini, Carlos Company, Michela Serresi, Maria Pilar Sanchez-Bailon, Danielle Hulsman, Colin Pritchard, Ji-Ying Song, Matthias Jürgen Schmitt, Ellen Tanger, Oliver Popp, Philipp Mertins, Ivo J. Huijbers, Heinz Jacobs, Maarten van Lohuizen, Gaetano Gargiulo, and Elisabetta Citterio

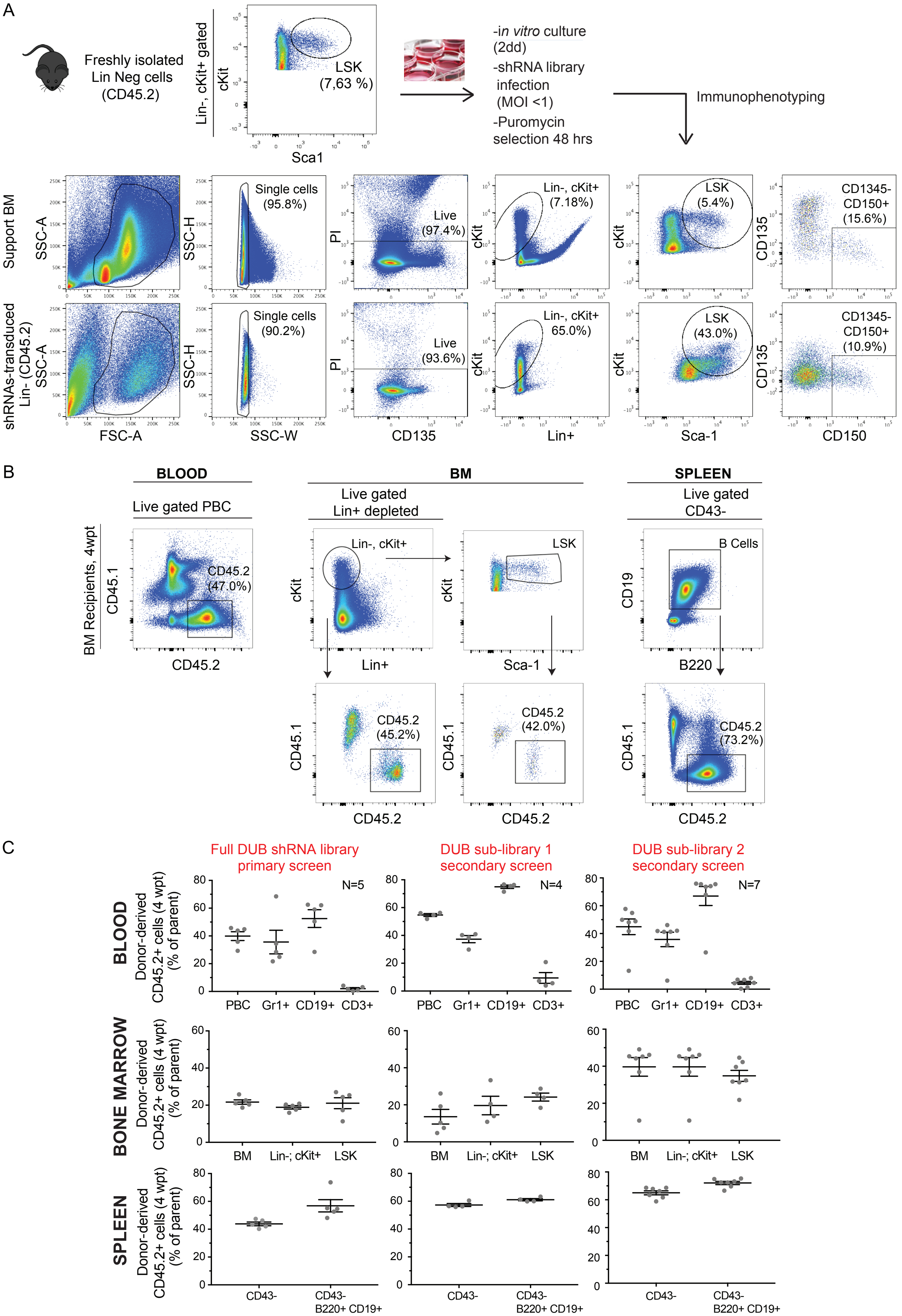

Figure S1. *In vivo* RNAi screens for DUBs in murine hematopoietic stem and progenitor cells, Related to Figure 1.

Figure S1. *In vivo* RNAi screens for DUBs in murine hematopoietic stem and progenitor cells, Related to Figure 1.

(A) Early progenitors (LSK) population is enriched and it is retained upon viral transduction *in vitro*. Lineage negative (Lin-) cells isolated form wt (CD45.2) bone marrow (BM) were grown under stem cell conditions and transduced with pooled shRNA lentiviral libraries targeting all annotated mouse orthologues of human DUBs. After selection, CD45.2 transduced Lin- cells are mixed 1:1 with CD45.1 support BM cells. Top panel: representative FACS profiles and relative percentage of LSK (Lin- Sca1+ cKit+) cells in freshly isolated Lin-, cKit^{high} progenitors. Middle and lower panels: gating strategy and FACS profiles for BM cell subsets in support BM (middle panel) and in shRNA library-transduced Lin-cells prior transplantation (lower panel). LSK were gated on the basis of CD135/Flk-2 and SLAM CD150 surface markers to assess immature precursors endowed with reconstitution potential (LSK, CD135-, CD150+)(Christensen and Weissman, 2001; Kiel et al., 2005; Yeung and Wai Eric So, 2009). Frequency in the parental cell population is shown.

(B-C) ShRNA library-transduced Lin- cells efficiently engraft and contribute to BM repopulation (4 wpt). Representative FACS profiles (B) and quantification of engraftment for each screen (C) (primary screen, left panels; secondary screens, middle and right panels) is shown. Total reconstitution of peripheral blood cells (PBC) and contribution of donor-derived, transduced (CD45.2) Lin- cells to Gr1+, CD19+ (B cells) and CD3+ (T cells) in the blood (C, upper panels), BM (total BM cells, Lin-, cKit+ cells and LSK cells)(C, middle panels), and to splenic CD43- and B cells (CD43-, CD19+, CD220+)(C, bottom panels) in recipient mice at 4 wpt is shown. Error bars represent ± SEM.

For isolation of resting B cells from spleen single cell suspension, CD43 positive cells were first depleted using anti-mouse CD43 (Ly-48). Donor-derived cells were then phenotypically analyzed and isolated by FACS sorting using fluorochrome-labeled antibodies.

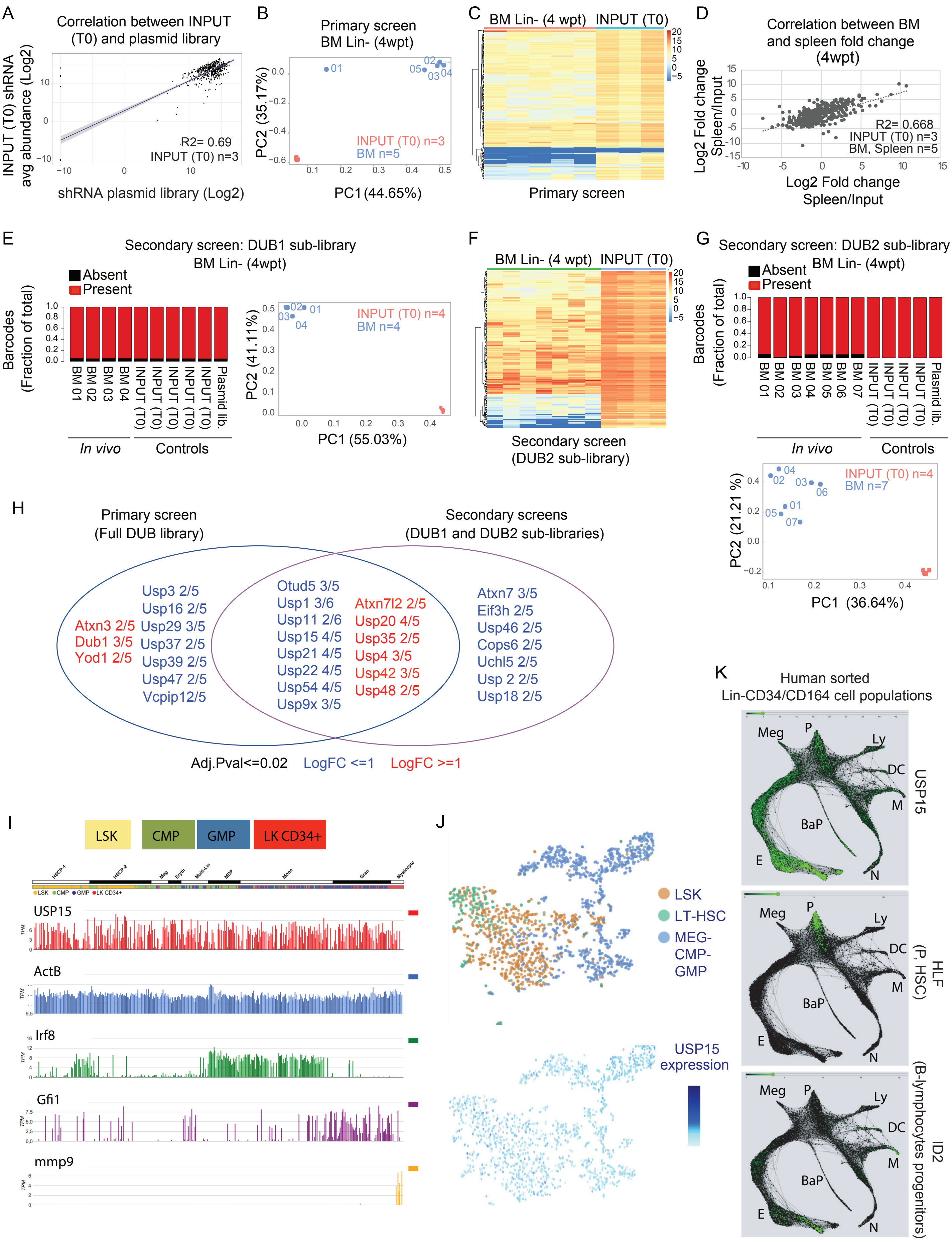

Figure S2. Pooled *in vivo* RNAi screens identify candidates DUBs effectors of HSC activity, Related to Figure 1.

Figure S2. Pooled *in vivo* RNAi screens identify candidates DUBs effectors of HSC activity, Related to Figure 1.

(A-D) Primary screen, full DUB library.

(A) shRNAs library complexity is maintained in transduced Lin- cells. Pairwise correlation between the shRNA-seq profiles of the plasmid DNA (plasmid library) used to generate the viral pool and the genomic DNA isolated from Lin- cells infected with the same viral pool prior transplantation, INPUT (T0).

(B) PC analysis of *in vivo* Lin- cells harvested from recipient mice at 4 wpt and INPUT (T0) samples.

(C) Quantitative heat map depiction of the primary screen with individual shRNAs (in rows) in each replicate (column) shown in red for the highest number of reads and in blue for lowest numbers of reads.

(D) Scatter plot of the change in shRNA representation after *in vivo* growth shows good correlation between the BM and the spleen compared to the input. Person's r is indicated.

(E) Secondary screen: DUB1 sub-library. Left panel: consolidated fraction of shRNAs retrieved *in vivo* in BM Lin- cells at 4 wpt and in controls. Right panel: PC analysis of *in vivo* and INPUT (T0) samples.

(F) Quantitative heat map depiction of the secondary screen (DUB2 sub-library) with individual shRNAs (in rows) in each replicate (column) shown in red for the highest number of reads and in blue for lowest numbers of reads.

(G) Secondary screen: DUB2 sub-library. Upper panel: consolidated fraction of shRNAs retrieved *in vivo* in BM Lin- cells at 4 wpt and in controls. Lower panel: PC analysis of *in vivo* and INPUT (T0) samples.

(H) Classification of screen results depicting significantly differentially represented DUBs overlapping between the primary and secondary screens. Candidate genes targeted by at least two shRNAs were selected with the indicated criteria. Genes were not scored if multiple cognate hairpins showed opposing effects. The number of differentially represented shRNAs per gene is indicated.

(I-K) USP15 is highly and homogeneously expressed in the hematopoietic lineage tree at single cell level.

(I) scRNA-Seq on stem/multipotent progenitors (LSK; lin−Sca1+c-Kit+), common myeloid progenitors (CMP), granulocyte monocyte progenitors (GMP), and LK CD34+ cells (lin−c-Kit+CD34+) that included granulocytic precursors. HSCP-1, hematopoietic stem cell progenitor; HSCP-2; Meg, megakaryocytic; Eryth, erythrocytic; Multi-Lin*, multi-lineage primed; MDP, monocyte-dendritic cell precursor; Mono, monocytic; Gran, granulocytic; and myelocyte, myelocytes and metamyelocytes. EndFragment. Mouse Expression (Olsson et al., 2016).

(J) scRNA-seq on HSC/hematopoietic progenitors. LSK, hematopoietic stem cells and hematopoietic multipotent progenitors; LT-HSC, long-term hematopoietic stem cells (Lin–c-Kit+Sca1+CD34–Flk2–); MEP-CMP-GMP, megakaryocyte-erythroid progenitor cells (MEP), common myeloid progenitors (CMP), and granulocyte-monocyte progenitors. Mouse Expression (Nestorowa et al., 2016).

(K) USP15 is expressed in single cells human CD34+ HSC and progenitors.

Analysis of USP15 expression in scRNA-seq data set of the human Lin−compartment, as investigated by means of CD34/CD164 fractionation (Pellin et al., 2019). The panels represent a SPRING plot of sorted human Lin−CD34/CD164 single-cell transcriptomes. Each point is one cell. Labels at the edges represent the transcriptional states associated to early lineage commitment

(P, early progenitor cells; Meg, megakaryocytes; E, erythroid cells; BaP, basophilprogenitors;N, neutrophils; M, monocytes; DC, dendritic cells; Ly-T/B/NK, lymphoid T/B/NK cells) (Pellin et al., 2019). Plots of USP15 expression and of the lineage defining genes HLF (P, HSC) and ID2 (B-lymphocytes progenitors branch) (LRT adjusted pvalue < 0.05) is shown in each panel.

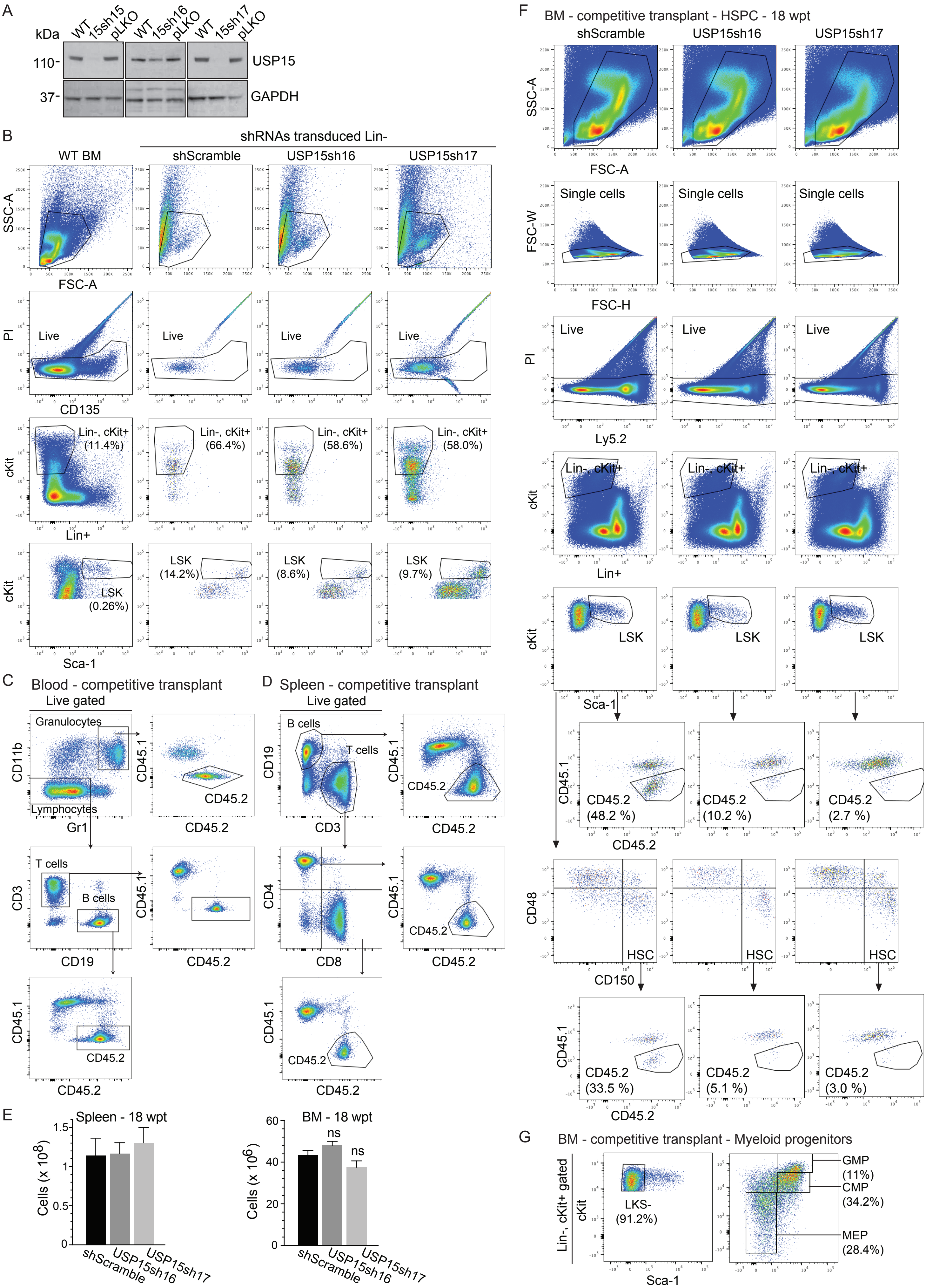

Figure S3. Hairpin validation, flow cytometry analysis and gating strategies for BM transplantation assays, Related to Figure 2 and Figure 4A, B.

Figure S3. Hairpin validation, flow cytometry analysis and gating strategies for BM transplantation assays, Related to Figure 2 and Figure 4A, B.

(A) Wild type (WT) KPE primary mouse NSCLC cells (Serresi et al., 2016) were infected with empty lentiviral vector (pLKO) or USP15-targeting (shUSP15) shRNAs expressing lentiviruses and puromycin-selected for 48hrs. The knockdown efficiency of USP15 was detected by immunoblot. Related to Figure 2B.

(B) Flow cytometry analysis of Lin- cells transduced with the indicated shRNA and grown *in vitro*. Full FACS representative profiles and frequency of Lin-, cKit+ and of LSK per live cells are shown (related to Figure 2C).

(C, D) Gating strategy for blood and spleen subsets analysis in bone marrow transplantation assays (related to Figure 2E-G and to Figure 4A, B).

(E-G) Freshly isolated wt murine Lin- cells were transduced with shRNAs targeting USP15 or control shRNA and assayed in competitive bone marrow transplantation.

(E) Total number of cells in the bone marrow (BM, left panel) and in the spleen (right panel) of recipient mice at 18 wpt is shown (related to Figure 2G and 2H-K).

P-value was assessed by Student's t test in Prism 7. ns, non significant. Data represent mean values ± SEM. N=3 per shRNA, except for shUSP15#16, n=4.

(F) Full gating strategy and representative FACS profiles for hematopoietic stem and progenitor compartment analysis (related to Figure 2H-J). Within the lineage-, Sca1+, c-Kit+ (LSK) population, cells were gated based on CD48 and CD150 to identify HSC (Cabezas-Wallscheid et al., 2014; Kiel et al., 2005). Percentages of the CD45.2 positive cells in the parental cell populations (LSK) or (HSC) are shown.

(G) Gating strategy for myeloid progenitors (related to Figure 2K). Within the lineage-, c-Kit+, Sca1- (LKS-) population, cells were gated based on CD16/32 and CD34 expression to identify common myeloid progenitors (CMP), granulocyte monocyte progenitors (GMP) and megakaryocyte erythrocyte progenitors (MEP). Frequency in the parental cell populations is shown.

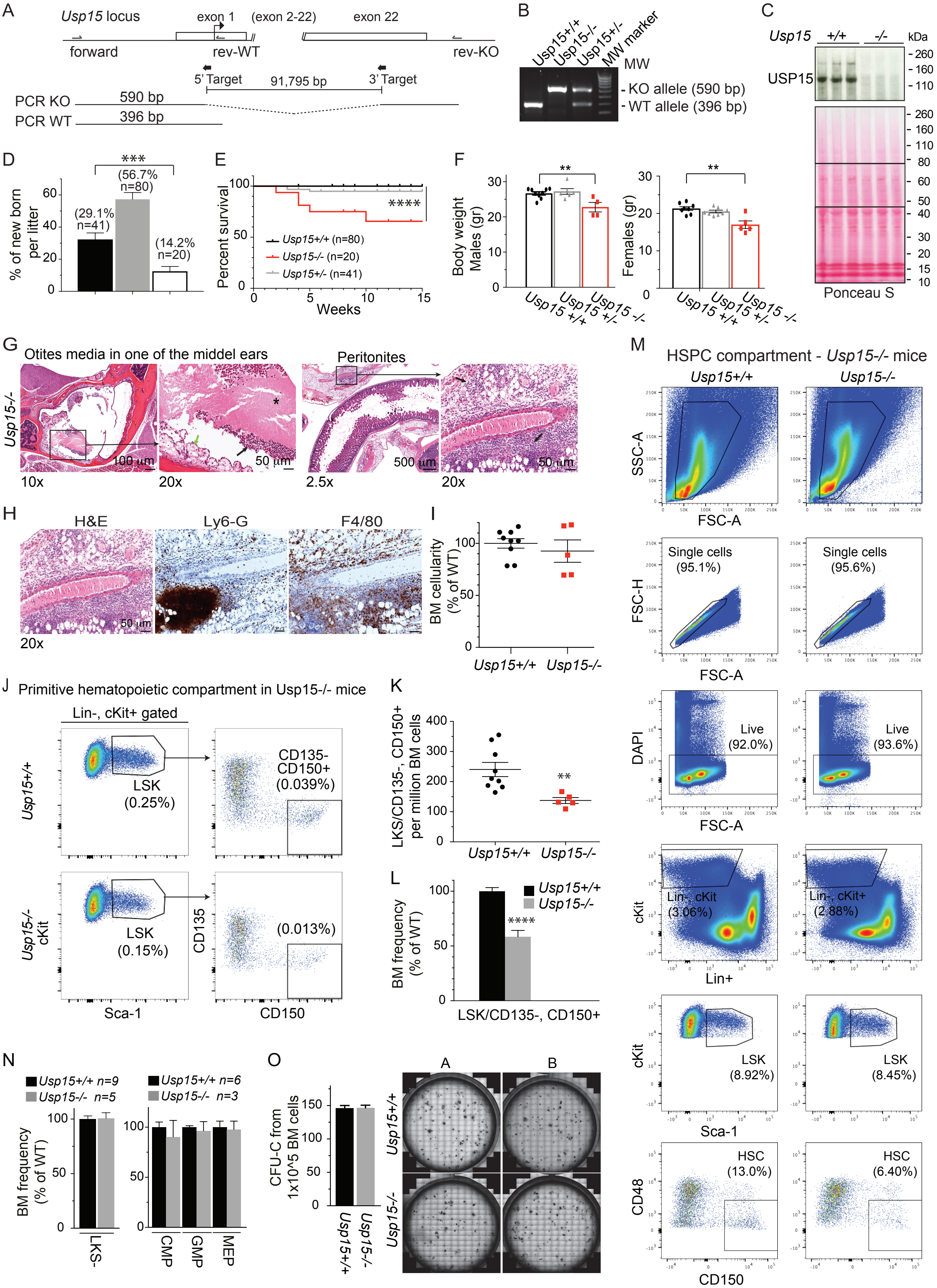

Figure S4. Generation and initial characterization of *Usp15* knockout mouse model, Related to Figure 3.

Figure S4. Generation and initial characterization of *Usp15* knockout mouse model, Related to Figure 3.

(A) Schematic representation of the strategy for germline deletion of the entire mouse *Usp15* locus (91,795 bp) by CRISPR/Cas9. On top: wild-type *Usp15* genomic locus. Oligos (arrows) for PCR screening are indicated. Middle: targeting primers positioned at the transcriptional start site within exon 1 (5' Target) and after the PolyA signal in exon 22 (3' Target). Bottom: PCR fragments corresponding to the *Usp15* knockout (KO) or wild-type (wt) allele.

(B) PCR analysis of genomic tail tip DNA.

(C) Immunoblot of whole cell extract from freshly isolated BM cells form *Usp15+/+* (n=3) and *Usp15-/-* (n=3) mice with anti-USP15 antibody. Total proteins staining with Ponceau S is shown (lower panel).

(D) Genotype frequency per litter, on a total of 31 litters. n = number of born mice/genotype.

(E) Kaplan-Meier survival analysis.

(F) Body weight of mice of the indicated genotype. Males, 8-12 wks old. Females, 8-14 wks old.

(G) Histopathological analysis of *Usp15-/-* mice. Full pathological analysis was performed on 6 *Usp15+/+* and 5 *Usp15-/-* mice between 10 and 12 weeks of age. 3 out of 5 *Usp15-/-* animals showed inflammatory lesions, in one case severe (bottom). Top: representative H&E-stained head from a *Usp15-/-* mouse (11 wks) showing lesions of otitis media in one of the inner ears. Proteaceous material (*) with dead cells and inflammatory cells (black arrow) is visible, as well as edema (green arrow). Bottom: Representative H&E-stained intestine from a *Usp15-/-* mouse (11 wks) showing severe inflammatory lesions with massive immune cells infiltration in the mesentery (black arrows). No lesions were found in *Usp15+/+* littermates (not shown).

(H) Immunohistochemistry (IHC) of Ly6-G and F4/80 confirmed the nature of multiple inflammatory lesions encountered in the skin and abdominal cavity of the mouse (case n.1 in Table S7). As shown in the representative microphotographs of mesentery stained by H&E (Hematoxylin/Eosin) and IHC, the cells involved in the massive inflammatory infiltrations were mainly those neutrophils and macrophages that were labeled by Ly6-G and F4/80, respectively. The inflammation was accompanied by multiple necrotic changes. Thereby a nature of acute purulent inflammation is evident. Bar: 50 μm.

(I) Bone marrow was isolated from femurs of *Usp15+/+* (n=9) and *Usp15-/-* (n=5) 8-12 wks old mice. The number of total BM live cells was calculated and normalized to *Usp15+/+* littermates.

(J-O) Hematopoietic primitive compartment in USP15 knockout mice (relative to Figure 4).

(J-N) Multiparameter flow cytometry analysis of the hematopoietic primitive populations in *Usp15+/+* and *Usp15-/-* mice (8-12 wks old).

(J) Gating strategy with representative FACS profiles using CD135/Flk-2 and CD150 surface markers to assess immature precursors endowed with reconstitution potential (LSK, CD135-, CD150+)(Christensen and Weissman, 2001; Kiel et al., 2005; Yeung and Wai Eric So, 2009).

(K) LSK, CD150+, CD135- cell numbers per million live BM cells.

(L) Frequency of LSK, CD150+, CD135- immature precursors in bone marrow of *Usp15-/-* mice was calculated and normalized to *Usp15+/+* animals (*Usp15+/+* n=9, *Usp15-/- n=5*).

(M) Flow cytometry analysis and representative FACS profiles for hematopoietic stem and progenitor compartment in 8-12 wks old *Usp15+/+* and *Usp15-/-* mice (related to Fig. 3).

(N) BM frequency of the indicated progenitor populations in *Usp15-/-* mice normalized to Usp15+/+ animals. Results are from three (K, L, and M left panel) (*Usp15+/+* n=9, *Usp15-/- n=5*) or two (M, right panel) independent experiments.

(O) BM cells from wt or *Usp15*-/- mice were assayed for myeloid colony-forming (CFU-C) ability. Results are the mean ± SEM from two independent experiments, *n* = 3 per genotype per experiment. Representative images of two technical replica (A, B) are shown.
For all panels: **, P ≤ 0.01; ***, P ≤ 0.001; ****, P ≤ 0.0001. P-value was assessed by Student's *t* test (D, F, K, L) or by Log-rank test (E). Mean ± SEM is shown.

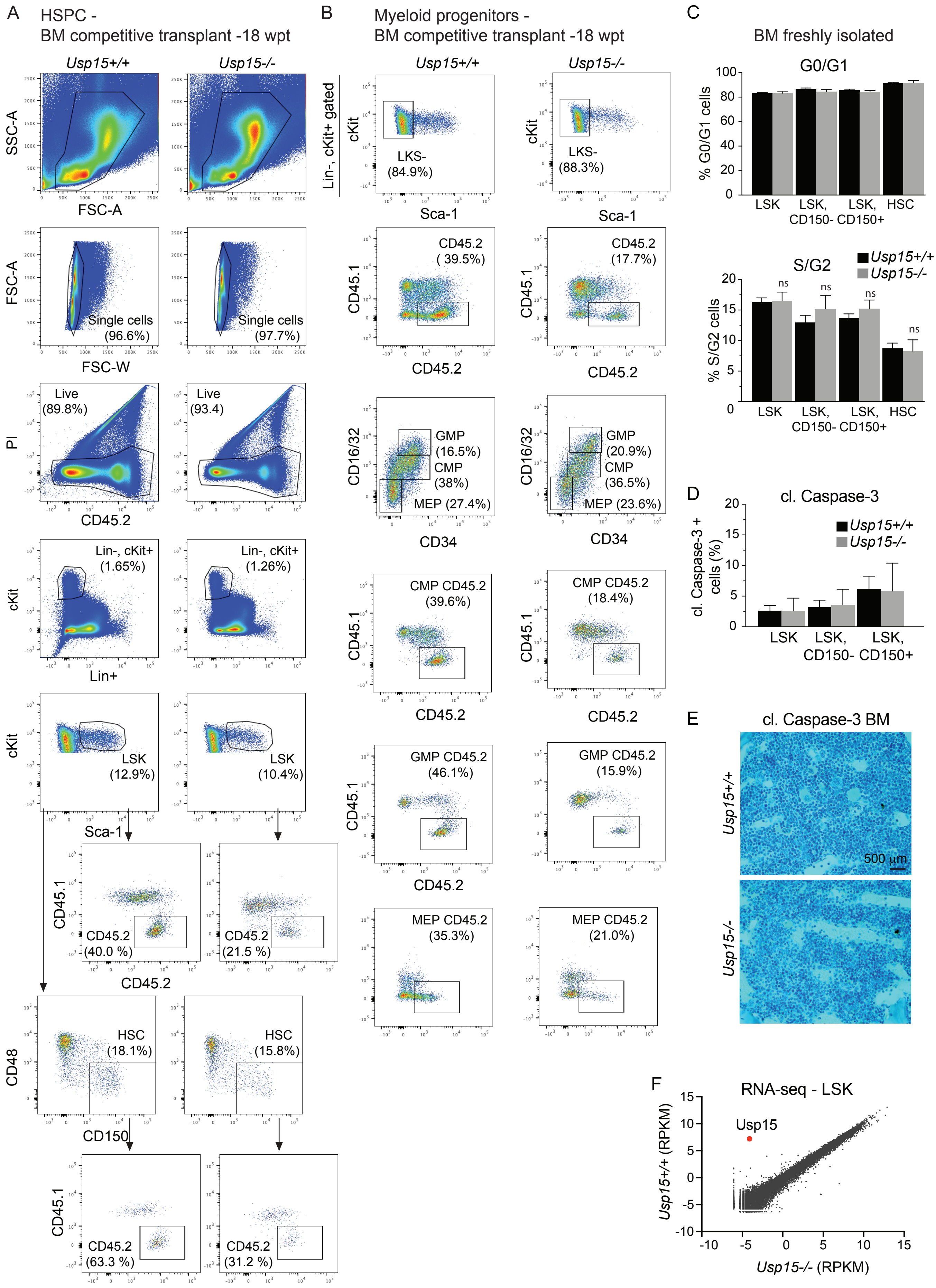

Figure S5. BM transplantation and *Usp15+/+* and *Usp15-/-* mice hematopoiesis, Related to Figure 4.

Figure S5. BM transplantation and *Usp15+/+* and *Usp15-/-* mice hematopoiesis, Related to Figure 4.

(A, B) BM analysis, HSPC subsets (A) and myeloid progenitors (B) in competitive BM transplantation primary recipient mice at 18 wpt (related to Fig. 4). Frequency in the parental cell populations is shown.

(C) Cell cycle analysis of hematopoietic stem and progenitor cells populations in wt and USP15 knockout mice. Percentage of G0/G1 and S/G2 cells in *Usp15+/+* and *Usp15-/-* mice was determined by DAPI analysis in 12-16 weeks old mice. *Usp15+/+*, n=10; *Usp15-/-*, n=5. Results are the mean ± SEM of three independent experiments.

(D) Cleaved (cl.) Caspase-3 analysis of freshly isolated hematopoietic stem and progenitor cells in *Usp15*+/+ and *Usp15*-/- mice. Results are the mean ± SEM of two independent experiments. *Usp15+/+*, n=6; *Usp15-/-*, n=2. 15-20 weeks old mice.

(E) Representative images of *Usp15*+/+ and *Usp15-/-* BM sections stained for apoptosis-indicating cleavage (cl.) of Caspase-3. *n* = 6 mice per genotype. Bar, 500 µm.

(F) RNA-seq of LSK population freshly isolated from BM. Comparison of normalized gene expression data for *Usp15*-/- (n-=2) versus *Usp15*+/+ (n=3) LSK (2 months old mice). USP15 expression is highlighted in red.

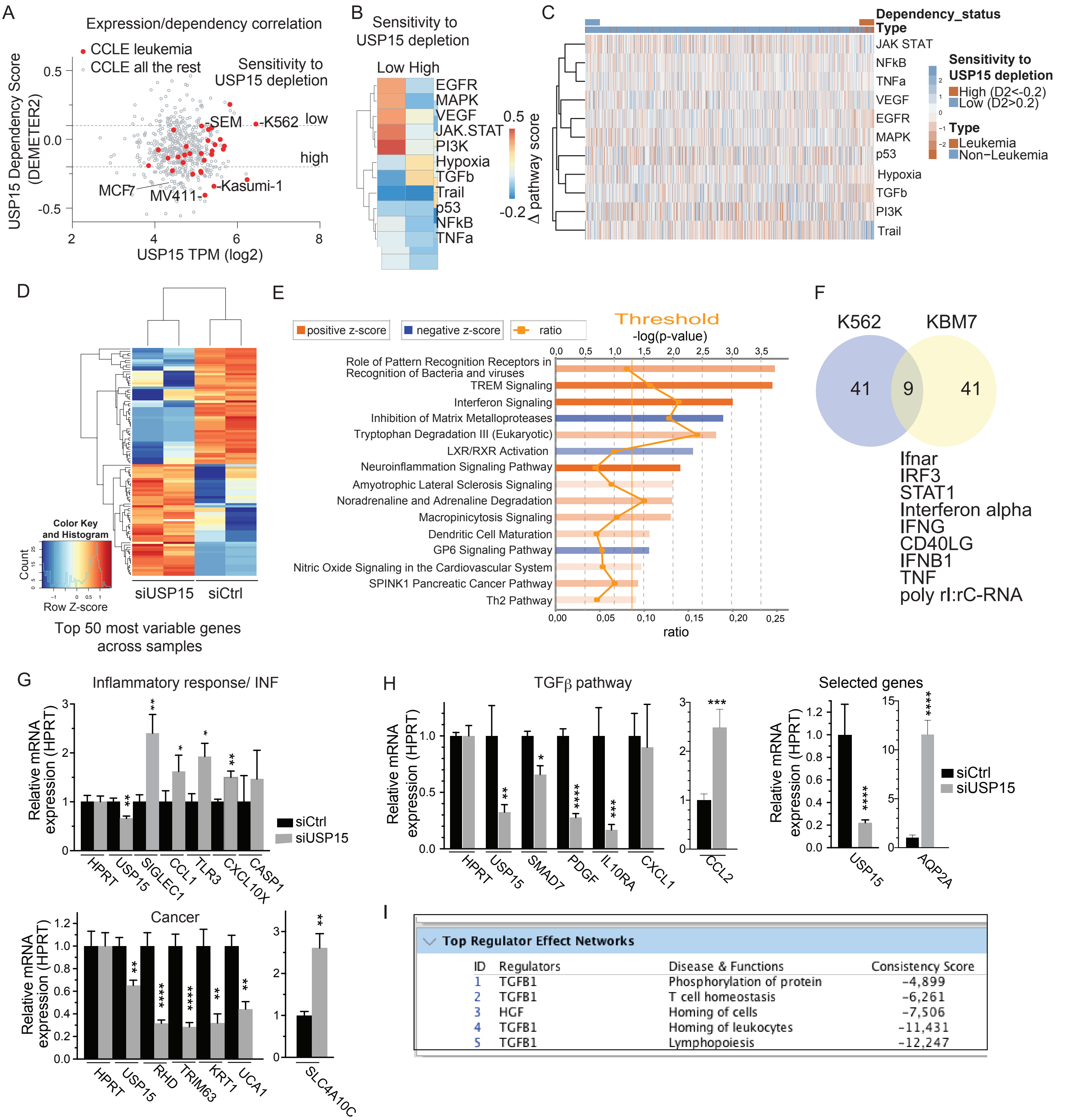

Figure S6. USP15 RNAi in CML cell lines results in alteration of inflammatory signaling pathways, Related to Figure 5.

Figure S6. USP15 RNAi in CML cell lines results in alteration of inflammatory signaling pathways, Related to Figure 5.

(A) The dependency scores were calculated by Demeter2 for USP15 RNAi in CCLE lines (McFarland et al., 2018) and correlated to USP15 gene expression levels. TPM (Transcripts Per Million).

(B) Differential pathway activation by PROGENy for cell lines with DEMETER2 scores D2<-0.2 (Low) and D2>0.2 (high).

(C) Heat map showing PROGENy scores for the indicated pathways (rows) in all the CCLE cell lines (columns). Rows are sorted by correlation whereas columns are sorted by DEMETER2 score. The legends indicate DEMETER2 score and tumor type.

(D-I) KBM7 (D, E, F, G) or K562 (F, H, I) cells were transfected with USP15 (siUSP15) or non-targeting (siCtrl) siRNA pool. At 72 hours after transfection, cells were harvested and RNA was extracted and subjected to RNAseq (D, E, F, I) or used for RT-qPCR analysis (G, H).

(D) Unbiased clustering of genes significantly dysregulated (>1 fold change and Adjusted *P* value <0.05) in KBM7 RNAseq dataset.

(E) Ingenuity pathway analysis of USP15-dependent genes in the KBM7 dataset showing dysregulated top canonical pathways (cut-off logFC > 1; adjusted P value <0.05).

(F) Venn diagram depicting the overlap between Top 50 significantly enriched Ingenuity Upstream Regulators in each cell line RNAseq datasets. The Upstream Regulators were calculated based on differential gene expression between USP15 and control siRNAs. The nine overlapping terms are indicated below.

(G) qRT-PCR analysis of the indicated genes in KBM7 USP15 knock down cells.

(H) qRT-PCR analysis of the indicated genes in K562 USP15 knock down cells.

(I) USP15 RNAi in K562 cells. Ingenuity pathway analysis of USP15-dependent genes in the K562 RNAseq dataset showing dysregulated Top Regulator Effect Networks (cut-off logFC > 1; adjusted P value <0.05).

Results are from one (KBM7) or two independent experiments (K562) per cell line. Each experiment n=2 per siRNA/ cell line. Results are mean ± SD (F, G). **. $P \leq 0.01$; ***. $P \leq 0.001$; ****. $P \leq 0.0001$. P-value was assessed by Student's *t* test.

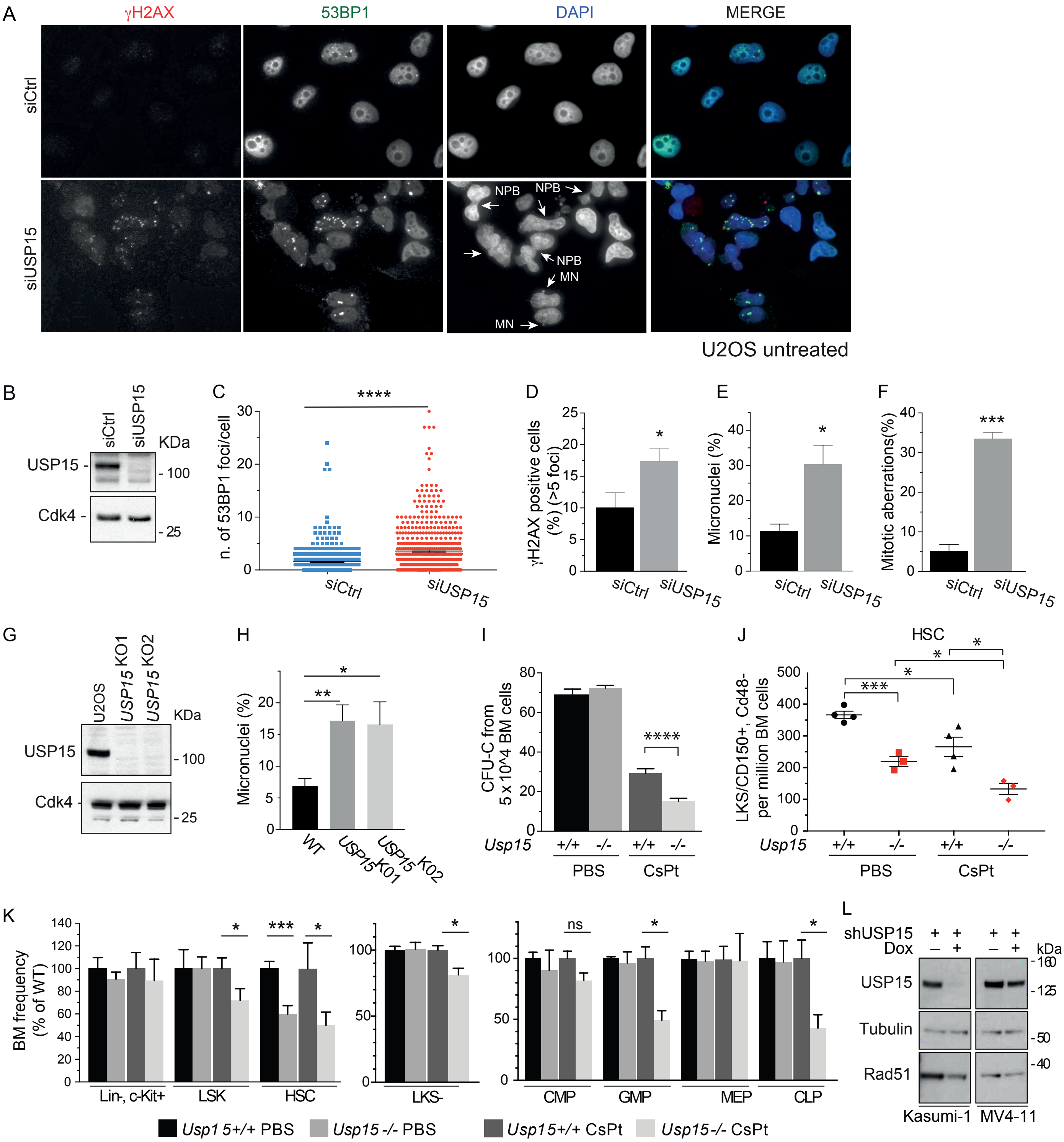

Figure S7. USP15 loss enhances genotoxic stress *in vitro* in human cancer cells and i*n vivo* in mouse hematopoietic stem and progenitor cells, Related to Figure 6.

Figure S7. USP15 loss enhances genotoxic stress *in vitro* in human cancer cells and *in vivo* in mouse hematopoietic stem and progenitor cells, Related to Figure 6.

(A-G) Knockdown or CRISPR/Cas9- mediated knockout of USP15 in U2OS cancer cells induces spontaneous DNA damage.

(A-F) U2OS cells were transfected with USP15 (siUSP15) or non-targeting (siCtrl) siRNA pool and assayed at 72 hours after transfection.

(A) Representative images of co-immunostaining for γH2AX and 53BP1. Arrows indicate micronuclei (MN), nucleoplasmatic bridges (NPB), and abnormal mitosis.

(B) Western blot with the indicated antibodies on whole cell extract.

(C) Quantification by Image J of the number of spontaneous 53BP1 foci per cell. n=4 experiments; >400 cells per genotype were in total analyzed. Mean values ± SEM are shown.

(D) Quantification of the percentage of cells containing >5 spontaneous γH2AX foci. Mean ± SD is shown.

(E) Percentage of micronuclei. Mean values ± SEM are shown.

(F) Percentage of aberrant mitosis, including bi-nucleated cells and cells with nucleoplasmatic bridges. Mean values ± SEM are shown.

(A-F) All quantifications are form three independent experiments. *n* > 50 cells/genotype/experiment.

(G-H) CRISPR/Cas9- mediated deletion of USP15 in U2OS cells.

(G) Western blot with the indicated antibodies on whole cell extract.

(H) Micronuclei were quantified in wt cells and in two independent USP15 knockout clones, in two independent experiments. A minimum of 100 cells per genotype/clone/experiment were scored. Mean values ± SEM are shown.

(I-K) USP15 protects the hematopoietic stem and progenitor compartment from genotoxic stress *in vivo*. *Usp15+/+ and Usp15-/-* mice (8-12 wks old) were injected with PBS or with low dose of cisplatin (CsPt)(0.8 mg/kg i.v.). Mice were sacrificed after 48 hrs.

(I) BM cells were isolated from wt or *Usp15*-/- mice and were assayed for myeloid colony-forming (CFU-C) ability.

(J, K) Multiparameter flow cytometry analysis of the hematopoietic stem and progenitor compartment.

(J) HSC (LSK, CD150+, CD48-) cell numbers per million live BM cells.

(K) The frequency of the indicated cell populations in *Usp15-/-* mice was calculated and normalized to *Usp15*+/+ animals. LSK/CD150+ CD48- (HSC), lineage-, c-Kit+, Sca1- (LKS-), common myeloid progenitors (CMP), granulocyte monocyte progenitors (GMP), megakaryocyte erythrocyte progenitors (MEP), common lymphoid progenitors (CLP).

Results are the mean ± SEM or SD (K, left panel) of two independent experiments. Mice: (J-K) *Usp15+/+ n* = 4, *Usp15-/- n* = 3 per treatment; (K) *n* = 3 per genotype per treatment.

(L) Kasumi-1 and MV4-11 cells harboring USP15 shRNA were kept in medium with or without doxycycline (Dox) for 5 days. Western blot was performed with the indicated antibodies. One representative experiment out of 2 is shown.

For all panels: *, P ≤ 0.05; **,P ≤ 0.01; ***, P ≤ 0.001; ****, P ≤ 0.001 as assessed by Student's t test.

Table S4. Primer sequences for Illumina sequencing multiplexing strategy, Related to Figure 1.

**PCR 1, forward primer**	*ACACTCTTTCCCTACACGACGCTCTTCCGATCT***NNNNNN**GGCTTTATATATCTTGTGGAAAGGACG
**PCR 1, reverse primer**	GTGACTGGAGTTCAGACGTGTGCTCTTCCGATCTGTGGATGAATACTGCCATTTGTCTC
**PCR 2, forward primer**	AATGATACGGCGACCACCGAGATCT*ACACTCTTTCCCTACACGACGCTCTTCCGATCT*
**PCR 2, reverse primer**	CAAGCAGAAGACGGCATACGAGAT**NNNNNN**GTGACTGGAGTTCAGACGTGTGCTCTTCCGATCT
**PCR 1 barcodes (green)**	
Barcode #1	CGTGAT
Barcode #2	ACATCG
Barcode #3	GCCTAA
Barcode #4	TGGTCA
Barcode #5	CACTGT
Barcode #6	ATTGGC
Barcode #7	GATCTG
Barcode #8	TCAAGT
Barcode #9	CTGATC
Barcode #10	AAGCTA
Barcode #11	GTAGCC
Barcode #12	TACAAG
**PCR 2 Illumina Indexes (green)**	
Index #1	ATCACG
Index #2	CGATGT
Index #3	TTAGGC
Index #4	TGACCA

PCR1 forward primer is designed to include: Illumina/Solexa adapter 1 (red, italics), barcode (green, bold and underlined), pLKO.1 common region (plain text).

Table S5. Hematopoietic stem and progenitor cell populations and their markers, Related to Figures 2, 3 and 4.

Subset	Subpopulations	Markers
Lin^-	LSK, HSC, LKS-, CMP, GMP, MEP	$Lineage^-$
LSK	HSC	$Lineage^-$, Sca-1^+, $cKit^+$
HSC		$Lineage^-$, Sca-1^+, $cKit^+$, $CD150^+$, $CD48^-$
LKS^-	CMP, GMP, MEP	$Lineage^-$, Sca-1^-, $cKit^+$
CMP		$Lineage^-$, Sca-1^-, $cKit^+$, $CD34^{int}$, $CD16/32^{int}$
GMP		$Lineage^-$, Sca-1^-, $cKit^+$, $CD34^+$, $CD16/32^+$
MEP		$Lineage^-$, Sca-1^-, $cKit^+$, $CD34^-$, $CD16/32^-$

Table S6. List of antibodies used in this study, Related to STAR Methods section.

Antigen-conjugate	Supplier	Dilution
Lineage Cell Detection Mixture-Biotin, mouse	MACS Miltenyi Biotech	1:30
Streptavidin APC/Cy7	Southern Biotech	1:200
cKit-APC	eBioscience	1:200
Sca1-PerCp-Cy5.5	Biolegend	1:200
CD48-PE-Dazzle 594	Biolegend	1:200
CD48-FITC	BD Biosciences	1:200
CD135-PE	Biolegend	1:200
CD150-PE-Cy7	Biolegend	1:200
CD16/32-PE-Cy7	eBioscience	1:200
CD34-FITC	eBioscience	1:200
CD45.1(Ly5.1)-EF450	eBioscience	1:200
CD45.2(Ly5.2)-PE	Biolegend	1:200
CD45.1(Ly5.1)-PE	BD Biosciences	1:200
CD45.2(Ly5.2)-PE-Cy7	Biolegend	1:200
CD3-FITC	Biolegend	1:200
CD19-APC	BD Biosciences	1:200
Gr1-APC-Cy7	Biolegend	1:200
CD11b-PerCp-Cy5.5	BD Biosciences	1:200
CD4-APC	Biolegend	1:200
CD8a-PerCp-C5.5	BD Biosciences	1:200
CD19-APC-H7	BD Biosciences	1:200
CD43 (Ly-48) MicroBeads, mouse	MACS Miltenyi Biotech	n.a.
CD43-biotin (clone S7, RUO)	BD Biosciences	1:100
CD19-APC	Biolegend	1:200
B220-Pacific Blue	BD Biosciences	1:200
CD45.2-FITC	BD Biosciences	1:200
γH2AX	Millipore	1:1000
53BP1	Novus Biologicals	1:500
USP15 Mab ab56900	Abcam	1:500
USP15 Rb ab71713	Abcam	1:500
FUS – NB100-565	Novus Biologicals	1:500

Tubulin	Sigma	1:10,000
Goat anti-mouse HRP	Life technologies	1:10,000
Rabbit IgG 2729S	Cell Signaling Technology	
Alexa Fluor 568 goat anti–mouse	Life Technologies	1:250
Alexa Fluor 488 goat anti–rabbit	Life Technologies	1:250

Table S7. Inflammation in *Usp15-/-* mice, Related to Figure 3 and Figure S4.

Genotype	Age	Cases	Sign of inflammation
Usp15+/+	10-12 wks	0/6	No lesions detected
Usp15-/-	10-12 wks	3/5	*Case 1:*<br>Peritonitis, Laryngitis.<br>Skin: Local purulent inflammatory lesions subcutaneously, showing massive infiltration of neutrophils accompanied by necrosis of the tissue.<br>Testicular: degeneration in parts of the testes, accompanied by severe inflammatory changes in the surrounding adipose tissues with adiponecrosis.<br>GI tract: Purulent inflammatory lesions in the mesentery accompanied by fibrosis and adherence.<br>Spleen: Enhancement of haematopoiesis in the red pulp.<br>Pancreas: Edematous changes with inflammatory infiltrations in stroma.<br>Kidney: Purulent inflammatory lesions in the renal capsules and in the surrounding adipose tissues.<br>Gall bladder: Edematous changes in the submucosa with inflammatory lesions in the surroundings.<br>Head: Edema and inflammatory infiltrations in the submucosa of larynx.<br>Spleen: Enhancement of hematopoiesis in the red pulp of spleen<br><br>*Case 2:*<br>Head: -Local purulent inflammatory lesions in the skin of lip.<br>- Otitis media in one of the middle ears.<br>-Uterus/ovary Edematous changes of the endometrial stroma.<br><br>*Case 3:*<br>Head: Lesions of otitis media in one of the middle ears.

Histopathological analysis of *Usp15-/-* mice. Full pathological analysis was performed on 6 *Usp15+/+* and 5 *Usp15-/-* mice between 10 and 12 weeks (wks) of age. GI: gastro intestinal tract.

Table S8. TCGA Pan-Cancer (PANCAN) samples, related to Figure 5A, B and D.

PANCAN EXPRESSION analysis (A,B)			PANCAN survival analysis (C)	
acute myeloid leukemia	173		acute myeloid leukemia	161
adrenocortical cancer	79		adrenocortical cancer	79
bladder urothelial carcinoma	427		bladder urothelial carcinoma	426
brain lower grade glioma	530		brain lower grade glioma	528
breast invasive carcinoma	1218		breast invasive carcinoma	1214
cervical & endocervical cancer	310		cervical & endocervical cancer	309
cholangiocarcinoma	45		cholangiocarcinoma	45
colon adenocarcinoma	495		colon adenocarcinoma	491
diffuse large B-cell lymphoma	48		diffuse large B-cell lymphoma	48
esophageal carcinoma	196		esophageal carcinoma	196
glioblastoma multiforme	172		glioblastoma multiforme	166
head & neck squamous cell carcinoma	566		head & neck squamous cell carcinoma	565
kidney chromophobe	91		kidney chromophobe	89
kidney clear cell carcinoma	606		kidney clear cell carcinoma	606
kidney papillary cell carcinoma	323		kidney papillary cell carcinoma	322
liver hepatocellular carcinoma	423		liver hepatocellular carcinoma	422
lung adenocarcinoma	576		lung adenocarcinoma	567
lung squamous cell carcinoma	553		lung squamous cell carcinoma	546
mesothelioma	87		mesothelioma	86
ovarian serous cystadenocarcinoma	308		ovarian serous cystadenocarcinoma	307
pancreatic adenocarcinoma	183		pancreatic adenocarcinoma	183
pheochromocytoma & paraganglioma	187		pheochromocytoma & paraganglioma	187
prostate adenocarcinoma	550		prostate adenocarcinoma	550
rectum adenocarcinoma	171		rectum adenocarcinoma	170
sarcoma	265		sarcoma	265
skin cutaneous melanoma	474		skin cutaneous melanoma	458
stomach adenocarcinoma	450		stomach adenocarcinoma	443
testicular germ cell tumor	156		testicular germ cell tumor	139
thymoma	122		thymoma	121
thyroid carcinoma	572		thyroid carcinoma	572
uterine carcinosarcoma	57		uterine carcinosarcoma	57
uterine corpus endometrioid carcinoma	567		uterine corpus endometrioid carcinoma	553
uveal melanoma	80		uveal melanoma	80
Total number of samples	11060			10951
